# Understanding the Electronic Structure Basis for N_2_ Binding to FeMoco: A Systematic Quantum Mechanics/Molecular Mechanics Investigation

**DOI:** 10.1021/acs.inorgchem.2c03967

**Published:** 2023-03-29

**Authors:** Yunjie Pang, Ragnar Bjornsson

**Affiliations:** † College of Chemistry, Beijing Normal University, Beijing 100875, China; ‡ Max-Planck Institute for Chemical Energy Conversion, Stiftstrasse 34-36, Mülheim an der Ruhr 45470, Germany; § Univ. Grenoble Alpes, CNRS, CEA, IRIG, Laboratoire de Chimie et Biologie des Métaux, 17 Rue des Martyrs, Grenoble F-38054, Cedex, France

## Abstract

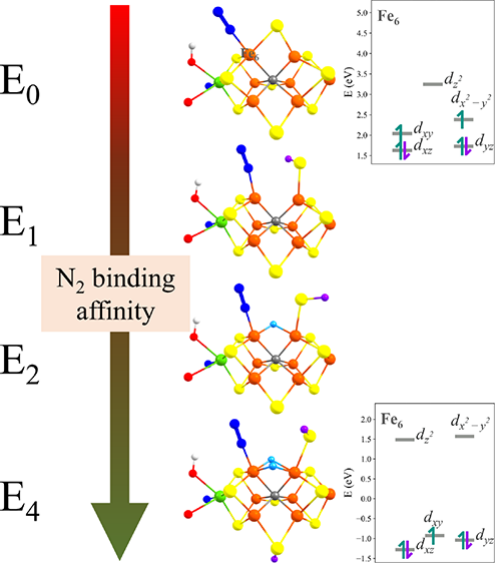

The FeMo cofactor (FeMoco) of Mo nitrogenase is responsible for reducing dinitrogen to ammonia, but it requires the addition of 3–4 e^–^/H^+^ pairs before N_2_ even binds. A binding site at the Fe2/Fe3/Fe6/Fe7 face of the cofactor has long been suggested based on mutation studies, with Fe2 or Fe6 nowadays being primarily discussed as possibilities. However, the nature of N_2_ binding to the cofactor is enigmatic as the metal ions are coordinatively saturated in the resting state with no obvious binding site. Furthermore, the cofactor consists of high-spin Fe(II)/Fe(III) ions (antiferromagnetically coupled but also mixed-valence delocalized), which are not known to bind N_2_. This suggests that an Fe binding site with a different molecular and electronic structure than the resting state must be responsible for the experimentally known N_2_ binding in the **E_4_** state of FeMoco. We have systematically studied N_2_ binding to Fe2 and Fe6 sites of FeMoco at the broken-symmetry QM/MM level as a function of the redox-, spin-, and protonation state of the cofactor. The local and global electronic structure changes to the cofactor taking place during redox events, protonation, Fe–S cleavage, hydride formation, and N_2_ coordination are systematically analyzed. Localized orbital and quasi-restricted orbital analysis via diamagnetic substitution is used to get insights into the local single Fe ion electronic structure in various states of FeMoco. A few factors emerge as essential to N_2_ binding in the calculations: spin-pairing of *d_xz_* and *d_yz_* orbitals of the N_2_-binding Fe ion, a coordination change at the N_2_-binding Fe ion aided by a hemilabile protonated sulfur, and finally hydride ligation. The results show that N_2_ binding to **E_0_**, **E_1_**, and **E_2_** models is generally unfavorable, likely due to the high-energy cost of stabilizing the necessary spin-paired electronic structure of the N_2_-binding Fe ion in a ligand environment that clearly favors high-spin states. The results for models of **E_4_**, however, suggest a feasible model for why N_2_ binding occurs experimentally in the **E_4_** state. **E_4_** models with two bridging hydrides between Fe2 and Fe6 show much more favorable N_2_ binding than other models. When two hydrides coordinate to the same Fe ion, an increased ligand-field splitting due to octahedral coordination at either Fe2 or Fe6 is found. This altered ligand field makes it easier for the Fe ion to acquire a spin-paired electronic structure with doubly occupied *d_xz_* and *d_yz_* orbitals that backbond to a terminal N_2_ ligand. Within this model for N_2_ binding, both Fe2 and Fe6 emerge as possible binding site scenarios. Due to steric effects involving the His195 residue, affecting both the N_2_ ligand and the terminal SH^–^ group, distinguishing between Fe2 and Fe6 is difficult; furthermore, the binding depends on the protonation state of His195.

## Abstract 2

The electronic structure changes associated with N_2_ binding to different redox states of FeMoco are analyzed in this work. Favorable N_2_ binding is found to correlate with local low-spin states of Fe ions, especially in redox states with bound hydrides.

## 1 Introduction

**Figure 1:**
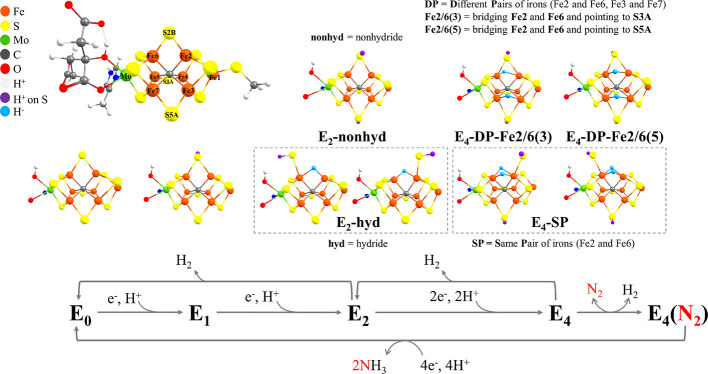
Simplified Lowe–Thorneley catalytic cycle of nitrogenase with structural models for the **E_0_**,^10^**E_1_**,^11^**E_2_**,^12^ and **E_4_**(13−15) redox states of FeMoco shown, currently discussed in the literature.

Nitrogenase is the only type of enzyme capable of reducing dinitrogen gas to ammonia at ambient temperature and pressure and has been studied for decades.^1−5^ The reaction has been proposed to follow the following stoichiometry:
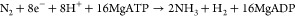
1where in addition to the 2 NH_3_ molecules, a molecule of H_2_ is formed. This obligatory H_2_ formation is now confidently known to be related to a critical H_2_ elimination step involving hydrides during the reaction. Extensive kinetic studies^6−8^ by Lowe and Thorneley (LT) led to a pioneering kinetic scheme that is still used to discuss the mechanism of the reaction. A simplified LT cycle is presented in Figure 1, where reduced intermediates are named **E_*n*_**, where the subscript *n* refers to the additional electrons/protons relative to the resting state (**E_0_**). A recent study^9^ discusses a steady-state kinetic model that establishes even more clearly the rate constants of several critical steps: the rate of H_2_ revolution from the **E_2_** state was, e.g., found to be lower than the more reduced **E_4_** state, while H_2_ formation from **E_4_** and N_2_ binding to the **E_4_** state have equal rate constants. A previously unidentified slow process was also found, believed to be related to an N_2_ activation process.

The FeMo cofactor (FeMoco) of the more active Mo-dependent nitrogenase is generally accepted as the site where N_2_ conversion to NH_3_ takes place.^7,16,17^ FeMoco contains eight open-shell metal ions (one Mo and seven Fe ions), with a complex electronic structure featuring antiferromagnetic coupling of high-spin Fe^2+/3+^ ions, mixed-valence delocalization, and a strange non-Hund electron configuration at the Mo^3+^ site.^18^ The resting **E_0_** state has a spin of S = 3/2, and a charge of −1 (of the [MoFe_7_S_9_C] part) has been proposed as the most likely redox state based on calculations^10,19^ and experiments.^20^ Together with the previous Mo^3+^ assignment,^18^ this suggests a combined Mo^3+^3Fe^2+^4Fe^3+^ oxidation state, although calculations indicate that the electronic structure can be rather delocalized.^10,19^

Broken-symmetry density functional theory remains the primary computational methodology utilized to study the electronic structure and reactivity of the system. Ten broken-symmetry (BS) solutions of FeMoco were originally suggested based on approximate *C*_3_ symmetry of the cofactor and where only Fe ions were considered as part of the magnetic coupling.^21−23^ These solutions become 35 if symmetry is ignored. The most favorable BS7 class of solutions consist of the BS7–235, BS7–247, and BS7–346 solutions (abbreviated as BS235, BS247, and BS346 from now on) where the three numbers indicate which Fe ions are spin-down (X-ray structure numbering). These three solutions cannot be distinguished energetically (being within ∼1–2 kcal/mol of each other) but they do lead to distinct geometric Fe–Fe/Mo–Fe distances, with the BS235 solution giving the best agreement with the high-resolution X-ray structure.^10^ Localized orbital analysis^10^ of the cofactor indicates that these three BS7 solutions differ in their locations of mixed-valence pairs and localized Fe^2+^ or Fe^3+^ ions, which explains the specific geometric effects behind these almost isoenergetic solutions.

The integer-spin **E_1_** state of FeMoco has an additional e^–^/H^+^ pair and is likely *S* = 1 or 2.^24^ A recent Mo,Fe XAS and Mössbauer study^25^ suggests that Fe-based reduction has occurred, while a joint Mo,Fe-EXAFS-QM/MM study^11^ further suggests that the added proton is likely on either the bridging belt sulfide S2B or S5A. The calculations showed a preference for a *M*_S_ = 2 spin state and the Fe reduction was found to be localized in the MoFe_3_ subcubane. The S2B sulfide, bridging Fe2 and Fe6 (shown in Figure 1), appears most likely as the protonation site in **E_1_**, as X-ray crystallography^26−28^ has revealed various structures indicating the lability of this sulfide position and computational studies have also suggested protonation of S2B in the **E_1_** state.^29,30^

The **E_2_** state is known to possess an *S* = 3/2 spin state with two conformers present with distinct *g*-tensors (*g* = [4.21, 3.76, ∼1.97] and [4.69, ∼3.20, ∼2]) and a hydride likely present in at least one of them.^31−34^ In a recent study^12^ from our group, we proposed two models for the **E_2_** state: **E_2_-hyd** and **E_2_-nonhyd**. One contains a bridging hydride between Fe2 and Fe6 as well as a terminal sulfhydryl group (labeled **E_2_-hyd** here). The **E_2_-hyd** with a terminal SH^–^ on Fe6, named as **E_2_-hyd-SH^–^@Fe6**, is more stable than the one with a SH^–^ on Fe2, as also seen in a recent QM/MM study.^35^ The **E_2_-nonhyd** model contains a doubly reduced Fe environment and two protons located on S2B and S5A. Both models are shown in Figure 1. As discussed both in ref (12) and more recently in ref (35), **E_2_** isomers are, however, quite sensitive to the DFT method used with TPSS, e.g., giving opposite trends to r^2^SCAN and TPSSh. A saddlepoint leading to H_2_ formation from **E_2_-hyd** → **E_0_** was also reported in ref (12), with a fairly high activation barrier of ∼20 kcal/mol, which is consistent with a recent kinetics study,^9^ indicating the **E_2_** → **E_0_** step to be slower than other H_2_ formation pathways.

Little is unfortunately known experimentally about the **E_3_** state (with even few computational studies^29,30^ dedicated to it) due to its EPR-silent nature and it will not be discussed further here. In contrast to the EPR-silent **E_3_**, the EPR-active **E_4_** state is much better characterized. Extensive EPR studies have revealed a spin-state of *S* = 1/2 in both the α-70^Val → lle^ mutant and native enzyme.^36,37^ ENDOR studies have revealed that the state contains two bridging hydrides, bridging between the Fe ions (Fe-H-Fe) with two other protons bound to sulfides.^36,38^ A complete structural model for **E_4_** remains controversial, however. The first proposed models based on the ENDOR data suggested hydrides bridging Fe ions (and protonated belt sulfides) without any associated structural changes suggested.^39^ Computational studies have suggested models with either bridging or terminal hydrides as energetically favorable states.^13,15,30,38^ Some of these models feature hydrides bridging different pairs (**DP**) of Fe ions (Fe2 and Fe6, Fe3 and Fe7) with closed belt sulfide bridges and were shown to be in good agreement with ^1^H hyperfine tensor orientations,^15,38^ here labeled **E_4_-DP-Fe2/6(5)** and **E_4_-DP-Fe2/6(3)**, where **Fe2/6(5)** and **Fe2/6(3)** labels indicate that the hydrides bridging Fe2 and Fe6 points to either **S5A** and **S3A**, respectively. **E_4_-DP-Fe2/6(3)** was calculated to be lower in energy than **E_4_-DP-Fe2/6(5)**, but only the latter agreed with ENDOR results.^15^ A QM/MM study (using the TPSSh functional) from our research group in contrast suggested **E_4_** models with two bridging hydrides located on the same pair (**SP**) of Fe ions (Fe2 and Fe6) and with open belt sulfur bridges (terminal sulfhydryl groups) that were found to be energetically more favorable than models with closed belt sulfur bridges (including **E_4_-DP-Fe2/6(5)**). Such opening of protonated belt sulfides has also been discussed in computational work by Dance,^40^ Raugei,^13^ and recently by Ryde and co-workers.^35^

Kinetic and spectroscopic studies^7,37,41−44^ indicate that dinitrogen will bind to the **E_4_** state, while no binding has been directly observed in earlier **E_*n*_** states (with the role of **E_3_** unclear). Alternative substrates and inhibitors like hydrazine, diazene, acetylene, and CO likely bind on the other hand to the **E_1_** or **E_2_** states.^4,5^ The Fe2-Fe3-Fe6-Fe7 face has been proposed as a likely site for N_2_ binding based on mutation studies^45,46^ of α-70^Val^ where the N_2_ reduction is dramatically decreased, with the Fe2 and Fe6 sites being the primary possibilities. Unfortunately, experimental characterization of dinitrogen-bound intermediates is scarce and the nature of the molecular and electronic structure of the cofactor prior to and after dinitrogen binding is unclear. Additionally, crystallographic studies^26−28^ have shown that the bridging sulfide (S2B) between Fe2 and Fe6 can be displaced under certain conditions, implicating Fe2 and Fe6 as a site of ligand binding, but which could also be interpreted as sulfide lability being mechanistically relevant.^47,48^ A recent study presenting an X-ray crystal structure^28^ apparently showing a missing sulfide and bound N_2_ is controversial.^49−51^ Some recent computational studies have investigated the protonation and dissociation of S2B and the mechanism of N_2_ reduction after S2B dissociation, but it remains unclear whether S2B loss is part of the catalytic mechanism or not.^52−56^ Other computational studies^13,57−60^ have investigated N_2_ binding to the whole cofactor; however, N_2_ binding is rarely found to bind favorably to FeMoco. In a previous QM/MM study^14^ from our group, however, we found that **E_4_-SP** type cofactor models will favorably bind N_2_.

H_2_ evolution has for a long time been known to be a compulsory mechanistic step when reducing dinitrogen.^7^ Unlike the unproductive side-reactions **E_2_** → **E_0_** and **E_4_** → **E_2_** (that proceed via a hydride-protonation, *hp*, mechanism^37,38^) the obligatory H_2_ formation (the H_2_ formation part of eq 1) is known to occur via another mechanism, typically described as reductive elimination, *re* (though this nomenclature has also been challenged^61^). This elimination involves a direct reaction of the two bridging hydrides as N_2_ binds.^62^ N_2_ thus appears to act as a catalyst for the specific H_2_ formation via the *re* mechanism (as established via isotope labeling^37,38^). Hoffman and co-workers have classified the mechanistic possibilities for this N_2_/H_2_ reaction as “dissociative”, “concerted”, “associative”, and “intermediate”.^63^ A “dissociative” mechanism (i.e., H_2_ leaving first) can be ruled out based on the isotope labeling experiments, while “concerted” (simultaneous H_2_ dissociation and N_2_ binding), “associative” (N_2_ binding followed by H_2_ dissociation), and “intermediate” (N_2_ binds to a higher energy intermediate **E_4_** followed by H_2_ leaving) remain possible scenarios. In the “concerted” scenario, H_2_ evolution simply pays the energetic cost associated with N_2_ binding. However, it seems hard to imagine such a selective mechanistic step taking place without N_2_ binding occurring first. In the “associative” scenario, N_2_ binding causes the elimination of H_2_, presumably leading to the formation of a more stable N_2_-bound state. This hypothesis, however, implies that N_2_ binding to the cofactor has somehow become more favorable than before. Finally, in the “intermediate” scenario, H_2_ formation occurs first, but with H_2_ remaining bound in a higher energy intermediate, and next gets displaced by N_2_. A QM-cluster study^13^ and a combination^64^ of hybrid QM/MM metadynamics simulations and ENDOR measurements have suggested such a mechanism.

It is this initial binding of N_2_ (primarily within the “associative” scenario defined above) that is the focus of our study. We will focus on the uncovering of both the electronic and molecular structure aspects of FeMoco that enable N_2_ binding to occur. In our goal to understand why the **E_4_** state is capable of binding dinitrogen it is equally useful to understand why the earlier **E_0_**, **E_1_**, **E_2_** states are not capable of binding dinitrogen and to understand how both the cofactor redox state as well as single Fe oxidation state and local spin states correlate with binding energies. Diamagnetic substitution of the cofactor with a quasi-restricted orbital (QRO) transformation allows us to gain such insights, and we utilize this technique to shed light on the local ligand field of the individual Fe ions involved in N_2_ binding in each of the redox, spin, and BS states calculated in this work. Finally, we discuss how the protonation state of His195 affects binding energies.

## 2 Computational Details

The QM/MM model in this work builds on a previous model setup from our group^10^ that has successfully been used for several **E_*n*_** states.^10−12,14^ However, our previous studies employed a truncated spherical droplet model for the actual QM/MM modeling. While the spherical droplet QM/MM model is known to be a highly economical yet accurate strategy for QM/MM reaction profile studies on proteins,^65,66^ a recent study from our group on VFe protein of V nitrogenase^67^ demonstrated some sensitivity to the size of the model, suggesting that bulk electrostatics have an effect on the redox properties of the cofactor. In this work, we utilize the full box from the original classical MM setup of MoFe protein^10^ without truncating the system.

A new QM/MM code, ASH,^68^ developed in our group, interfaced to the OpenMM library^69^ and the ORCA quantum chemistry code^70^ was used in this study. ASH has been designed with the aim of having full flexibility to perform QM/MM calculations on large metalloproteins, with an interface to the OpenMM molecular mechanics library and a flexible interface to the ORCA quantum chemistry code with specific functionality to conveniently control BS-state convergence. The CHARMM36 force field^71^ was used to describe the MM region as in previous papers in our group.^10−12,14,67,72−74^ Geometry optimizations were performed using an interface to the geomeTRIC optimization library^200^ using HDLC internal coordinates.^201^ Standard electrostatic embedding QM/MM with link atoms and a charge-shifting scheme was used.^75^ A comparison between ASH and Chemshell^76^ results was performed on the spherical droplet model to demonstrate that previous QM/MM results were reproducible (see Section 17 in the SI). The full solvated model of MoFe protein has 320,814 atoms in the **E_0_** state (see Figure S1a in the SI). An active region of 990 atoms was defined, centered on FeMoco (see Figure S1b in the SI).

Different quantum mechanics regions (QM) were used in this work, labeled QM-I to QM-VI as shown in Figure S1 of the SI. The QM-I region has 56 atoms (**E_0_**-state; link-atoms included) consisting of FeMoco, α-Cys275, α-His442 sidechains and a homocitrate ligand attached to Mo (Figure 1 and Figure S1c). The homocitrate is singly protonated as is generally accepted.^10,77,78^ The QM-V region (Figure S1h) has eight more residues sidechains than the QM-I region: α-Val70, α-Arg96, α-Gln191, α-His195, α-Ser278, α-Glu380, α-Phe381, and α-Arg359. The QM-VI region has 12 more residues (10 H_2_O molecules, α-Gly356, and α-Gly357) than the QM-V region, or ∼187 atoms in total.

In this work, geometry optimizations were first obtained for all tested spin states and BS solutions using the smaller QM-I region and the larger QM-V region was used to perform geometry optimizations on the lowest-energy isomers (according to the QM-I data). Finally, single-point calculations using the large QM-VI region were performed on the QM-V geometries. Section 2 in the SI compares energy differences obtained with different QM regions.

### 2.1 Quantum Chemical Details

The r^2^SCAN^79^ density functional was used in QM and QM/MM calculations using the implementation in the LibXC library.^80^ This functional emerged as the most accurate in a recent benchmarking study from our group on the structural properties of spin-coupled iron–sulfur dimers as well as FeMoco (compared to high-resolution X-ray structures),^74^ giving on average slightly better molecular structures than the TPSSh functional that we have previously favored in our FeMoco research. In particular, the Fe–Fe and Mo–Fe distances of the spin-coupled metal dimers were found to be highly sensitive to the treatment of the covalency of the Fe–S bond, suggesting the r^2^SCAN functional to better treat the electronic structure of complex Fe–S clusters. The r^2^SCAN functional has furthermore been shown to be a highly reliable density functional for both maingroup as well as closed-shell and open-shell transition metal reaction energies (primarily organometallic systems tested) and transition metal spin-crossover energies according to benchmark studies.^81−83^ The r^2^SCAN functional has additionally the practical advantage of not including HF exchange, unlike the TPSSh functional. This results in less costly QM steps, which allowed us to explore more possibilities in this work as well as using large QM-regions while making no sacrifice with respect to overall electronic structure treatment of FeMoco. SCAN,^84^ the predecessor to r^2^SCAN, was known to suffer from numerical problems, which led to the development of r^2^SCAN, which suffers less from numerical problems. Nonetheless, we investigated the numerical grid dependence of the r^2^SCAN exchange–correlation integrals, as shown in the Section 20 of the SI. The “defgrid2” integration grid in ORCA, used in most calculations in this work, was found to give satisfactory numerical precision; we estimate numerical grid uncertainty in reaction energies to be on the order of ∼0.2 kcal/mol.

In this work, we continue to utilize an all-electron scalar relativistic treatment that we have shown to be close to the all-electron relativistic basis set limit of iron–sulfur compounds.^74^ The D4 dispersion correction^82,85^ was used in all calculations. The Split-RI-J approximation^86^ as implemented in ORCA was used to speed up the Coulomb integral evaluations utilizing a decontracted general Coulomb fitting auxiliary basis set^87,88^ (named SARC/J in ORCA). The ZORA scalar relativistic Hamiltonian^89,90^ was used with the relativistically recontracted ZORA-def2-TZVP^87,91^ basis set on Fe, S, carbide, hydrides, and S-bound protons of FeMoco as well as the dinitrogen ligand in N_2_-bound states. The all-electron SARC-ZORA-TZVP^92^ basis set was used for Mo. The smaller ZORA-def2-SVP basis set was used for other atoms (including protein residues). Test calculations indicated that differences in using ZORA-def2-SVP or ZORA-def2-TZVP on the homocitrate were tiny (see Section 2 in the SI). The smaller basis set (ZORA-def2-SVP) on homocitrate was used for optimizations with the QM-I region but was increased to ZORA-def2-TZVP when doing single-point calculations using the QM-VI region. Localized orbital calculations used the Pipek–Mezey localization method^93^ and was used to derive electron configuration diagrams of the most relevant states of FeMoco (shown in the SI). The CPCM solvation model^94^ with a dielectric constant of ε = 4 was used for diamagnetically substituted FeMoco cluster models (Section 3.1) to explore N_2_ binding to single-Fe models. Other parameters of the CPCM solvation model are the default settings in ORCA (refrac: 1.3300; rsolv: 1.3000; surface type: Gaussian vdW; radius for H, C, N, O, S, Fe, and Mo: 1.32, 2.04, 1.86, 1.82, 2.16, 2.40, and 2.40 Å, respectively). The solvation charge scheme was a Gaussian charge scheme as implemented in ORCA.^95^ No additional non-electrostatic term was included.

Hirshfeld population analysis^96^ was used to calculate atom charges and spin populations. Vibrational frequencies were calculated numerically using a partial Hessian approach^97^ (N_2_ and the binding Fe defining the partial Hessian). The calculated vibrational frequencies of N_2_ were not scaled. The stretching vibration of free N_2_ at the r^2^SCAN level is 2432 cm^–1^, which is an overestimate compared to the experimental gas value (2330 cm^–1^). In this study, we focus on the shift (Δ) in N_2_ frequency relative to free N_2_ at the same level. The unbound N_2_ was calculated in vacuum for all QM/MM binding energy calculations, while in CPCM-cluster binding energy calculations, CPCM was included (ε = 4).

Binding energy in this work will be used to refer to the energy of binding N_2_ to the most favorable isomer of each redox state while we will occasionally use the term “single-step N_2_ binding energy” when referring to N_2_ binding to a specific isomer. Finally, we note that reported N_2_ binding energies in this work are based on electronic energies at 0 K: Δ*E*^0K^ (without zero-point vibrational energy, ZPVE). The focus of this study is not to give realistic estimates of free energies of binding of N_2_ but rather to understand the trends in the electronic part of the binding energy. While we expect Δ*E*^0K^ to be a decent approximation to Δ*H*^298K^ (ZPVE being the largest missing contribution), we do not include entropic contributions, which will have considerable contributions to the free energies of binding. Unfortunately, translational and rotational entropic contributions from gas phase statistical mechanics (10–15 kcal/mol in favor of the reactant in an A + B → C reaction) may also overestimate entropy effects; in this work, we will report Δ*E*^0K^ binding energies as this is sufficient to discuss the relevant trends, but it should be kept in mind that free energies of binding will differ.

### 2.2 Diamagnetic Substitution and Ligand-Field Diagrams

Due to the complex delocalized electronic structure of Fe and Mo in FeMoco, understanding the local electronic structure as N_2_ binds to Fe is challenging. Localized orbital analysis has been used in previous QM/MM studies from our group^10,12,14,67,73^ as it allows us to derive approximate electron configurations diagrams of all Fe and Mo ions in each FeMoco state, something not possible by decomposition of the spin density. Unfortunately, localized orbitals prevent us from discussing a ligand-field model for each local Fe ion as localized orbitals have no well-defined energies. Diamagnetic substitution (DS) has in previous studies proved useful to get insights into the local electronic structure of FeMoco.^18,73^ Here, all open-shell metal ions, except a chosen one, are replaced by diamagnetic ions with a similar atom radius.

Diamagnetic substitution was used in this work in two ways:

(i) to study N_2_ binding in a diamagnetically substituted version of the resting state cofactor, varying the Fe oxidation state and spin state of a single Fe ion (Fe6). During optimization, only Fe6 and N_2_ were allowed to move.
(ii) to derive approximate ligand-field diagrams of specific Fe sites of FeMoco intermediates throughout the study by utilizing quasi-restricted orbitals of a diamagnetically substituted model.

In each diamagnetically substituted calculation, Mo^3+^ was substituted with In^3+^ and Fe^3+/2+^ centers were substituted with Ga^3+^. The Ga^3+^ ion nuclear charges were modified to have a value of 30.5 rather than 31 to make the atomic charges of the sulfides closer to that of the full FeMoco in the **E_0_** state as previously used.^73^ Because a diamagnetically substituted model contains only a single open-shell Fe ion, we can derive approximate ligand-field diagrams based on the quasi-restricted orbital transformation of the canonical orbitals. Ligand-field diagrams were obtained in a three-step procedure:

(i) determine the local redox state and spin state of the Fe center of interest according to localized orbital analysis of the full FeMoco using the QM-VI region.
(ii) diamagnetically substitute all other Fe ions except one ion (kept in the redox state and spin state suggested by the first step).
(iii) perform a single-point calculation and derive quasi-restricted orbitals, which were used to make a ligand-field diagram.

A step-by-step example is shown in Section 15 of the SI. The ligand-field diagrams derived in this way are of course approximate due to both (i) the spin-pairing enforced by the quasi-restricted orbital transformation of an unrestricted Kohn–Sham determinant with different orbitals for different spins and (ii) the diamagnetic substitution changing the chemical system. For the case of some high-spin metal ion cases, non-Aufbau configurations are found, which is an artifact of the approach. Future work will consider an ab initio ligand-field approach^98^ instead.

### 2.3 Broken-Symmetry States

Ten classes of BS determinants were originally proposed to describe FeMoco (in its **E_0_** state) assuming *C*_3_ symmetry.^21,22^ If the approximate *C*_3_ symmetry is ignored, however, this results in 35 (= ) BS solutions (flipping all 7 Fe sites). This is now known to be a simplification as the Mo ion is an open-shell ion as well.^18^ However, as the Mo ion preferentially adopts an unusual spin-coupled non-Hund configuration,^18^ it is not necessary to consider Mo as part of the spin-flipping problem. The BS determinants can be described in terms of which Fe ions have predominantly local spin-down vectors (according to spin population analysis of the SCF solution). The most favorable broken-symmetry solutions for the **E_0_** state are of the BS7 class and are here labeled as follows: BS235, BS247, and BS346. While energetically very similar (∼1 kcal/mol), these BS determinants differ electronically in terms of locations of mixed-valence delocalized Fe pairs and localized Fe^2+^ or Fe^3+^ ions.^10^ The localized orbital analysis of these three BS solutions is shown in Figure S5 of the SI. The BS235 solution has Fe2, Fe3, and Fe5 spin-down, which results in mixed-valence delocalized pairs between Fe2 and Fe3 (being both spin-down), between Fe1 and Fe4 (being both spin-up), and between Fe6 and Fe7 (being both spin-up), as shown in Figures S5 and S6. For reduced states of FeMoco, especially when the hydride or N_2_ is bound, the nature of the spin coupling problem changes, apparently due to the stability of intermediate or low-spin local configurations on H/N_2_-binding Fe ions. Thus, the BS147 (of BS10 class) was, e.g., found to be the most stable for **E_4_-SP**.^14^ In this study, we have used four BS solutions (BS235, BS247, BS346, and BS147) in initial explorations of all models of the **E_*n*_** states. Additionally, we present additional calculations on more BS states for different **E_4_** isomers that contain a more complicated electronic structure (including Fe ions with low or intermediate spin states) than the other **E_*n*_** states in Section 11 of the SI.

## 3 Results

N_2_ binding was systematically explored to Fe2 or Fe6 sites in QM/MM calculations of the **E_0_**, **E_1_**, **E_2_**, and **E_4_** states and is discussed in Sections 3.2, 3.3, 3.4, and 3.5, respectively. Section 3.6 shows the results of N_2_ binding to **E_4_** for different protonation states of α-His195 on N_2_ binding to the **E_4_-SP** model. We start our discussion about N_2_ binding to FeMoco, however, by showing the results for a diamagnetically substituted version of FeMoco with Fe6 in multiple oxidation and spin states. In our study, we primarily focused on end-on N_2_ binding to Fe2/Fe6 sites *trans* to the Fe–C bond. As discussed in the SI, other N_2_ binding modes are generally found to be unfavorable. Our study is also focused on N_2_ binding energies (calculated as 0 K electronic energies) and are not intended to be realistic estimates of the free energies of binding at room temperature (entropic effects would shift the binding energies considerably).

### 3.1 N_2_ Binding to Diamagnetically Substituted FeMoco: Fe(III) vs Fe(II) vs Fe(I)

Substituting all Fe and Mo ions for diamagnetic ions (Ga^3+^ and In^3+^) in FeMoco except Fe6 in a cluster model of FeMoco allows us to explore how N_2_ binding to a local Fe ion in the FeMoco coordination environment is affected by local oxidation states and spin states without the complications arising from the complex spin coupling and mixed-valence delocalization present. The diamagnetically substituted QM-cluster model, shown in Figure S1, was calculated utilizing a CPCM continuum solvation model. The following eight possibilities for Fe6 were studied: Fe^3+^ (*S* = 5/2, 3/2, 1/2), Fe^2+^ (*S* = 2, 1, 0), and Fe^1+^ (*S* = 3/2, 1/2). We note that the belt Fe ion positions are essentially equivalent in a diamagnetically substituted model of **E_0_** with no protein environment present.

**Figure 2:**
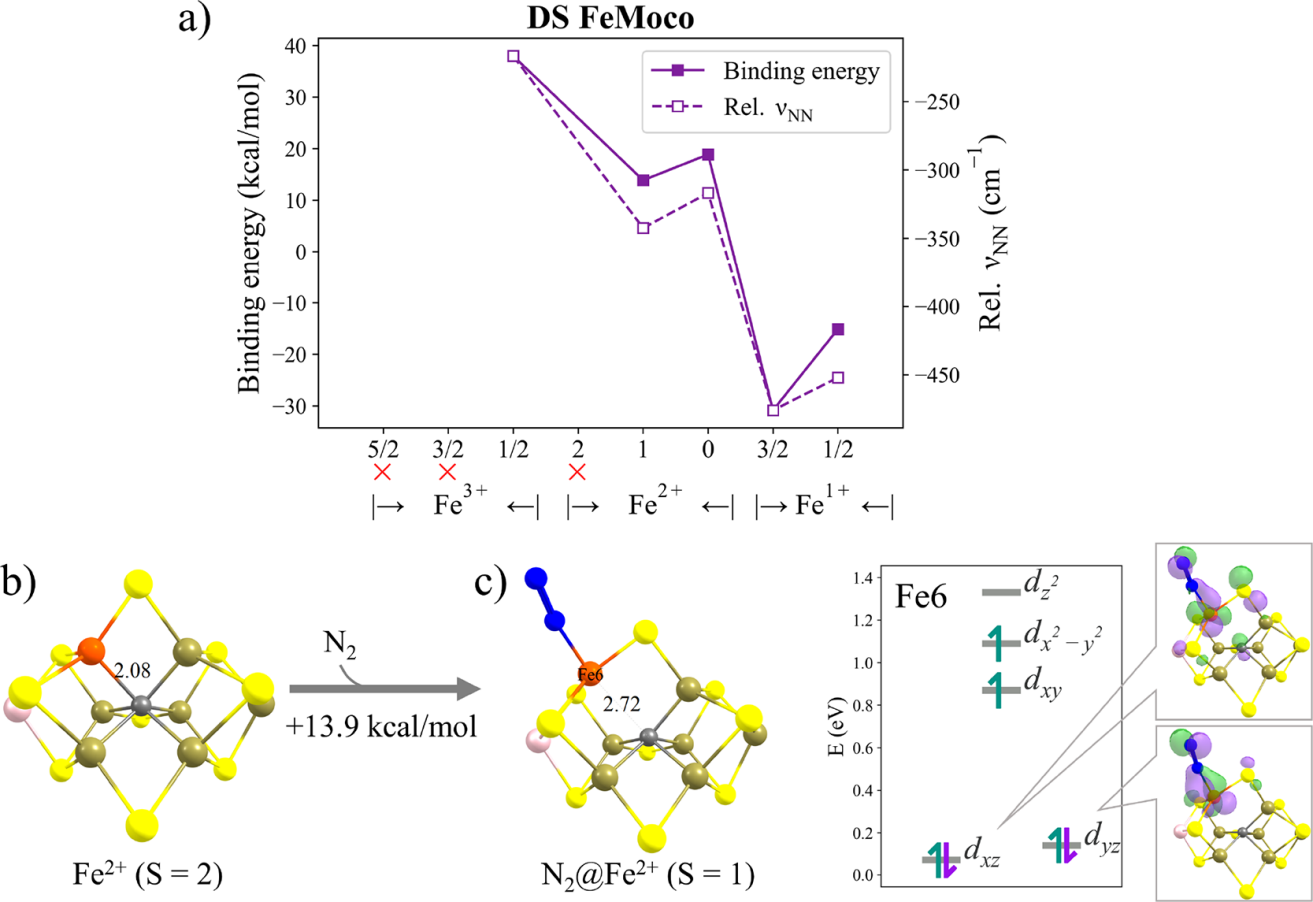
(a) Binding energies and relative ν_NN_ frequencies of the diamagnetically substituted (DS) model in different Fe oxidation states and spin states. Calculated ν_NN_ frequencies are relative to free N_2_ (2432 cm^–1^ at the r^2^SCAN level of theory). Each binding energy is relative to the lowest-energy isomer (always high spin) for each redox state without N_2_. Crosses in red indicate that N_2_ does not bind to Fe of that state. (b) Lowest-energy structure of DS FeMoco with a high-spin Fe^2+^ at Fe6 before N_2_ binding. (c) Optimized structure of N_2_ bound to DS FeMoco with a Fe^2+^ (*S* = 1) and the associated ligand-field diagram of the Fe^2+^ based on quasi-restricted orbitals. Also shown are *d_xz_* and *d_yz_* quasi-restricted orbitals. The full cluster model is shown in Figure S1 of the SI.

Prior to N_2_ binding, the lowest-energy isomer for each redox state has the Fe ion in a high-spin state. Dinitrogen was found to bind to five of the eight states calculated: Fe^3+^ (*S* = 1/2), Fe^2+^ (*S* = 1, 0), and Fe^1+^ (*S* = 3/2, 1/2). N_2_ otherwise spontaneously dissociated for the other cases. The low spin states found, imply that N_2_-bound states of FeMoco will favor a change in spin state upon binding. The QRO ligand-field diagrams of these five states (see Figure 2c and Figure S3 in the SI) reveal the common theme of *d_xz_* and *d_yz_* orbitals being doubly occupied (where the *z*-axis is defined along the Fe–N_2_ bond). The *d_xz_* and *d_yz_* orbitals have the correct symmetry to overlap with the π* orbitals of dinitrogen, and considerable N_2_ π* orbital character can in fact be seen in the QRO orbitals (see Figure 2c for the Fe(II) *S* = 1 case). Further spin-pairing by double occupation of the *d_xy_* orbital as in a Fe^2+^*S* = 0 state offers no additional advantage, in fact the binding energy becomes less favorable (see Figure 2a).

As N_2_ binds to the cofactor, an approximate trigonal bipyramidal geometry might be expected; however, such a geometry is in fact not stable as the Fe–C distance is strongly elongated from 2.08 to 2.72 Å (see Figure 2), suggesting a completely broken Fe–C bond. This Fe–C “bond cleavage” occurred for all five N_2_-bound isomers (see Figure S3). Clearly, this suggests that the coordination change of the binding site Fe is likely to accompany N_2_ binding and we note that a flexible Fe–C interaction has indeed been suggested as a role for the interstitial carbide, with model compounds from Peters being particularly relevant in this context.^99^ However, recent ^13^C ENDOR data of various states of FeMoco are more indicative of a stabilizing rather than flexible role for the interstitial carbide.^100^ In the diamagnetically substituted model, only the Fe and N_2_ atoms were allowed to move and with no protons present either, and elongation of the Fe–C bond constitutes the only structural rearrangement possible.

The N_2_ binding energy becomes more favorable in going from the Fe^3+^ state, via Fe^2+^, to the Fe^1+^ state. While for the Fe^2+^ state, the N_2_ binding is still endothermic by +13.9 to +18.9 kcal/mol, for the Fe^1+^ state, the N_2_ binding has become convincingly exothermic by −15.1 to −30.8 kcal/mol. A more reduced Fe obviously makes it easier to generate double occupations of *d_xz_* and *d_yz_* orbitals (that overlap with N_2_ π*), and a more reduced Fe can even more effectively push electron density into the N_2_ π* orbitals as the Fe d-orbital energies should be shifted to higher energy (closer to N_2_ π* levels). The vibrational frequency of the N–N bond (a well-known indicator of N_2_ bond activation^101^) relative to free N_2_, ν_NN_, correlates well with the trend in binding energies. ν_NN_ decreases from −217 to −476 cm^–1^ as the Fe oxidation state goes from Fe^3+^ to Fe^1+^, which indicates unsurprisingly that a more reduced Fe is positively correlated with the activation degree of N_2_.

Overall, the results using the diamagnetically substituted FeMoco model demonstrate that double occupations of *d_xz_* and *d_yz_* orbitals, a reduced Fe, and the coordination change of the Fe binding site are relevant to favorable terminal N_2_ binding. Such local spin-state changes of the multi-Fe spin-coupled FeMoco seem likely to occur as N_2_ binds to the cofactor; however, structural changes (including those associated with protonation) are likely to play an equally important role, which are not realistically captured in this simple model.

### 3.2 N_2_ Binding to FeMoco: The **E_0_** Redox State

The **E_0_** state with a spin state of *S* = 3/2^31,33^ is the resting state described in the Lowe–Thorneley cycle (Figure 1) and is the best understood redox state of FeMoco. Experiments have revealed that FeMoco does not bind N_2_ at **E_0_**.^7,41−43^ The lowest energy isomer is found to be **E_0_-BS235** in the *M*_S_ = 3/2 spin state (this differs slightly from our original TPSSh-QM/MM study^10^ where **E_0_-BS346** was more stable). As discussed in previous studies, the **E_0_-BS235**, **E_0_-BS346**, and **E_0_-BS247** are generally found to be very close in energy.^10,102^ While the electronic structures of these three BS solutions are related, the three different BS solutions change the location of the minority-spin electrons and hence the mixed-valence delocalized Fe(2.5)-Fe(2.5) pairs. This means that the local electronic structure of the Fe6 (or Fe2) position is dependent on which BS solution is calculated. For the BS235 and BS247 solutions, the Fe6 ion is involved in a mixed-valent delocalized Fe(2.5)-Fe(2.5) pair with Fe7 or Fe5, respectively, while for BS346, Fe6 would be ferric (Fe^3+^). Figure S5 in the SI shows the electronic structure diagrams of all three BS solutions based on the localized orbital analysis.

**Figure 3:**
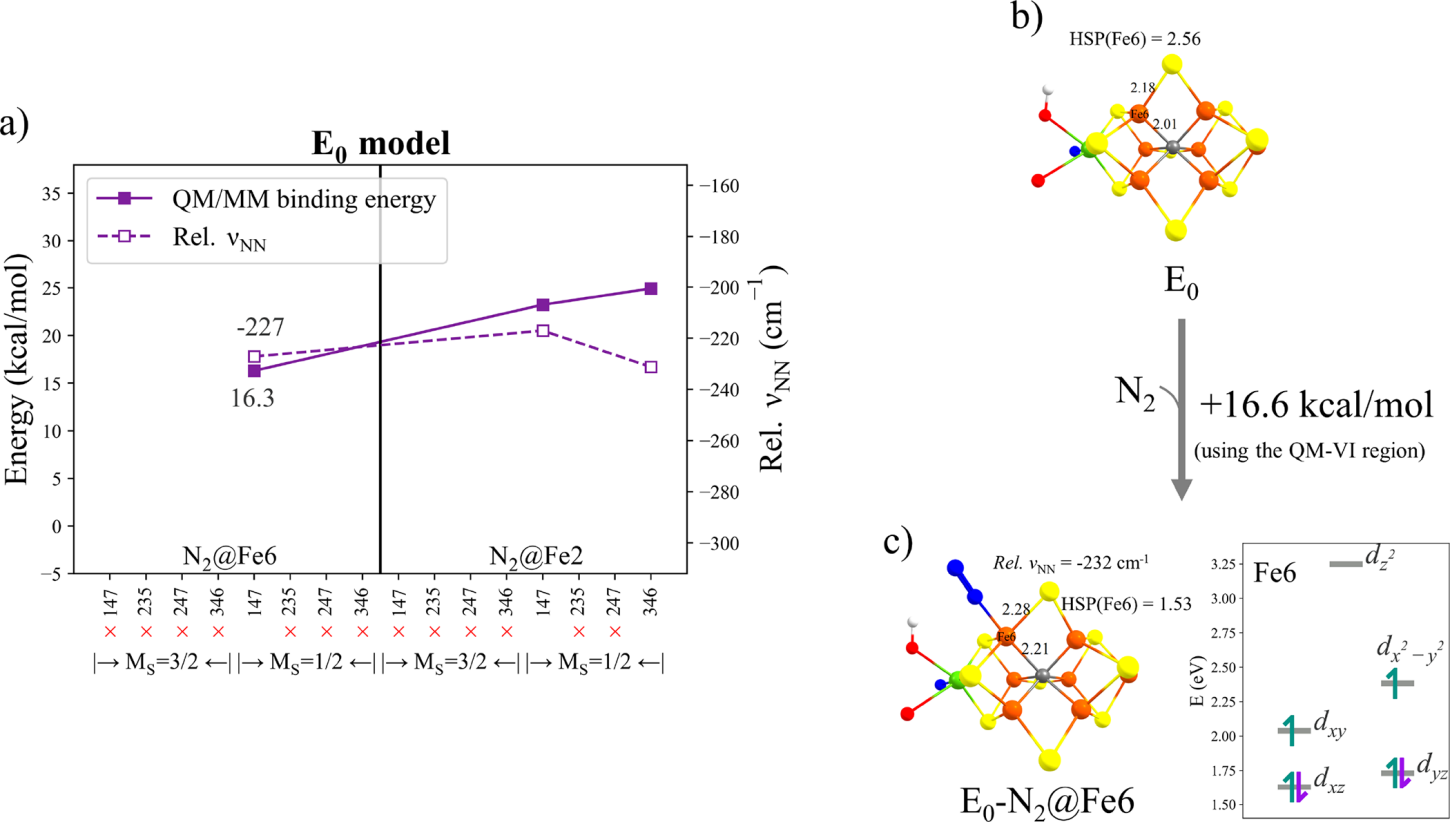
(a) QM/MM N_2_ binding energies and relative ν_NN_ frequencies (relative to free N_2_: 2432 cm^–1^) of the **E_0_** state of FeMoco (using the QM-I region). The *x*-axis labels indicate the BS solution and *M*_S_ spin state of the N_2_-bound state (e.g., “147” is the shorthand name of BS147, Fe1, Fe4, and Fe7 being spin-down). Red crosses indicate no stable N_2_-bound minimum found. (b) Structure of **E_0_-BS235** with *M*_S_ = 3/2. (c) Structure of **E_0_-BS147** (*M*_S_ = 1/2) with N_2_ bound to Fe6 and the ligand-field diagram of Fe6. The N_2_ binding energy is relative to the **E_0_-BS235** in the *M*_S_ = 3/2 spin state in all cases. HSP(atom) indicates the Hirshfeld spin population of that atom.

Sixteen N_2_-bound states were studied by binding N_2_ to Fe2 or Fe6 in the **E_0_** state in two spin states (*M*_S_ = 3/2, 1/2) and four BS solutions using the QM-I region, as shown in Figure 3, but only three of these states were found to give N_2_-bound minima: **E_0_-N_2_@Fe6-BS147**, **E_0_-N_2_@Fe2-BS147**, and **E_0_-N_2_@Fe2-BS346**, all of which are in *M*_S_ = 1/2 spin state. The N_2_ binding energies to Fe6 and Fe2 are found to be 16.6 and 21.4 kcal/mol, respectively, using the larger QM-VI region and the BS147 solution. The difference in N_2_ binding energy between Fe2 and Fe6 sites appears to be due to the steric clashing of the N_2_ ligand with the His195 residue when bound to Fe2. This preference is reversed when a cofactor-only cluster-continuum model is used, as shown in Figure S7 in the SI.

Localized orbital analysis (see, e.g., Figure S29) and Hirshfeld spin populations are consistent with local spin state changes occurring for most of the N_2_-bound states. For the **N_2_@Fe6** state highlighted in Figure 3c, the low Hirshfeld spin population of 1.53 on Fe6 is highly suggestive of a local spin state change and localized orbital analysis confirms spin-pairing occurring for *d_xz_* and *d_yz_* orbitals, as seen in Figure S29 of the SI. The QRO-based ligand-field diagram of the Fe6 ion (using diamagnetic substitution and choosing Fe^2+^*S* = 1 based on localized orbital analysis) reveals a changing ligand field where the *d*_*z*^2^_ orbital is destabilized due to the approximate trigonal bipyramidal geometry in the N_2_-bound state.

As shown in the FeMoco structures before and after N_2_ binding to Fe6, the Fe6-C and Fe6-S2B distances become longer after N_2_ binding (Figure 3), although the Fe6-C elongation is far from the complete cleavage that was seen for the diamagnetically substituted model in Section 3.1. Clearly, however, this implies that N_2_ binding to Fe will lead to structural changes that are likely to depend on the protonation state of the cofactor.

As higher **E_*n*_** states of the cofactor are known to involve protonated belt sulfides (the S2B sulfide bridging Fe2 and Fe6 in particular), we also carried out **E_0_** calculations with an additional H^+^ present on S2B, labeled **E_0_H^+^** here (but without an electron as in the **E_1_** state). We note in this context that Rees and co-workers have suggested based on X-ray structures (1.85 and 2.30 Å resolution) of MoFe proteins (both *Azotobacter vinelandii* and *Clostridium pasteurianum*) under pH = 4.5–6 conditions, that an **E_0_H^+^** state may rather be protonated at either S5A or S3A.^103^ As our study is restricted to studying N_2_ binding to Fe2 and Fe6, we only study the S2B-protonated state here.

**Figure 4:**
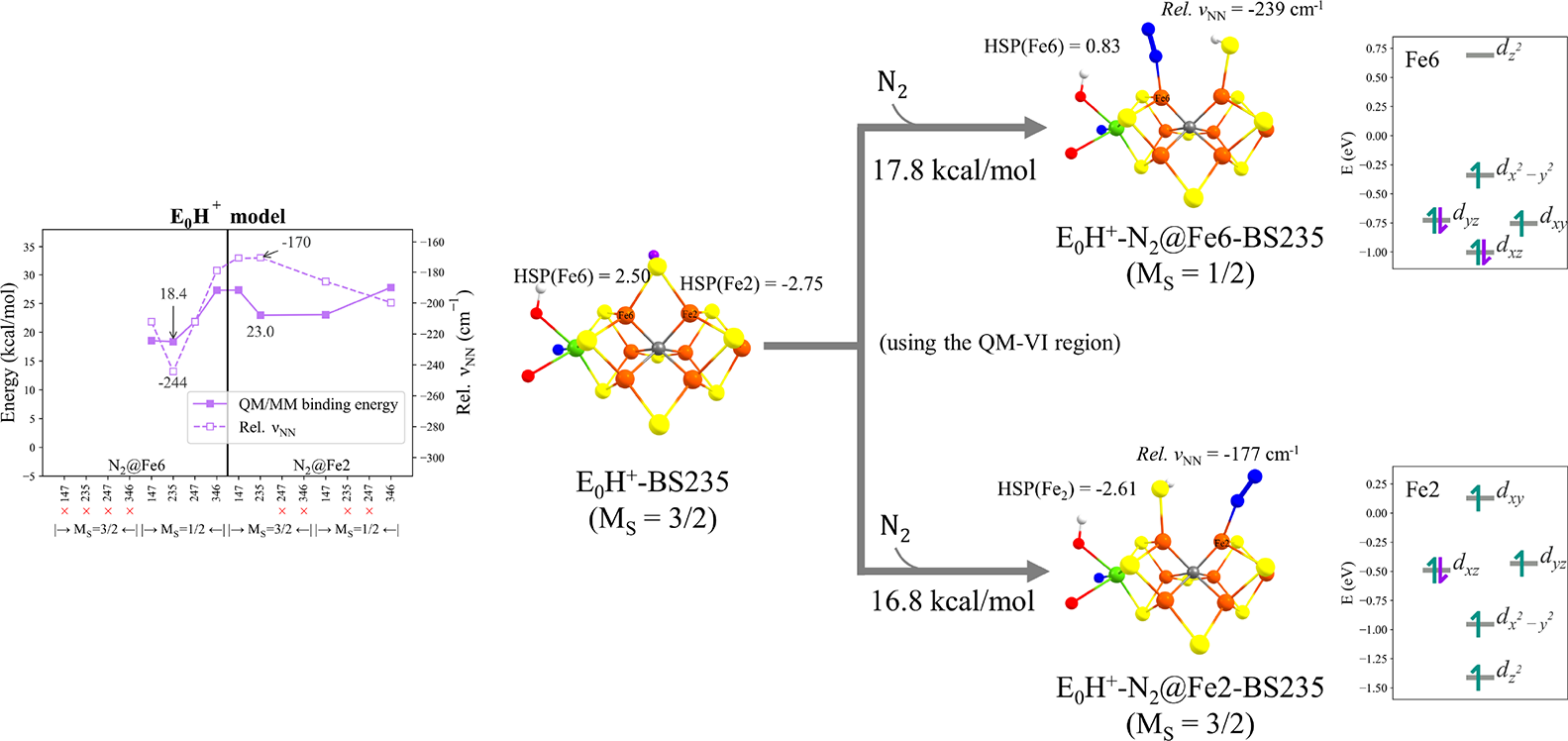
The left plot shows N_2_ binding energies and relative N–N stretching frequencies (relative to free N_2_: 2432 cm^–1^) for **E_0_H^+^** calculated using the QM-I region. Geometries and N_2_ binding energies for the most favorable N_2_-bound **E_0_H^+^** models using the QM-VI region are shown on the right. All N_2_ binding energies are relative to the lowest-energy isomer: **E_0_H^+^-BS235** with *M*_S_ = 3/2. HSP(atom) indicates the Hirshfeld spin population of that atom.

Figure 4 shows the binding of N_2_ to the FeMoco **E_0_H^+^** model. The results reveal that the availability of an additional proton on S2B introduces additional flexibility for N_2_ binding, the proton enabling spontaneous full belt sulfide-bridge opening to give a partially or even fully terminal sulfhydryl group, and additional favorable N_2_-bound states could be found for both **N_2_@Fe2** and **N_2_@Fe6** (compared to regular **E_0_**). This hemilability of protonated belt sulfur bridges in reduced **E_*n*_** states has been found to be a common structural theme in previous reduced and ligand-bound studies by us^12,14,73^ but had not previously been explored at the **E_0_** redox level. However, as for the **E_0_** state, the N_2_ binding energy of N_2_ remains endothermic and high (>16.8 kcal/mol), suggesting structural rearrangement alone via sulfur protonation not to be sufficient for favorable N_2_ binding to FeMoco. This lack of N_2_ binding to the resting state is of course fully consistent with experimental observations. A local spin-state change on Fe6 was found for the **E_0_H^+^-N_2_@Fe6** state but not for the **E_0_H^+^-N_2_@Fe2** state (see Figure 4).

### 3.3 N_2_ Binding to FeMoco: The **E_1_** Redox State

While there is no indication of the **E_1_** state binding N_2_, this state has been implicated in binding alternative substrates and inhibitors such as CO and acetylene,^5,73^ indicating that it possesses increased reactivity compared to the resting state. Previous computational studies^29,30^ and a recent joint EXAFS-QM/MM study^11^ suggest the state to be best described as an Fe-reduced cofactor with a protonated S2B belt sulfide bridge. The spin state is known to be integer spin,^24^ and an *M*_S_ = 2 broken-symmetry state was the most favorable according to the QM/MM calculations.^11^ The added electron was found to localize in the MoFe_3_ subcubane, on either Fe5, Fe6, or Fe7, depending on whether the BS235, BS346, or BS247, respectively, were calculated, with the spin-down Fe in the MoFe_3_ sub-cubane, thus determining the site of the redox event (Fe^3+^ → Fe^2+^).

**Figure 5:**
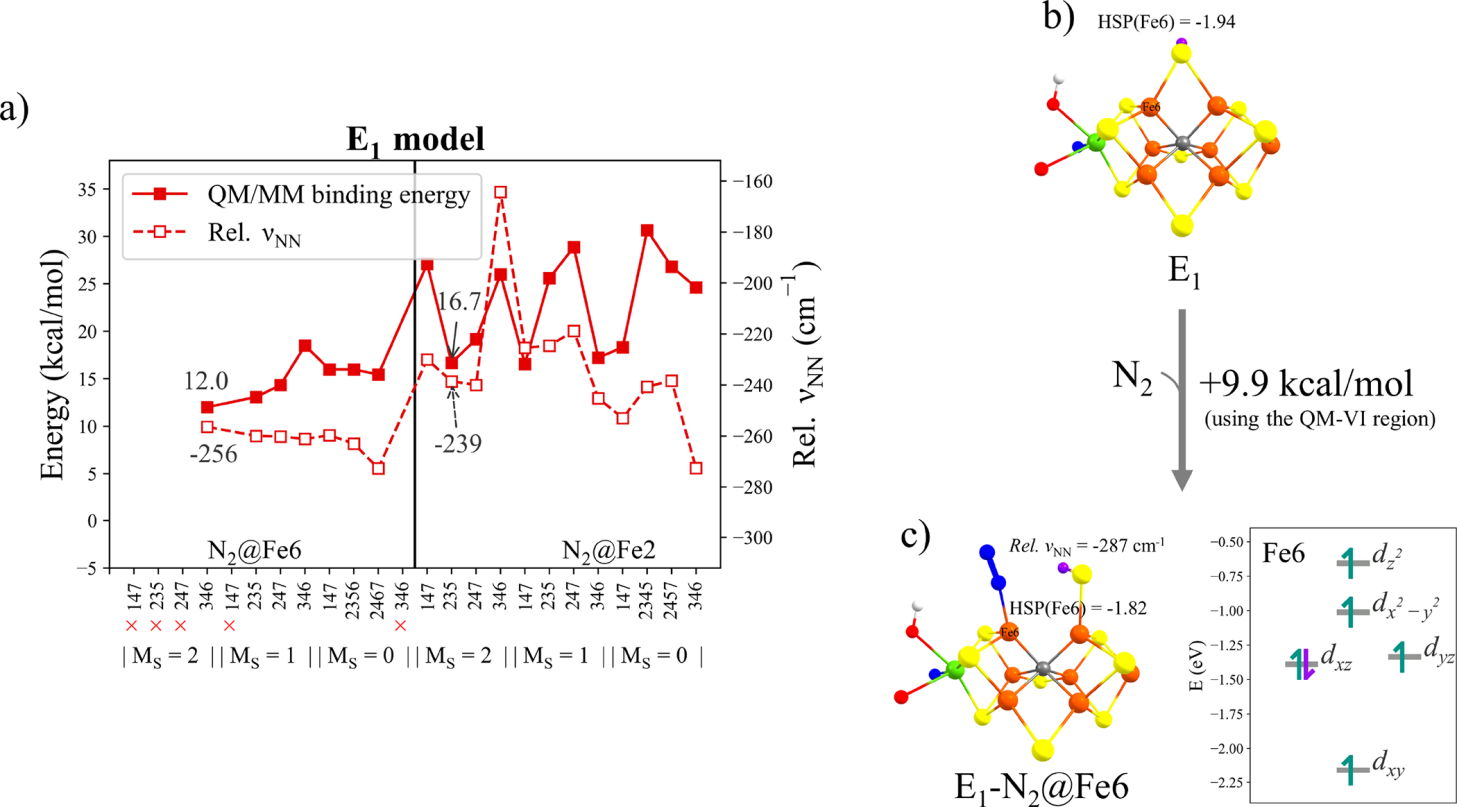
(a) N_2_ binding energies and relative ν_NN_ frequencies for **E_1_** models (QM-I region calculations). (b) Structure of **E_1_-BS346** with *M*_S_ = 2. (c) Structure of **E_1_-BS346** with N_2_ bound to Fe6 in the *M*_S_ = 2 state and relevant QRO-ligand-field diagram of Fe6. The localized orbital analysis shown in Figure S9 of the SI suggests that Fe6 is best described as a high-spin Fe^2+^ ion. The results shown in (b) and (c) were calculated using the QM-VI region. The N_2_ binding energies are relative to the **E_1_-BS346** with *M*_S_ = 2 (b) when using both QM-I and QM-VI regions.

Twenty-four isomers were systematically explored with N_2_ bound to Fe2 or Fe6 in three spin states (*M*_S_ = 0, 1, 2) and four BS solutions. As shown in Figure 5, considerably more states were found to give N_2_-bound local minima at the **E_1_** redox level compared to the **E_0_** redox level. As found for **E_0_H^+^**, all N_2_-bound states resulted in the protonated belt-sulfide (S2B) becoming terminal on the other Fe as N_2_ bound to Fe2 or Fe6_._ The hemilability of the belt-sulfide thus acts to preserve the (distorted) tetrahedral coordination of both Fe ions as N_2_ binds.

Localized orbital analysis was performed for all N_2_-bound states, which showed that most states showed spin pairing of *d_xz_* and *d_yz_* orbitals at the N_2_-binding Fe site, with some showing spin-pairing of only one of these orbitals. For the most favorable N_2_-bound state, **E_1_-N_2_@Fe6**, shown in Figure 5c, however, only the *d_xz_* orbital on Fe6 showed spin-pairing, as did the state without N_2_ according to similar Hirshfeld spin populations of Fe6 (see ligand-field diagrams in Figure 5c). The slightly more favorable N_2_ binding energies in the **E_1_** state for **E_1_-N_2_@Fe6** and **E_1_-N_2_@Fe2** states of 9.9 and 11.2 kcal/mol, respectively, compared to the **E_0_**/**E_0_H^+^** states are likely to be primarily due to the overall more reduced Fe environment of the cofactor. This also results in increased activation of the N_2_ ligand with a ν_NN_ = −287 cm^–1^ shift of the N–N stretching frequency compared to free N_2_. The N_2_ binding energy is still found to be rather endothermic, which is fully consistent with the lack of experimental evidence for N_2_ binding in the **E_1_** state. This result can be contrasted with the more favorable calculated binding energy for CO in this same redox state, which was found to be −8.3 kcal/mol (TPSSh-QM/MM level of theory) in our previous study.^73^ CO binding to Fe6 of the **E_1_** state using the same QM level as N_2_ binding to FeMoco (r^2^SCAN and the QM-I region) was also studied, shown in Section 7 of the SI, indicating a similar binding energy (−9.6 kcal/mol) as our previous study. Thus, despite an almost identical coordination geometry of the **E_1_-CO@Fe6** species and the **E_1_-N_2_@Fe6** species, favorable binding of N_2_ to FeMoco clearly requires some missing element.

### 3.4 N_2_ Binding to FeMoco: The **E_2_** Redox State

The **E_2_** redox state has been proposed to contain a hydride based on the state’s ability to evolve H_2_ (with slow kinetics as recently revealed^9^) as well as its photochemical properties.^34^ The **E_2_** state thus would appear to share a hydride in common with the N_2_-binding **E_4_** state; however, in contrast, the **E_2_** state has not been found to bind N_2_.

**Figure 6:**
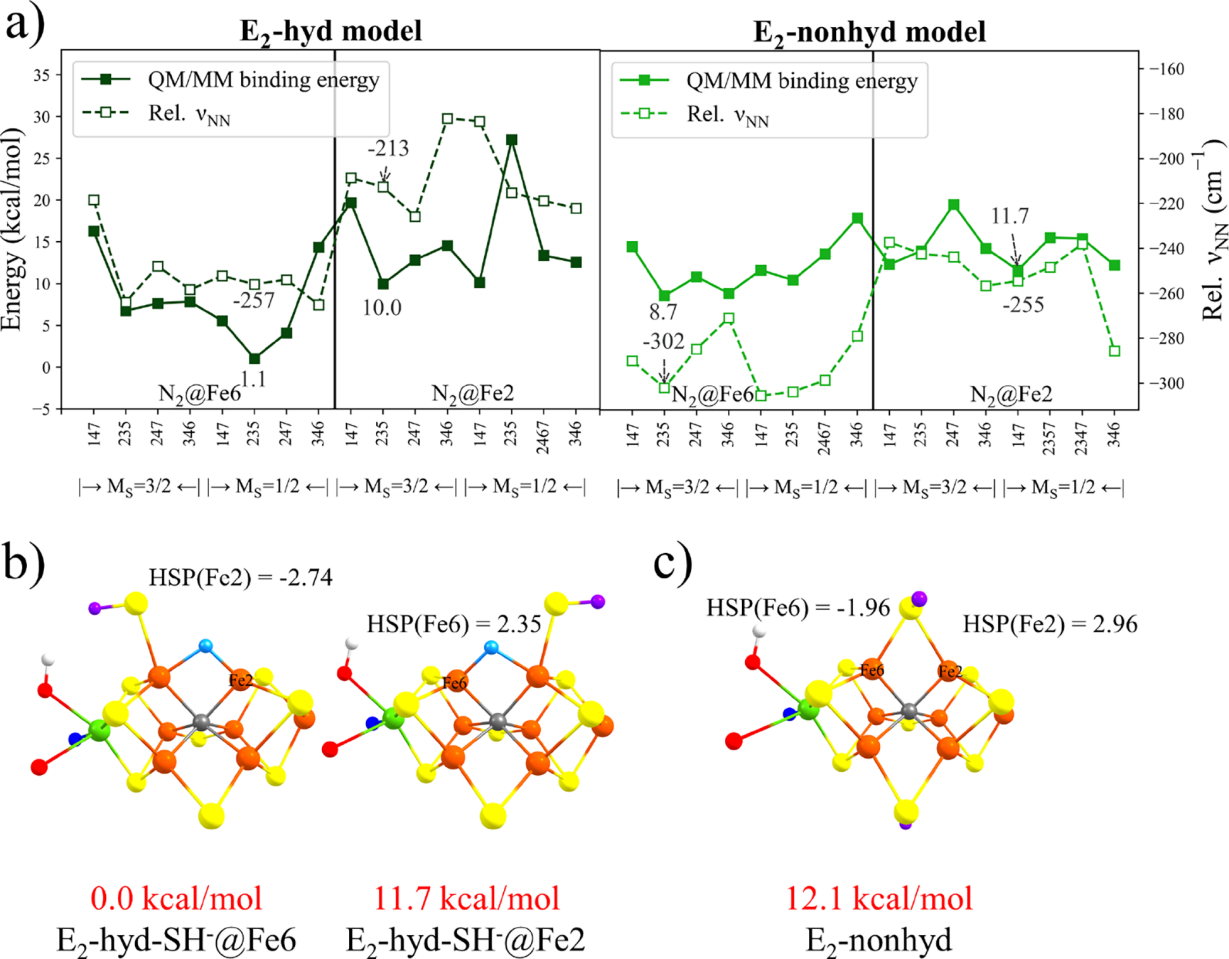
(a) N_2_ binding energies and relative N–N frequencies (QM-I region data) for **E_2_-hyd-SH^–^@Fe6**, **E_2_-hyd-SH^–^@Fe2**, and **E_2_-nonhyd**. The N_2_ binding energies of **E_2_-hyd** and **E_2_-nonhyd** are relative to the left structure of (b) and (c) using the QM-I region, respectively. Relative energies if isomers in (b) and (c) used the QM-VI region.

Unlike the **E_1_** state, where computational studies and most experimental studies are in good agreement on the most likely model, the **E_2_** state offers more structural possibilities. In a recent QM/MM study,^12^ we performed a detailed exploration of the energy landscape of the **E_2_** redox state using a TPSSh-QM/MM protocol. Two isomers were found to be the most energetically favorable: (i) a structure with a hydride bridging Fe2 and Fe6 with a terminal sulfhydryl group on Fe6, **E_2_-hyd-SH^–^@Fe6** (see Figure 6b) and (ii) a nonhydride, doubly belt-sulfur protonated state, **E_2_-nonhyd** (see Figure 6c). The hemilability of the protonated belt sulfur bridge (S2B) allows it to become a terminal sulfhydryl group that stabilizes a bridging hydride between Fe2 and Fe6. Additionally, we included an alternative **E_2_** isomer, **E_2_-SH^–^@Fe2** (see Figure 6b). This isomer is predicted to be relatively high in energy (11.7 kcal/mol using the QM-VI region) compared to **E_2_-SH^–^@Fe6**, likely due to steric repulsion involving the terminal SH^–^ group and His195. This makes it an unlikely candidate for the **E_2_** state (previous results^12,30,35,40^ also found a similar trend); however, as the results for **E_0_** and **E_1_** have revealed, stronger binding is found to Fe6 than Fe2, making N_2_ binding to such a geometry with an open coordination site to Fe6 of interest. These three types of **E_2_** isomers were thus tested for their ability to bind dinitrogen.

**Figure 7:**
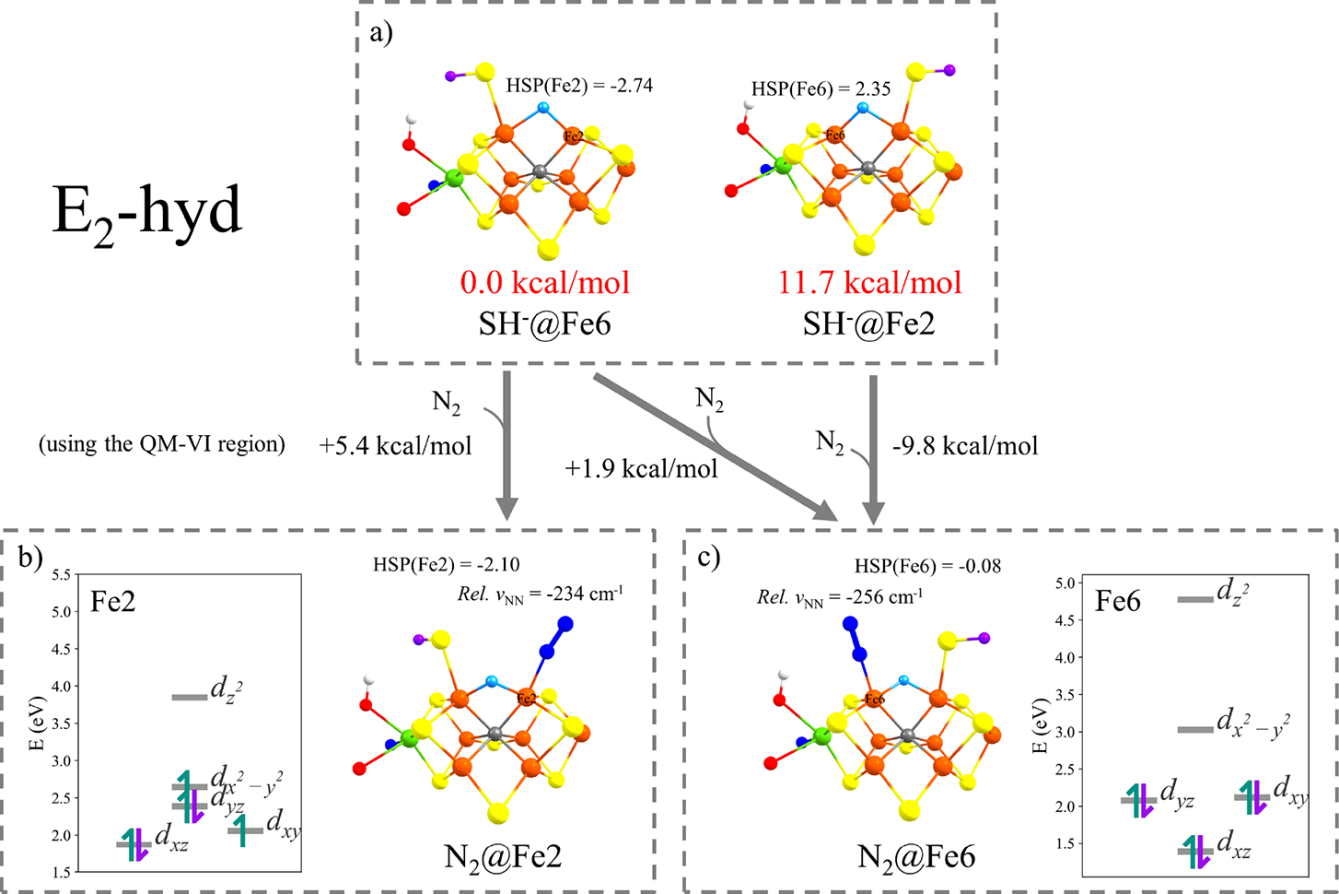
**E_2_-hyd** isomers shown in (a) (*M*_S_ = 3/2 BS235 states) and (b and c) N_2_-bound versions as well binding energies. Ligand-field diagrams of the indicated Fe ion are shown for Fe2 and Fe6 after N_2_ binding. The N_2_-bound structures, **E_2_-hyd-N_2_@Fe2-BS235** and **E_2_-hyd-N_2_@Fe6-BS235**, have *M*_S_ = 3/2 and *M*_S_ = 1/2 spin states, respectively. The energies here differ from Figure 6a as they use different QM regions (here QM-VI).

Figure 6 shows N_2_ binding energies and relative N–N frequencies for **E_2_-hyd** and **E_2_-nonhyd** isomers calculated using the QM-I region. We note that binding energy refers here to the energy of binding N_2_ to the lowest-energy isomer of either the **E_2_-hyd** state or the **E_2_-nonhyd** state. The results reveal a considerably changed picture with respect to N_2_ binding compared to the **E_0_**/**E_1_** data. There is now a much stronger tendency for binding N_2_ according to the **E_2_-hyd** isomer data. As shown more clearly in Figure 7, binding N_2_ to **E_2_-hyd-SH^–^@Fe6** (the most favorable **E_2_** isomer) at the Fe2 site is uphill by +5.4 kcal/mol (a considerable shift compared to **E_0_**/**E_1_**). Even more favorable is to bind N_2_ directly to the alternative isomer **E_2_-hyd-SH^–^@Fe2**, giving a single-step exothermic binding energy of −9.8 kcal/mol, implying a starkly different affinity for binding N_2_. Calculating the binding energy relative to the more favorable **E_2_** isomer (**E_2_-hyd-SH^–^@Fe6**) reduces this binding energy down to +1.9 kcal/mol, which still implies that **E_2_-hyd** isomers behave quite differently to **E_0_** and **E_1_** with respect to N_2_ binding.

In sharp contrast to **E_2_-hyd** models, the data for the **E_2_-nonhyd** models in Figure 6 might appear at first glance to be of little interest. The binding energies are more endothermic, closer to the **E_1_** data in magnitude: being +8.5 (**E_2_-nonhyd-N_2_@Fe6**) and +6.2 (**E_2_-nonhyd-N_2_@Fe2**) kcal/mol using the largest QM-VI region (see Figure S11). However, the endothermic binding energies of **E_2_-nonhyd** models are contrasted with the relative N–N frequencies that are now larger in magnitude than seen before, especially for the Fe6 site, and curiously do not correlate well with binding energies. These **E_2_-nonhyd-N_2_@Fe6** states clearly activate N_2_ more than other FeMoco states, yet the binding energies are unfavorable. A possible interpretation of this conundrum is that these states are favorable with respect to the Fe-N_2_ interaction but N_2_ binding leads to such an unfavorable electronic structure for the rest of the cofactor that the binding energy remains unfavorable.

We note that in our previous study, the total QM/MM energy difference between **E_2_-hyd** and **E_2_-nonhyd** was reported to be 0.1 kcal/mol.^12^ The energy difference in our present study is predicted to be much larger: 12.1 kcal/mol (QM-VI region). As discussed in the SI, the difference arises primarily due to different DFT methods used (r^2^SCAN vs TPSSh) and also due to a slightly different QM/MM setup. A recent QM/MM study using different functionals (TPSS, r^2^SCAN, TPSSh, and B3LYP) but small basis set (def2-SV(P)) also reports a similar energy difference.^35^ Such a large difference between DFT methods that actually predict very similar FeMoco geometries (and energies of **E_4_** isomers as shown in the SI) appears at first troubling. It should be pointed out, however, that it is not too surprising that the energy difference between two states with electrons either localized in Fe-hydrides vs in Fe d-orbitals for this complicated multimetal cofactor would be challenging and highly sensitive to the DFT method. At this point, the relevance of **E_2_-nonhyd** as a model for the **E_2_** state is thus unclear, being so sensitive to the functional. The possible relevance of a N_2_-bound **E_2_-nonhyd** state in the overall mechanism will be discussed later.

The more favorable N_2_ binding energies (although still endothermic) for the **E_2_-hyd** FeMoco states would seem to be connected to the presence of a hydride bound to the N_2_-binding Fe ion (Fe2 or Fe6). The presence of a hydride ligand in the **E_2_-hyd-N_2_@Fe2** or **E_2_-hyd-N_2_@Fe6** leads to unique distorted trigonal bipyramidal geometries. Such a ligand-field is expected to lead to destabilization of the *d*_*z*^2^_ orbital, and this can be seen in the QRO-based ligand-field diagrams for these two states in Figure 7b,c. The raised *d*_*z*^2^_ orbital level would then no longer be populated, and this appears to lead to the stabilization of intermediate-spin Fe(II) for **E_2_-hyd-N_2_@Fe2** and low-spin Fe(II) for **E_2_-hyd-N_2_@Fe6** according to localized orbital analysis. The Hirshfeld spin populations of Fe2 and Fe6 when N_2_ is bound are 2.10 and 0.08 in agreement with the localized orbital analysis. These two lower spin states arise due to spin-pairing or double occupation of the *d_xz_* and *d_yz_* orbitals that, as mentioned, are capable of π* backbonding to the N_2_ ligand. The changed ligand-field in these states due to the presence of the hydride likely makes these electron configurations more favorable than the otherwise weak-field tetrahedral ligand-field that favors high-spin states.

### 3.5 N_2_ Binding to FeMoco: The **E_4_** Redox State

The **E_4_** state gives rise to an *S* = 1/2 EPR signal.^36,37^^1^H ENDOR experiments^38^ have revealed that the state contains two Fe-hydrides and two S-based protons with the hyperfine tensors consistent with bridging rather than terminal hydrides. The precise nature of the **E_4_** geometry with respect to the location of the hydrides and protons is controversial. A model^13^ with all hydrides and protons on the same Fe2-Fe3-Fe6-Fe7 face, suggested by Raugei and Hoffman, was put forward as being the most consistent with the hyperfine tensor orientations, while Cao and Ryde reported that such a model but with two protons pointing to opposite directions has slightly lower energy,^15^ here labeled **E_4_-DP-Fe2/6(5)** (DP-Fe2/6(5): hydrides on different pairs of Fe ions and the hydride bridging Fe2 and Fe6 pointing to S5A). Cao and Ryde also found another energetically more favorable model^15^ than **E_4_-DP-Fe2/6(5)**, here labeled **E_4_-DP-Fe2/6(3)** (DP-Fe2/6(3): hydrides on different pairs of Fe ions and the hydride bridging Fe2 and Fe6 pointing to S3A), which appeared also to be consistent with the ENDOR data. In a study^14^ from our group, we put forward different models, here labeled **E_4_-SP**, that consist of a terminal sulfhydryl group on either Fe2 or Fe6 and bridging hydrides between Fe2 and Fe6. These **E_4_-SP** models were energetically favored over other models tested, apparently due to the energetic stabilization associated with opening the protonated S2B belt sulfide and stabilizing bridging hydrides between Fe2 and Fe6. As discussed in the previous study, there are multiple **E_4_** isomers of this type, i.e., with an open belt sulfide bridge, but with hydrides either bridging, partially bridging, terminal, or even with an H_2_ adduct. The most favorable **E_4_** model found in this work is **E_4_-SP-SH^–^@Fe6** (*M*_S_ = 3/2) shown in Figure 9c. While this model is predicted to have the wrong spin state (and features one bridging and one terminal hydride), it is only 1.7 kcal/mol lower in energy than the next-lowest energy model **E_4_-SP-SH^–^@Fe6** (*M*_S_ = 1/2) model, shown in Figure 9d, which is the same model discussed in our previous work^14^ (labeled **E_4_-o** in that study). The **E_4_-SP-SH^–^@Fe6** (*M*_S_ = 1/2) and **E_4_-SP-SH^–^@Fe2** (*M*_S_ = 1/2) isomers are the ones of primary interest, as they feature bridging hydrides (and thus in qualitative agreement with the ENDOR data) but also because they feature a more accessible N_2_ binding site on either Fe2 or Fe6, respectively.

In this study, we do not seek to come to a clear conclusion regarding the nature of the **E_4_** state. In the SI, we present additional energetic data that compares the energies of the **E_4_-DP-Fe2/6(3)**, **E_4_-DP-Fe2/6(5)**, and the **E_4_-SP** models with four different density functionals (r^2^SCAN, TPSSh,^104,105^ B97-D3,^90,106,107^ and B3LYP*^108,109^), as shown in Figure S17, which were the four best performing DFT methods in a recent structural benchmarking study^74^ of iron–sulfur dimers and FeMoco **E_0_** from our group. The results reveal that the four functionals show an overall impressive consistency and they all predict the strong preference of **E_4_-SP** models over **E_4_-DP-Fe2/6(3)** and **E_4_-DP-Fe2/6(5)** models by 15–20 kcal/mol. However, there is clearly a disconnect between the interpretation of the ENDOR data and the calculated energetics that is not easily resolved and additional experiments are likely required for a firm conclusion on the nature of the **E_4_** state.

**Figure 8:**
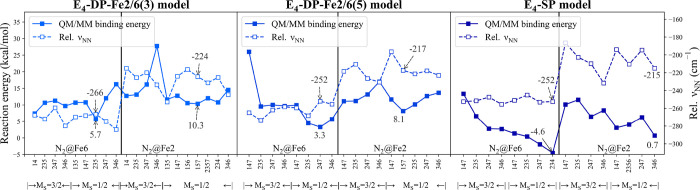
N_2_ binding energies and relative N–N frequencies (relative to free N_2_, ν_NN,free_ = 2432 cm^–1^) for three types of **E_4_** models calculated using the QM-I region. All binding energies are relative to the lowest-energy **E_4_** isomer **E_4_-SP-SH^–^@Fe6** with *M*_S_ = 3/2 (Figure 9c).

**Figure 9:**
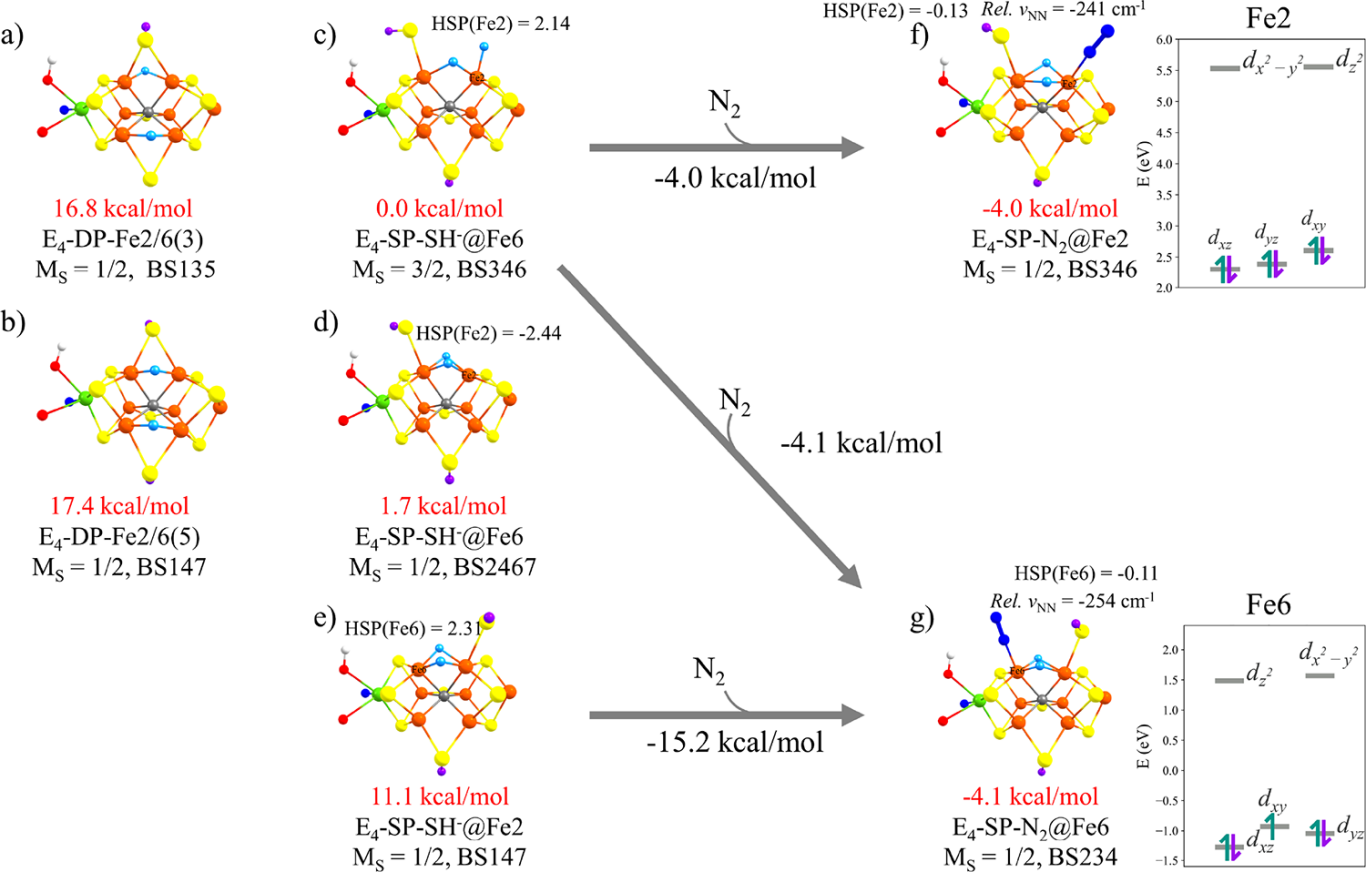
Structures of the **E_4_** models calculated (a–e) before N_2_ binding and (f, g) N_2_-bound **E_4_** models. The QRO-based ligand-field diagrams are shown for the labeled Fe. All calculated data used the large QM-VI region.

Here, on the other hand, we are interested in the binding of N_2_ to models of the **E_4_** state, and **E_4_-SP**, **E_4_-DP-Fe2/6(3)**, and **E_4_-DP-Fe2/6(5)** models are of interest. Figure 8 shows N_2_ binding energies to each of the **E_4_-DP-Fe2/6(3)**, **E_4_-DP-Fe2/6(5)**, and the **E_4_-SP** models relative to the most stable **E_4_** model calculated at the r^2^SCAN-QM/MM level of theory. **E_4_-SP** models and especially models with N_2_ bound to Fe6: **E_4_-SP-N_2_@Fe6** show considerably different N_2_ binding energies. As shown in Figure 9 (presenting the data with a large QM-VI region), a highly favorable single-step N_2_ binding energy of −15.2 kcal/mol is found for the **E_4_-SP-SH^–^@Fe2** → **E_4_-SP-N_2_@Fe6** binding. However, calculating the energy with respect to the most stable **E_4_** isomer found, **E_4_-SP-SH^–^@Fe6** (*M*_S_ = **3/2**, BS346) (shown in Figure 9c), the energy becomes −4.1 kcal/mol. A similar binding energy for **E_4_-SP-SH^–^@Fe6** → **E_4_-SP-N_2_@Fe2** is −4.0 kcal/mol, which shows that Fe2 and Fe6 binding sites cannot be so easily distinguished. In contrast, N_2_ binds to Fe2 and Fe6 in **E_4_-DP-Fe2/6(3)** and **E_4_-DP-Fe2/6(5)** models with single-step N_2_ binding energies close to zero kcal/mol (see Figure S18), which, however, is not an improvement over the **E_2_** models in Section 3.4, perhaps as the number of hydrides at the Fe2/Fe6 binding site remains the same.

Prior to N_2_ binding, these models feature local spin states of the 5-coordinated Fe ion of either intermediate-spin or high-spin states according to localized orbitals (see Figures S19 and S20). After N_2_ binding, creating effectively a 6-coordinated Fe ion, the ligand-field diagrams reveal an approximate octahedral ligand-field with a sizeable gap between t_2g_ and e_g_ orbitals. The *d_xz_* and *d_yz_* orbitals are again doubly occupied in the **E_4_-SP-N_2_@Fe6** and **E_4_-SP-N_2_@Fe2** states. The Hirshfeld spin populations are in good agreement with the orbital analysis, showing small spin populations on the N_2_-binding Fe ion, suggesting a local low-spin state, clearly due to the change in coordination enabled by the hydrides.

### 3.6 N_2_ Binding to **E_4_-SP** in Two α-His195 Protonation States

The protonation state of α-His195 is plausibly assigned as being protonated on the N_ε_ atom in the **E_0_** state according to the high-resolution X-ray crystal structure. However, it is unknown whether this protonation state persists for different **E_*n*_** states or whether the N_δ_ position could be protonated instead. This histidine residue has in fact been suggested to have a proton transfer role during the reduction of N_2_ according to mutation studies.^110^ If a proton transfers from N_ε_ to the cofactor (becoming either a hydride or S-bound proton), a simultaneous Grotthuss-type N_δ_ protonation could in fact occur, possibly via a water molecule hydrogen-bonding to N_δ_ in the X-ray structure. This would give an inverse protonation state of His195 (His195-N_δ_(H)) that could persist depending on its stability.

Our results show that the N_ε_ protonation state is more energetically favorable than the N_δ_ one by >15 kcal/mol for **E_4_-SP-SH^–^@Fe2**, **E_4_-SP-SH^–^@Fe6**, **E_4_-SP-N_2_@Fe2**, and **E_4_-SP-N_2_@Fe6** cases, shown in Table S6, which is consistent with previous results^12^ for **E_0_**, **E**_**1**,_ and **E_2_** states; however, the energy difference of these protonation isomers of His195 would be solely determined by the specific protein and cofactor environment, which is currently biased toward the **E_0_** state in our model (as our model is based on the resting state X-ray structure). It is thus not unreasonable to imagine that a slightly different environment present in a reduced cofactor state could change this protonation state preference. A future QM/MM dynamics study may be able to shed more light on the plausibility of this idea.

**Figure 10:**
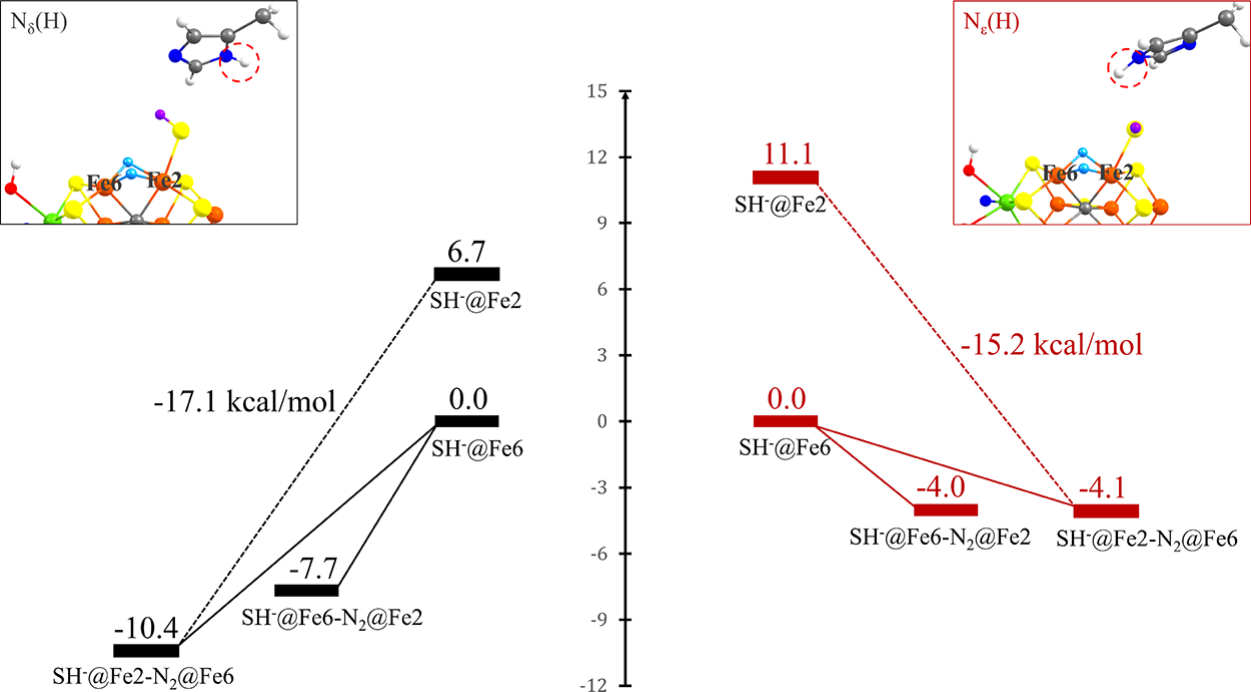
N_2_ binding energies for **E_4_-SP** models with a proton in either the N_δ_ position (left) or the N_ε_ position (right) of α-His195. Insets show the **E_4_-SP-SH^–^@Fe2** model in either α-His195 protonation states. Results were obtained using the QM-VI region. We note that the **E_4_-SP-SH^–^@Fe6** state with a His195-N_ε_(H) environment is calculated to be considerably lower than His195-N_δ_(H), by 22.3 kcal/mol. However, the computational model may be biased toward the **E_0_** crystal structure (where His195-N_ε_(H) is present).

Here, we explore how assuming a His195-N_δ_(H) protonation state instead would affect the binding of N_2_ to the **E_4_-SP** isomers. A doubly protonated His195 residue was also briefly explored (see the SI); however, seeing as spontaneous proton transfer from His195 to the cofactor occurs in that case, such a protonation state may not be realistic. Interestingly, Dance has previously described reduced FeMoco isomers containing a doubly protonated His195; the use of a cluster model (instead of a QM/MM model) with a different functional (BLYP instead of r^2^SCAN) is likely the reason for the different prediction.^111^Figure 10 compares how the relative energies of **E_4_-SP-SH^–^@Fe6** and **E_4_-SP-SH^–^@Fe2** isomers as well as N_2_ binding energies are affected by the His195 protonation state. First of all, the energy difference between the **E_4_-SP-SH^–^@Fe6** and **E_4_-SP-SH^–^@Fe2** isomers is reduced from 11.1 to 6.7 kcal/mol in going from His195-N_ε_(H) to His195-N_δ_(H). Second, the binding preference of N_2_ to either Fe2 or Fe6 sites is quite strongly affected by the His195 protonation state, with **N_2_@Fe2** and **N_2_@Fe6** states being about equally stable for His195-N_ε_(H), while for His195-N_δ_(H), stronger N_2_ binding is generally found as well as a clearer preference for binding to Fe6.

The reasons for these trends are not entirely clear but appear to arise due to steric and H-bonding effects involving SH^–^ and N_2_ with the histidine residue, depending on whether the Fe2 site has a terminal N_2_ or SH^–^ ligand bound.

Finally, we note that mutation studies where α-Val70 is mutated into α-Ile70 have shown to prevent N_2_ reduction from occurring.^45,46^ The crystal structure reveals that the additional methyl group in isoleucine compared to valine is positioned directly above the Fe6 site, introducing steric hindering. While the lack of N_2_ reduction in this variant suggests Fe6 as a likely potential binding site for N_2_, it is also possible that the bridging → terminal sulfhydryl group conversion (on either Fe2 or Fe6) seen in the **E_4_** isomers would be similarly sensitive to such steric hindering with the protonation state of His195 further complicating matters.

## 4 Discussion

**Figure 11:**
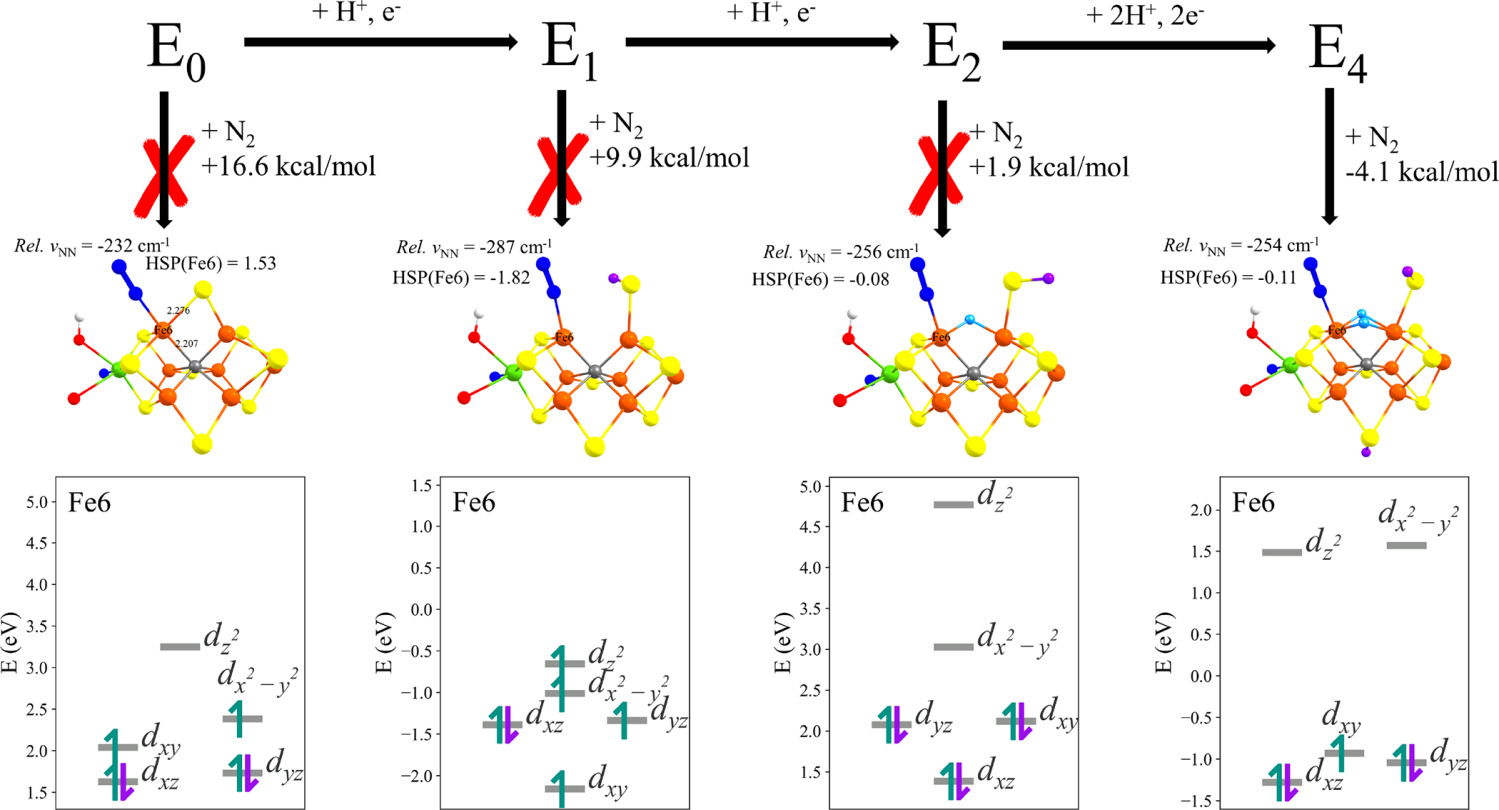
N_2_ binding energies, geometries, and QRO-based ligand-field diagrams of Fe6 for each **E_*n*_** states. These energies are the lowest in each **E_*n*_** state calculated using the QM-VI region. Results were calculated using the QM-VI region.

The results of this study highlight the fact that favorable N_2_ binding is actually rather difficult to achieve in calculations of FeMoco. The diamagnetic substitution results (Section 3.1) show that a sufficiently reduced Fe ion in the Fe(I) redox state will favorably bind and activate N_2_ but such a localized reduced Fe(I) state may not easily form in FeMoco with the strong tendency of the cluster to delocalize electrons over multiple Fe ions. Figure 11 shows the most favorable calculated N_2_-bound states for each redox state of FeMoco according to the results of the previous sections. FeMoco at the **E_0_** level will only barely give stable N_2_-bound local minima and only in a seemingly unfavorable distorted trigonal bipyramidal geometry of the N_2_-bound Fe ion (with elongated Fe–C and Fe–S bonds). The addition of a proton to the bridging S2B sulfide allows more flexibility in stabilizing N_2_-bound geometries that together with an additional electron in the **E_1_** state leads to a terminal N_2_-bound structure in a distorted tetrahedral/seesaw geometry. While the **E_1_-N_2_** structure shows moderate N_2_ activation, the binding energy remains unfavorable. However, adding yet another electron and localizing the two electrons in a bridging hydride between Fe2 and Fe6 in the **E_2_** state changes the picture, with a considerably more favorable N_2_-bound state, albeit still slightly endothermic. The addition of a single hydride as a ligand appears to enable the formation of a low-spin electron configuration on the N_2_-binding Fe that is now more favorable than before, even though the **E_2_-hyd-N_2_** state does not activate N_2_ more than the **E_1_-N_2_** state does. As the ligand-field diagrams in Figure 11 show, there is a common theme of spin-pairing in the *d_xz_* and *d_yz_* orbitals at the N_2_-bound Fe ion, even in the **E_0_-N_2_** state, that should enable backbonding to N_2_. However, such a spin-paired electron configuration (beneficial for N_2_ back-bonding) may well come at the cost of destabilizing the spin-coupled electronic structure of FeMoco (an Fe ion in a non-high-spin state will have much less favorable antiferromagnetic coupling interactions). The addition of strong σ-donating hydrides to FeMoco may be what makes such low-spin electron configurations more favorable, despite the cost of losing antiferromagnetic interactions. In fact, adding another hydride to FeMoco, i.e., going to the **E_4_** state, but importantly adding the hydride to the same pair of Fe ions (rather than different pairs of Fe ions as in previously proposed **E_4_** models), enables the formation of a distorted octahedral N_2_-bound state in a low-spin electronic configuration (again with *d_xz_* and *d_yz_* orbitals doubly occupied). Interestingly, this N_2_-bound state features the N_2_-bound Fe ion in a *S* = 1/2 Fe^3+^ oxidation state (i.e., more oxidized than the Fe^2+^ ions in the other redox states), but unlike the other redox states, the N_2_ binding has now become thermodynamically favorable (based on the electronic energy alone, not free energy). It seems that the addition of two hydrides in the **E_4_** state have now allowed a favorable low-spin electron configuration to form, with occupation of *d_xz_* and *d_yz_* orbitals, which is useful for N_2_ binding, but this time presumably without the associated energy penalty of forming such a configuration like in the other redox states. In this context, it is noteworthy that Peters and co-workers have synthesized two low-spin Fe_2_(μ-H)_2_ complexes (either 2Fe(II) or Fe(II)Fe(I)) capable of binding N_2_ with a local octahedral geometry.^112^ The 2Fe(II) complex with one bound N_2_ gives a similar N_2_ frequency shift of Δ = −264 cm^–1^ (relative to free N_2_), as found in our **E_4_-SP-N_2_@Fe6** model (Δ = −254 cm^–1^). There is nonetheless a difference in the coordination environment: sulfide vs phosphine ligation, carbon vs silicon axial ligand, but also the hydride geometries differ (more asymmetric Fe–H bridges in the cofactor model, more acute <H-Fe-H angles and a non-planar Fe_2_(μ-H)_2_ unlike the complexes). It is also worth noting the rich literature describing synthetic Fe complexes with N_2_ ligands with increasingly more biomimetic ligand environments.^112−120^ In a particularly relevant study, Suess and McSkimming described a reduced [MoFe_3_S_4_]^1+^ cubane,^119^ analogous to the FeMoco [MoFe_3_S_3_C] subcubane, with an Fe-bound N_2_ ligand (and substantially activated). Additionally, a [Fe_4_S_4_] cubane with a single terminal CO ligand has recently been described, and spectroscopy reveals the CO-bound Fe to be a low-valent Fe^1+^ site with double-occupation of the backbonding *d_xz_*, *d_yz_* orbitals.^121^ It is clear that a general model for what determines the binding affinity of N_2_ to terminal Fe sites is not complete; overall, the literature suggests that low-spin Fe sites are preferred, together with electron-donating ligands and reduced oxidation states. Our results are mostly in agreement with this consensus, with the local Fe(III) configuration found at Fe6 in our **E_4_-SP-N_2_@Fe6** model being an exception; we suspect that this relates to the difference between the determined d-electron configuration (assuming idealized electron-counting) and the effective local Fe charge in the cluster (with highly covalent Fe–H bonds present).

A common theme in the results as presented in Figure 11 is the general preference of N_2_ binding to Fe6 rather than Fe2. This effect is seemingly at least partly due to Fe6 being more sterically accessible; however, this is further complicated by the fact that both N_2_-bound states and hydride-containing states stabilize an open S2B belt sulfide bridge with a terminal sulfhydryl group on either Fe6 or Fe2, which causes a similar steric effect. The closeness of the His195 residue is the key here, a residue that has been implicated as both a proton-transfer residue and being important to N_2_ binding. With the results also rather dependent on the protonation state of His195, as shown in Section 3.6, it thus seems difficult to convincingly distinguish between Fe2 and Fe6 at this stage. Fe6 is arguably, however, a more interesting binding site, being close to the Mo ion as well as the homocitrate ligand with a protonated alcohol group aptly positioned for possible proton transfer to the N_2_ ligand. Another QM/MM study furthermore has found protonated N_2_-substrates such as diazene to be thermodynamically more stable in the Fe6 position,^54^ revising a previous study^122^ that favored binding to Fe2. While the use of a QM/MM model in our calculations is important to describe a realistic electrostatic and steric environment around the cofactor, we note that much simpler cofactor-only (same size as QM-I region) calculations with a continuum model (using the same functional and basis set) were also performed for N_2_ binding to **E_0_**, **E_1_**, **E_2_-hyd**, and **E_4_-SP** models in Figure 11. This data, shown in Figure S24 in the SI, reveal the same trend as the QM/MM data, giving further evidence for the importance of hydride ligation for favorable N_2_ binding energies. The differences between cluster and QM/MM data (primarily affecting Fe2 binding) can be attributed to steric effects.

**Figure 12:**
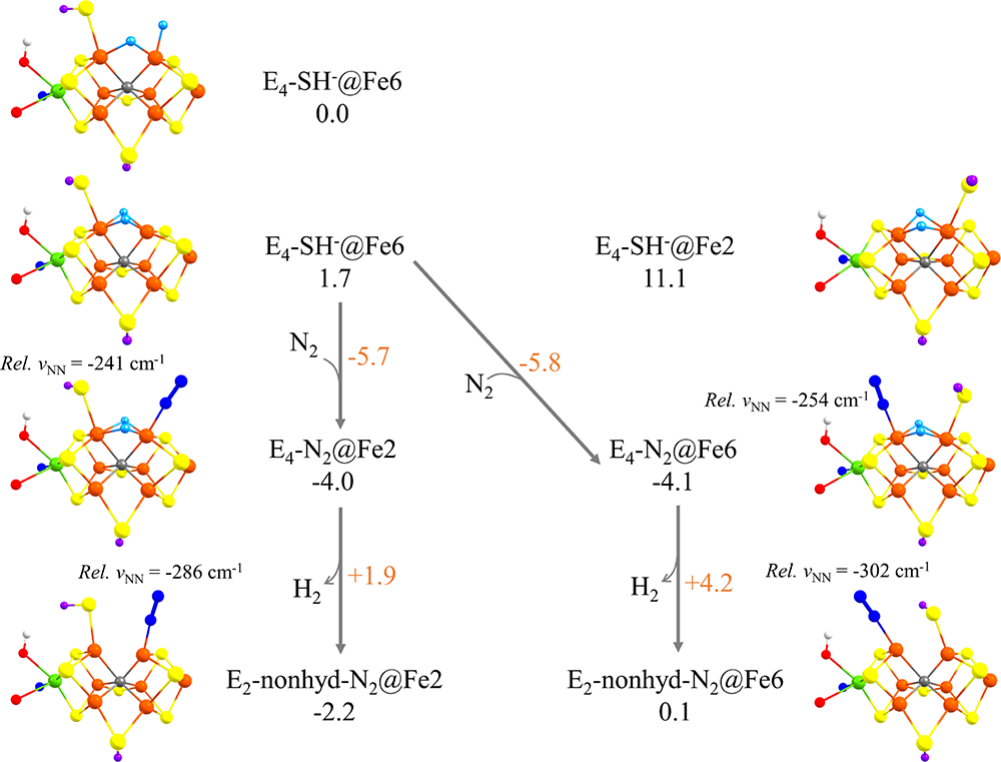
Binding of N_2_ to both Fe2 and Fe6 sites in the **E_4_-SP** models followed by reductive elimination of two hydrides of **E_4_-SP**. The His195-N_ε_(H) protonation state is assumed. Calculated results used the QM-VI region.

Experimentally, it is now well established that a molecule of H_2_ is formed as N_2_ binds to the **E_4_** state and isotope labeling has shown that H_2_ is derived from the two hydrides, a kinetic step that only occurs when N_2_ is present (without N_2_, **E_4_** will instead relax by H_2_ evolution to the **E_2_** state via a regular hydride + proton mechanism).^37,38,62^Figure 12 shows the results of N_2_ binding to our **E_4_-SP** models, in both Fe2 and Fe6 positions, followed by the energy of performing the reductive elimination step. Consistent with experiments, the H_2_ evolution via reductive elimination is neither strongly endothermic nor exothermic but rather close to being thermoneutral instead (in both Fe2 and Fe6 positions). While the overall reaction energy for **E_4_** + N_2_ → **E_4_-N_2_′** + H_2_ is slightly more favorable at the Fe2 position, N_2_ is in contrast more activated at the Fe6 position.

The nature of the calculated **E_4_-N_2_′** state after reductive elimination has occurred (whether at Fe2 or Fe6 position) is interesting. As 4 e^–^ and 4 H^+^ are present in FeMoco in the **E_4_** state of FeMoco (relative to **E_0_**) but reductive elimination removes 2 e^–^ and 2 H^+^ via the two hydrides as H_2_, the redox state is now identical to a nonhydridic **E_2_** state with N_2_ bound to Fe2 or Fe6 with an open belt sulfide bridge. Such a state was in fact previously calculated and discussed in Section 3.4 when we considered N_2_ binding to **E_2_-nonhyd** models. The **E_2_-nonhyd** state may be related to the experimentally described E_4_(2H)*, a state formed after photoinduced formation of H_2_ from two hydrides.^37^ We note that **E_2_-nonhyd** models are in fact not interesting from the point of view of showing any favorable N_2_ binding; they are in fact strongly endothermic for N_2_ binding, but counterintuitively, the **E_2_-nonhyd-N_2_** models instead showed the most activated N_2_ substrates of all the states calculated in this work. The most activated N_2_ substrate is in fact found in the **E_2_-nonhyd-N_2_@Fe6** state, which is shown in Figure 12, and data for several electronic states of this model is shown in Figure 6 and discussed in Section 3.4. The relevance of this state, **E_2_-nonhyd-N_2_@Fe6**, now seems clearer: it is not favorable to bind N_2_ to the **E_2_** redox state directly (even though a nonhydridic **E_2_** state leads to the strongest N_2_ activation); instead, we must first proceed to the **E_4_** redox state where the thermodynamically favored double-hydride geometries both enable favorable N_2_ binding via stabilizing low-spin electronic configurations and also allow access to the N_2_-activating **E_2_-nonhyd** state via the reductive elimination step involving the hydrides.

It is unclear what happens next. The **E_2_-nonhyd-N_2_** state having such low affinity for N_2_ according to our calculations should dispel the substrate (unless trapped behind a barrier). Perhaps, more likely the N_2_ ligand is promptly converted to a more favorable protonated form (N_2_H or N_2_H_2_), though this would seemingly conflict with the known reversible N_2_/H_2_ exchange step in the **E_4_** state.^44^ A recent kinetic study^9^ suggests that a slow kinetic step after N_2_ binding/H_2_ evolution occurs, which might be assigned to a slow N_2_ activation/protonation process. Previous calculations from our group suggested that diazene formation (via SH and OH) might already form, although it was calculated to be slightly uphill (+5.6 kcal/mol).^14^ Clearly, mysteries remain about the nature of this key mechanistic step.

## 5 Conclusions

We have systematically investigated N_2_ binding to the FeMo cofactor of nitrogenase using a QM/MM model to unravel the nature of how and why N_2_ binds and gets activated for protonation. Critical to this work is the use of detailed electronic structure analysis of each broken-symmetry DFT state calculated and the correlation with calculated N_2_ binding energies to each redox state of the cofactor. Using a combination of localized orbital analysis, Hirshfeld spin populations, and ligand-field diagrams derived from quasi-restricted orbitals (made possible via diamagnetic substitution), we could derive cofactor electron configurations but also obtain a ligand-field level insight into the local Fe spin state changes occurring as a function of hydride and N_2_ coordination in the models calculated.

This study builds on models for the **E_2_** and **E_4_** states previously introduced by our research group^12,14^ but are here analyzed in more detail and compared with the **E_0_** and **E_1_** states. The results strongly suggest that it is double-hydride coordination to Fe2 and Fe6 that might be responsible for an increased binding affinity of N_2_ to the cofactor by stabilizing a local low spin state in the N_2_-bound state. Overall, this chemically plausible model offers an explanation for why hydride coordination in **E_4_** allows N_2_ binding to occur and furthermore how reductive elimination could be triggered by N_2_ binding. Importantly, we also show that the trend of more favorable N_2_ binding energy with the **E_*n*_** redox state is reproduced not only with our QM/MM model but also with a QM-I cluster model of FeMoco (lacking the protein environment). This suggests that it is primarily the electronic structure of the cofactor that explains the most basic mechanism of how N_2_ might bind to cofactor while the protein environment is likely to influence precisely to which Fe ion N_2_ will bind.

There are important caveats to our calculations, however. The results reveal favorable N_2_ binding based on electronic energies (approximate enthalpies). It is unclear whether the calculated binding is able to overcome the loss of translational entropy (may also be offset by H_2_ evolution), which, based on gas-phase estimates, would be on the order of 10–15 kcal/mol. Recent work by Dance, however, has suggested a lower ∼4 kcal/mol entropic penalty based on N_2_ binding from a diffusible position within the protein.^59,123^ Future free energy simulations will hopefully be able to clarify this. The problem of DFT-method dependency on the results of FeMoco calculations is still far from being fully understood. In this work, we used the r^2^SCAN functional that our previous benchmarking study^74^ identified as the best performing density functional for the geometric features of iron–sulfur and iron–molybdenum–sulfur spin-coupled dimers as well as FeMoco itself. Future benchmarking of relevant reaction energies would be desired to estimate better the uncertainty associated with the energies. We also note that the results of this work have revealed local spin-state changes to be especially critical to the stability of N_2_-bound species. Encouragingly, r^2^SCAN is known to be an accurate DFT method according to benchmarking studies of Fe spin crossover complexes.^81^

The calculated N_2_-bound species in this work show only weak-to-moderate N_2_ activation rather than strong N_2_ activation, as defined by Studt and Tuczek.^101^ While strong N_2_ activation (Δν_NN_ = ∼1000 cm^–1^), typically requiring bridging M-N_2_-M interactions, might be expected to be necessary to activate N_2_ for protonation, successful stoichiometric and catalytic N_2_ fixation has been achieved for complexes with only moderately activated N_2_.^124^ In a recent study^125^ by Peters and co-workers on N_2_ reduction catalysis with molecular Fe/Mo compounds, the use of a concerted proton-electron transfer mediator, which enables favorable H-atom transfer to the substrate, even avoids strongly reduced metal ion species (that are known to activate N_2_ more strongly) and allows H-addition to metal-bound N_2_ seemingly without ever activating N_2_ strongly. The details of concerted proton-electron transfer to FeMoco are, however, not well understood. The level of N_2_ activation in the most favorable N_2_-bound FeMoco species discussed in this work is (Δν_NN_ = 302 cm^–1^) on par with the level of N_2_ activation found in, e.g., the Mo-bound catalytic Schrock complex (Δν_NN_ = 340 cm^–1^),^101,126^ previously classified as an example of moderate N_2_ activation. Much more work is required before the mechanism of nitrogenase can be claimed to be understood, but it is our hope that this study represents a step toward a more detailed understanding of the electronic structure basis of biological N_2_ fixation.

## Supporting Information Available

Additional information on QM-regions, electronic structure analysis, orbitals, geometries, broken-symmetry states, and relative energies (PDF)
Coordinates of QM-regions (ZIP)
