# Supplementary material for: Understanding the Electronic Structure Basis for N2 Binding to FeMoco: A Systematic Quantum Mechanics/Molecular Mechanics Investigation
Source: Inorg Chem. 2023 Mar 29;62(14):5357–75. doi: 10.1021/acs.inorgchem.2c03967 (PMC10091479; doi:10.1021/acs.inorgchem.2c03967)
Supplement: Supplementary file 1 — ic2c03967_si_001.pdf [file ic2c03967_si_001.pdf]

Supporting Information

Understanding the electronic structure basis for N_2 binding to FeMoco: a systematic quantum mechanics / molecular mechanics investigation

Yunjie Pang[a,b] and Ragnar Bjornsson[b,c*]

[a]College of Chemistry, Beijing Normal University, 100875, Beijing, China.

[b]Max-Planck Institute for Chemical Energy Conversion, Stiftstrasse 34-36, 45470 Mülheim an der Ruhr, Germany.

[c]Univ. Grenoble Alpes, CNRS, CEA, IRIG, Laboratoire de Chimie et Biologie des Métaux, 17 Rue des Martyrs, F-38054 Grenoble, Cedex, France. E-mail: ragnar.bjornsson@cea.fr

Contents

1. QM/MM model and region definitions 3

2. Analysis of relative energy differences with different QM regions 4

3. N_2 binding to diamagnetically substituted FeMoco models: geometries, binding energies, relative frequencies, QRO-based ligand field diagrams, and quasi-restricted orbitals (QROs) 6

4. N_2 binding to E_0 and E_0H^+ models: structures, QRO-based ligand-field diagrams, and localized orbital analysis 7

5. N_2 binding to a cluster-continuum model of E_0: structures, binding energies 9

6. N_2 binding to the E_1 state: structures, binding energies, QRO-based ligand-field diagrams, and electronic configurations from localized orbital analysis 10

7. CO binding to Fe6 of the E_1 state: binding energies 11

8. N_2 binding to the E_2 state: structures, binding energies, QRO-based ligand-field diagrams, and electronic configurations based on the localized orbital analysis 12

9. N_2 binding energies, single-step binding energies, and relative N-N frequencies for E_0, E_0H^+, E_1, E_2, and E_4 states 15

10. N_2 binding to the E_4 state: structures, N_2 binding energies, single-step N_2 binding energies, QRO-based ligand-field diagrams, and electronic configurations from localized orbital analysis 17

11. Testing additional BS solutions for E_4 models 20

12. The Hirshfeld charges and populations of distal N and Fe2 or Fe6 when N_2 binding 22

13. The N_2 binding affinities from E_0 to E_4 states using cluster models 23

14. The effect of different His195 protonation states for N_2 binding to the E_4-SP model 24

15. How the QRO-based ligand-field diagrams were created 26

16. Spin-state energies of diamagnetically substituted models 28

17. The energy difference between E_2-hyd and E_2-nonhyd models 31

18. Electronic structure sensitivity of the E_2-nonhyd isomer 33

19. Additional N_2 binding modes 34

20. Investigation of the integration grid dependence in r^2SCAN calculations 35

21. Relative energies, vibrational frequencies and Hirshfeld spin populations of all calculated models 36

References 39

1. QM/MM model and region definitions

Figure S1 shows the full QM/MM model, the active and QM regions of the models used in the article. Table S1 shows the composition of each QM-region.

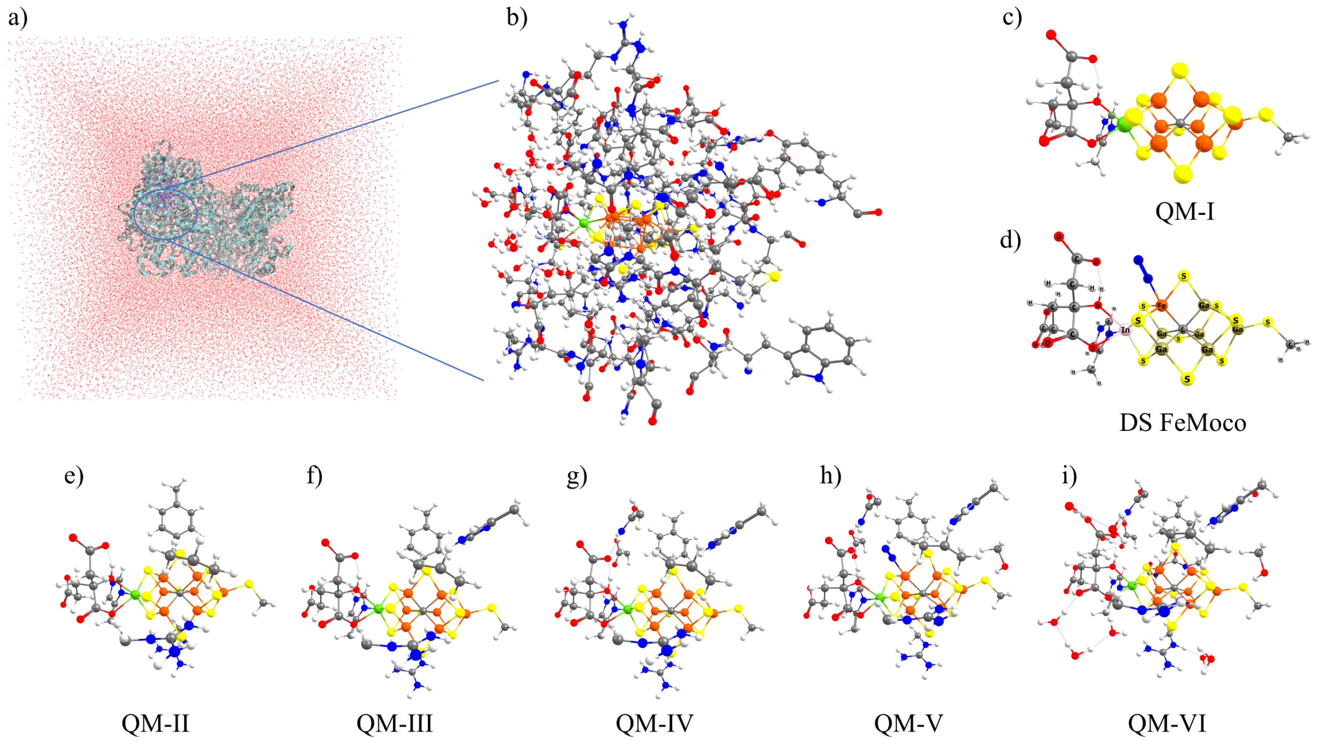

Figure S1. (a) The full QM/MM model. (b) The active region (~990 atoms). (c-d) The QM-I region in full FeMoco (c) and diamagnetically substituted (DS) FeMoco (d). (e-i) Larger QM regions. The coordinates of the active, QM-I, QM-V, and QM-VI regions of $\mathbf{E_0}$ without N_2 were given in SI.

Table S1. Definitions of the QM-regions (assuming $\mathbf{E_0}$). Residues are located in the α subunit.

Regions	No. atoms (link-atoms included)	Region definition
**QM-I**	56	FeMoco + homocitrate + His442 (4-MEI) + Cys275 (S-Me)
**QM-II**	108	QM-I + Val70 (Pr) + Arg96 (guanidyl-Me) + Arg359 (guanidyl-Me) + Phe381 (phenyl-Me)
**QM-III**	120	QM-II + His195 (4-MEI)
**QM-IV**	136	QM-III + Gln191 (Ethanamide) + Glu380 (Me-COO$^-$)
**QM-V**	142	QM-IV + Ser278 (HO-Me)
**QM-VI**	188	QM-V + Gly356 (backbone) + Gly357 (backbone) + 10 H_2O

2. Analysis of relative energy differences with different QM regions

The relative energies of some reduced FeMoco isomers were found to be sensitive to the QM-region size, in particular the MM energy difference was sometimes found to be large in magnitude. Here we analyze this sensitivity by comparing the energy difference between the non-hydride and hydride isomers of the E_2 redox state with N_2 bound to Fe6: **E_2-hyd-N_2@Fe6-BS235 (M_S = 1/2)** (Figure 7c) and **E_2-nonhyd-N_2@Fe6-BS235 (M_S = 3/2)** (Figure S11b). The MM energy contribution, **ΔE(MM)**, is suspiciously large, 9.1 kcal/mol using the QM-I region which increases to 10.0 kcal/mol when the QM-V region is used, as shown in Table S2. Only when the QM-VI region was used (see QM-VI-OPT data in the Table S2) did the **ΔE(MM)** contribution drop down below 4 kcal/mol. Separate calculations (Table S2) indicate that the difference is both due to Gly356 and Gly357 residues (including the backbone) as well as 10 H_2O. The results clearly reveal that despite the large MM energy contribution in the smaller QM-region, the total QM/MM energy difference remains fairly reliable.

Optimizing the QM-VI region is costly as the QM region contains more than 180 atoms. However, performing single-point calculations at the QM-VI level on the smaller QM-V geometry turns out to be a good compromise.

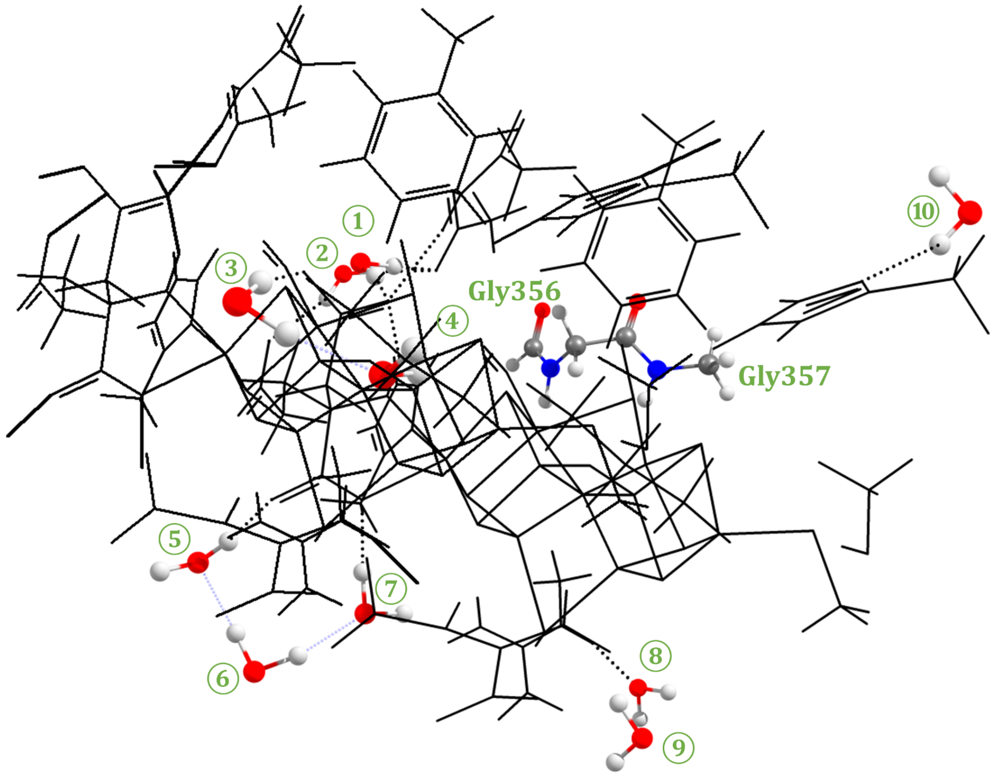

Figure S2. The comparison of the QM-V (black lines) and QM-VI regions (black lines + ball&stick). Every water molecule was numbered and used in Table S2.

Table S2. Relative energies (QM, MM, and QM/MM) of **E_2-hyd-N_2@Fe6-BS235** and **E_2-nonhyd-N_2@Fe6-BS235** comparing different QM-regions and Single-point (SP) vs. optimized (OPT) types. Every water molecule was numbered in Figure S2.

	ΔE(polarized QM)	ΔE(MM)	ΔE(QM/MM)
**E_2-hyd-N_2@Fe6-BS235-QM-I-OPT (M_S = 1/2)**	0.0	0.0	0.0
**E_2-nonhyd-N_2@Fe6-BS235-QM-I-OPT (M_S = 3/2)**	13.4	9.1	21.2
**E_2-hyd-N_2@Fe6-BS235-QM-V-OPT (M_S = 1/2)**	0.0	0.0	0.0
**E_2-nonhyd-N_2@Fe6-BS235-QM-V-OPT (M_S = 3/2)**	9.3	10.0	19.3
**E_2-hyd-N_2@Fe6-BS235-QM-(V+2,3,4)-SP (M_S = 1/2)**	0.0	0.0	0.0
**E_2-nonhyd-N_2@Fe6-BS235-QM-(V+2,3,4)-SP (M_S = 3/2)**	11.4	7.5	18.9
**E_2-hyd-N_2@Fe6-BS235-QM-(V+1,2,3,4)-SP (M_S = 1/2)**	0.0	0.0	0.0
**E_2-nonhyd-N_2@Fe6-BS235-QM-(V+1,2,3,4)-SP (M_S = 3/2)**	12.1	6.7	18.7

**E_2-hyd-N_2@Fe6-BS235-QM-(V+2,3,4,10)-SP (M_S = 1/2)**	0.0	0.0	0.0
**E_2-nonhyd-N_2@Fe6-BS235-QM-(V+2,3,4,10)-SP (M_S = 3/2)**	12.0	6.9	18.9
**E_2-hyd-N_2@Fe6-BS235-QM-(V+1,2,3,4,5,6,7)-SP (M_S = 1/2)**	0.0	0.0	0.0
**E_2-nonhyd-N_2@Fe6-BS235-QM-(V+1,2,3,4,5,6,7)-SP (M_S = 3/2)**	12.8	6.1	18.9
**E_2-hyd-N_2@Fe6-BS235-QM-(V+1,2,3,4,5,6,7,8,9)-SP (M_S = 1/2)**	0.0	0.0	0.0
**E_2-nonhyd-N_2@Fe6-BS235-QM-(V+1,2,3,4,5,6,7,8,9)-SP (M_S = 3/2)**	13.3	5.0	18.3
**E_2-hyd-N_2@Fe6-BS235-QM-(V+Gly356+Gly357)-SP (M_S = 1/2)**	0.0	0.0	0.0
**E_2-nonhyd-N_2@Fe6-BS235-QM-(V+Gly356+Gly357)-SP (M_S = 3/2)**	10.1	9.2	19.3
**E_2-hyd-N_2@Fe6-BS235-QM-VI-OPT (M_S = 1/2)**	0.0	0.0	0.0
**E_2-nonhyd-N_2@Fe6-BS235-QM-VI-OPT (M_S = 3/2)**	13.8	3.8	17.6
**E_2-hyd-N_2@Fe6-BS235-QM-VI-SP (M_S = 1/2)**	0.0	0.0	0.0
**E_2-nonhyd-N_2@Fe6-BS235-QM-VI-SP (M_S = 3/2)**	14.8	3.6	18.3
**E_2-hyd-N_2@Fe6-BS235-QM-VI-SP-TZVP@homocitrate[a] (M_S = 1/2)**	0.0	0.0	0.0
**E_2-nonhyd-N_2@Fe6-BS235-QM-VI-SP- TZVP@homocitrate[a] (M_S = 3/2)**	15.2	3.6	18.8

Note: [a] The homocitrate was calculated using ZORA-def2-TZVP, but homocitrate of other results in this table were calculated using ZORA-def2-SVP. The last two rows show that the difference between using ZORA-def2-TZVP and ZORA-def2-SVP is very small for the polarized QM energy and total QM/MM energy.

3. N_2 binding to diamagnetically substituted FeMoco models: geometries, binding energies, relative frequencies, QRO-based ligand field diagrams, and quasi-restricted orbitals (QROs)

Additional data on N_2 binding to diamagnetically substituted FeMoco, presented in Figure 2 of the manuscript. Figure S3 shows ligand-field diagrams of all stable N_2-bound states.

The diamagnetically substituted cluster-model was based on the optimized **E_0-BS235 (M_S = 3/2)** QM/MM model using the QM-I region with the MM pointcharge environment replaced by a CPCM polarizable continuum model. All metal atoms except Fe6 were replaced with a diamagnetic ion (Ga^{3+} for Fe and In^{3+} for Mo). Only Fe6 and N_2 were allowed to move during optimizations. High-spin states for Fe^{3+} and Fe^{2+} redox states did not bind N_2 while lower spin states did. Quasi-restricted orbital (QRO) analysis for all stable N_2-bound states reveals a common theme of spin-pairing in d_{xz} and d_{yz} orbitals. Only Fe^{1+} in M_S = 1/2 and 3/2 states gives exothermic N_2 binding energies.

Table S3 shows Hirshfeld charges and spin populations of all stable states before and after N_2 binding.

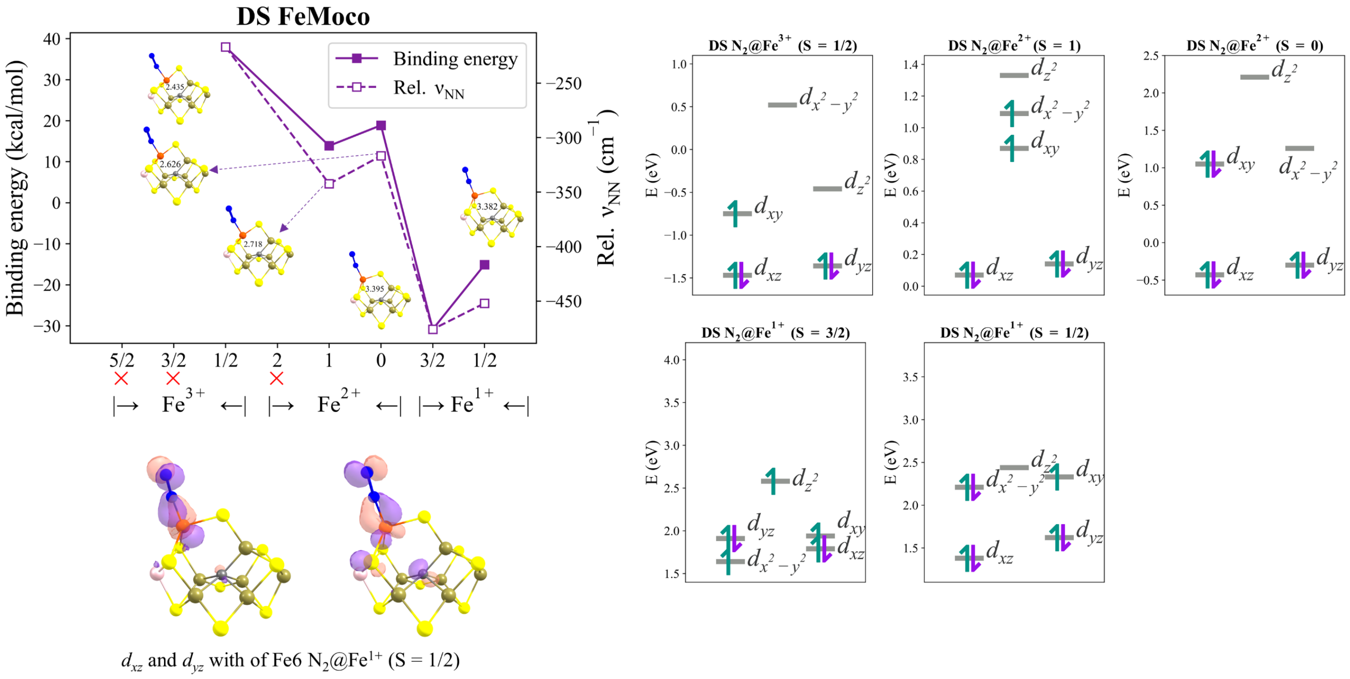

Figure S3. The upper left plot shows binding energies of N_2 to diamagnetically substituted (DS) FeMoco (with Fe6 unsubstituted) and relative N-N frequencies (relative to free N_2, $\nu_{NN,\ free}$ = 2432 cm^{-1}) as a function of redox state and spin-state of Fe6. Cross-symbols indicate spontaneous N_2 dissociation from DS FeMoco. Ligand-field diagrams of Fe6 in the stable N_2-bound states according to a quasi-restricted orbital (QRO) transformation of the full DS-model FeMoco. Also shown are d_{xz} and d_{yz} quasi-restricted orbitals of the diamagnetically substituted FeMoco in the Fe^{1+}(S = 1/2) state, revealing significant overlap with the π* orbitals of N_2.

Table S3. Hirshfeld charges and spin populations of Fe6 before and after N_2 binding.

	**Hirshfeld Charges of Fe6**	**Hirshfeld Spin populations of Fe6**
**Fe^{3+}-N_2 (S = 1/2)**	0.05	0.86
**Fe^{2+}-N_2 (S = 1)**	0.01	1.77
**Fe^{2+}-N_2 (S = 0)**	0.02	0.00
**Fe^{1+}-N_2 (S = 3/2)**	-0.02	2.83
**Fe^{1+}-N_2 (S = 1/2)**	-0.06	1.00
**Fe^{3+}-nonN_2 (S = 5/2)**	0.13	3.44
**Fe^{3+}-nonN_2 (S = 3/2)**	0.06	2.47
**Fe^{3+}-nonN_2 (S = 1/2)**	0.04	0.69
**Fe^{2+}-nonN_2 (S = 2)**	-0.01	3.04
**Fe^{2+}-nonN_2 (S = 1)**	-0.06	1.59
**Fe^{2+}-nonN_2 (S = 0)**	-0.05	0.01
**Fe^{1+}-nonN_2 (S = 3/2)**	-0.15	2.54
**Fe^{1+}-nonN_2 (S = 1/2)**	-0.18	0.93

4. N_2 binding to E_0 and E_0H^+ models: structures, QRO-based ligand-field diagrams, and localized orbital analysis

Additional data on N_2 binding to the $\mathbf{E_0}$ and $\mathbf{E_0H^+}$ models presented in the 2nd Results Section of the manuscript. Figure S4 reveals QRO-based ligand-field diagrams for Fe2 after N_2 binding in states **E_0-BS235 (M_S = 3/2)**.

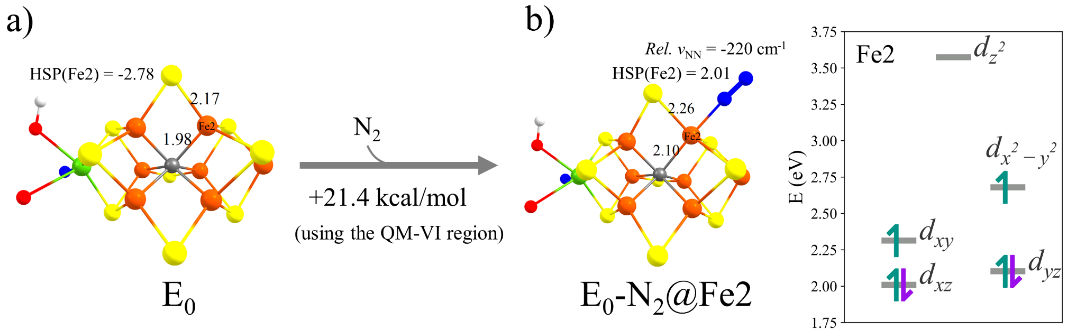

Figure S4. (a) Structures and Hirshfeld spin population (HSP) of Fe2 for the lowest-energy isomer **E_0-BS235** with M_S = 3/2. (b) The structure, relative N-N frequencies (relative to free N_2, $\nu_{NN,\ free}$ = 2432 cm^{-1}), and QRO-based ligand-field diagram of Fe2 for **E_0-N_2@Fe2-BS147** with M_S = 1/2.

Figure S5 shows the Fe and Mo electron configurations in FeMoco of three BS7 solutions before N_2 binding to the $\mathbf{E_0}$ state according to the localized orbital analysis. The difference between these three BS7 solutions is the location of localized high-spin Fe^{3+} and mixed-valence pairs. For example, the localized Fe^{3+} is at site Fe5, Fe7, or Fe6 for BS235, BS247, or BS346, respectively.

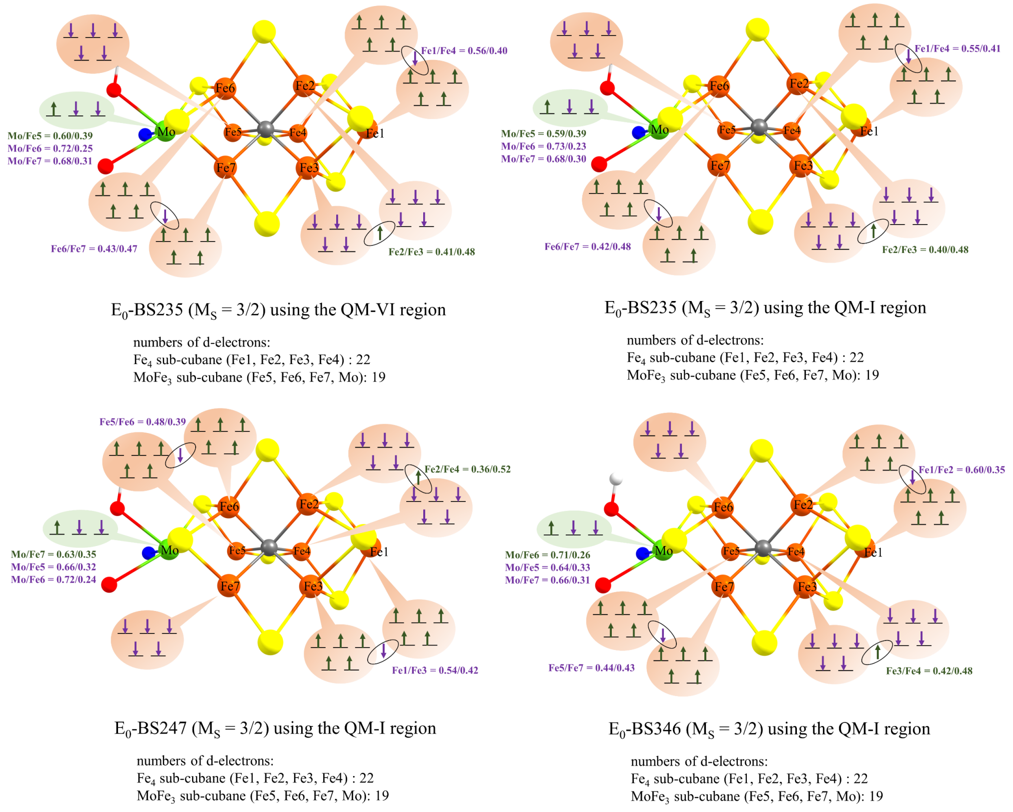

Figure S5. Electronic configurations based on the localized orbital analysis of three BS7 (BS235, BS247, and BS346) solutions of the $\mathbf{E_0}$ state before N_2 binding. Data using QM-VI and QM-I regions for BS235 solution reveal a relative insensitivity of the localized orbital population w.r.t. the QM-region. Also shown are electron-counts per cubane.

Figure S6 shows the Fe and Mo electron configurations in the FeMoco $\mathbf{E_0}$ redox state before and after N_2 binding to Fe6 according to the localized orbital analysis.

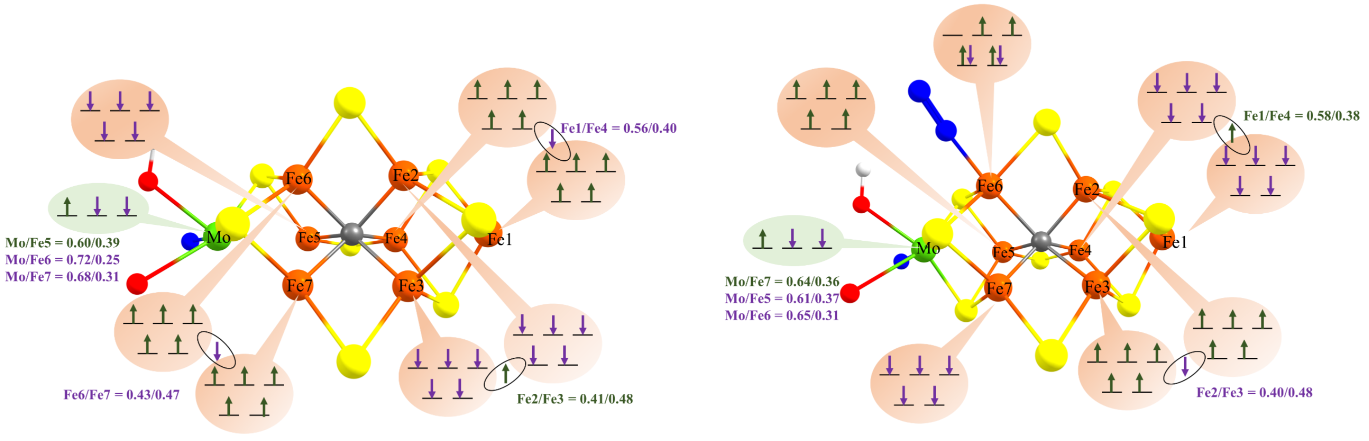

E_0-BS235 (M_S = 3/2)

numbers of d-electrons:
Fe_4 sub-cubane (Fe1, Fe2, Fe3, Fe4) : 22
$MoFe_3$ sub-cubane (Fe5, Fe6, Fe7, Mo): 19

E_0-N_2@Fe6-BS147 (M_S = 1/2)

numbers of d-electrons:
Fe_4 sub-cubane (Fe1, Fe2, Fe3, Fe4) : 22
$MoFe_3$ sub-cubane (Fe5, Fe6, Fe7, Mo): 19

Figure S6. Electron configurations of FeMoco before and after N_2 binding in the $\mathbf{E_0}$ state: **E_0-BS235 (M_S = 3/2)** and **E_0-N_2@Fe6-BS147 (M_S = 1/2)** according to localized orbital analysis. These two configurations were calculated using the QM-VI region. Localized orbital populations are shown for the Mo electrons and the Fe d-electrons with significant delocalization. Also shown are electron-counts per cubane.

5. N_2 binding to a cluster-continuum model of E_0: structures, binding energies

Figure S7 shows N_2 binding energies when N_2 binding to Fe2 or Fe6 of the $\mathbf{E_0}$ state of a cofactor-only (QM-I) cluster-continuum model of FeMoco. As protein residues around the FeMoco are not included in this cluster model, we can understand the effect of α-His195 when comparing the binding energies of N_2 binding to Fe2 or Fe6. Interestingly, the energy of N_2 binding to Fe2 is smaller than to Fe6, which is opposite to the results when using QM/MM level (see Figure 3 and Figure S4).

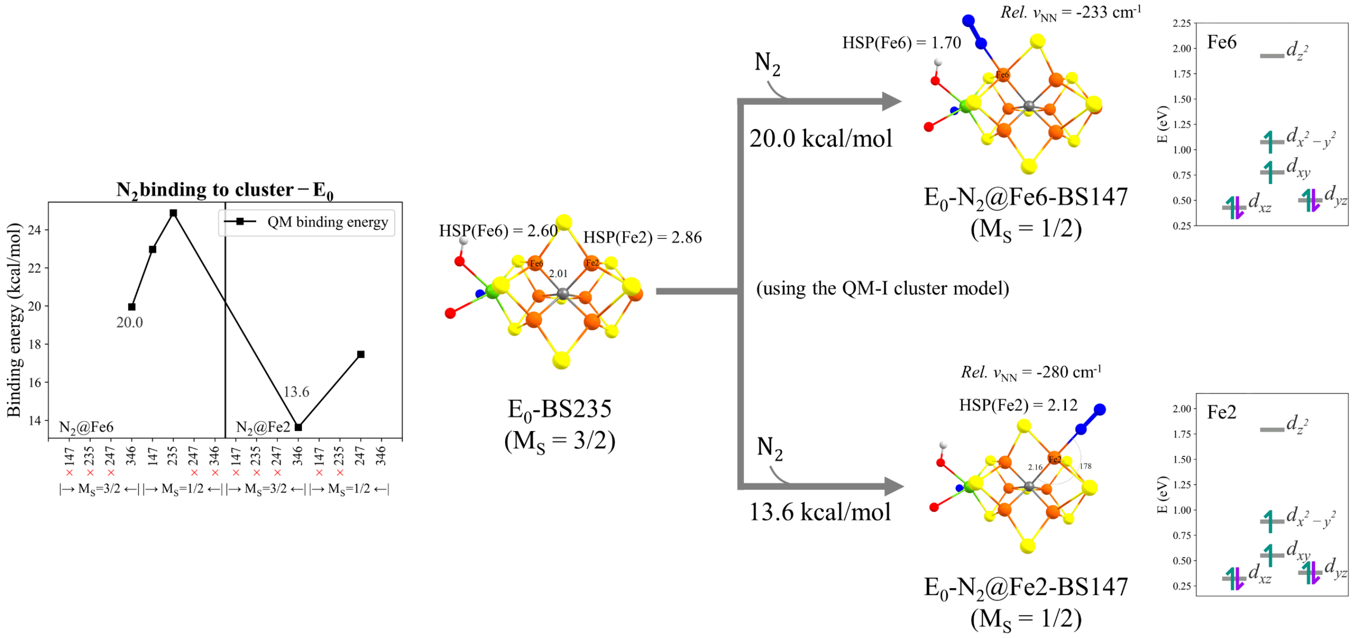

Figure S7. The QM binding energies, structures, Hirshfeld spin populations (HSP), and ligand-field diagrams when N_2 binding to QM-I cluster models of the $\mathbf{E_0}$ state. The cluster model was based on optimized $\mathbf{E_0}$**-BS235 (**$\mathbf{M_S = 3/2}$**)** using the QM-I region. Only the FeMoco atoms and N_2 were allowed to move during optimizations.

6. N_2 binding to the E_1 state: structures, binding energies, QRO-based ligand-field diagrams, and electronic configurations from localized orbital analysis

Additional results on N_2 binding to the $\mathbf{E_1}$ state, presented in the 3rd Results-Section of the manuscript. Figure S8 shows QRO-based ligand-field diagrams and Hirshfeld spin populations for Fe2 in the most favorable $\mathbf{E_1}$ isomer after N_2 binding to Fe2.

Figure S9 shows the electron configurations based on the localized orbital analysis of FeMoco in the $\mathbf{E_1}$ state before and after N_2 binding to Fe6.

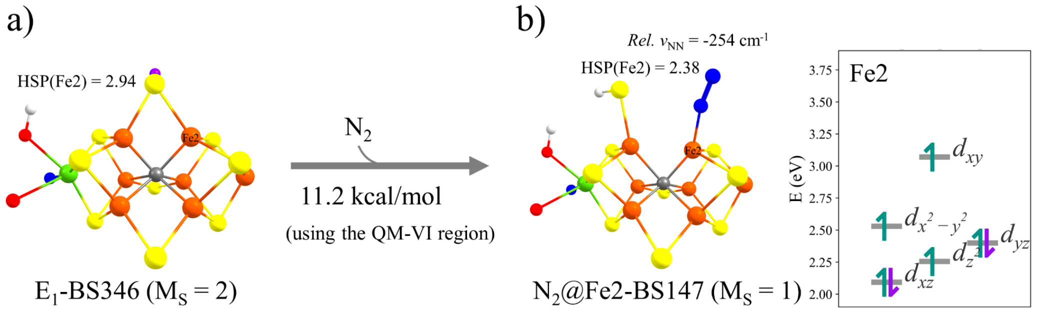

Figure S8. (a-b) The structure, relative N-N frequencies (relative to free N_2, $\nu_{NN,\ free}$ = 2432 cm^{-1}), QRO-based ligand-field diagrams of Fe2, and Hirshfeld spin populations (HSPs) of Fe2 of the most stable isomer for the $\mathbf{E_1}$ state before and after N_2 binding to Fe2, calculated using the QM-VI region. The binding energy in the middle refers to the left structure (a).

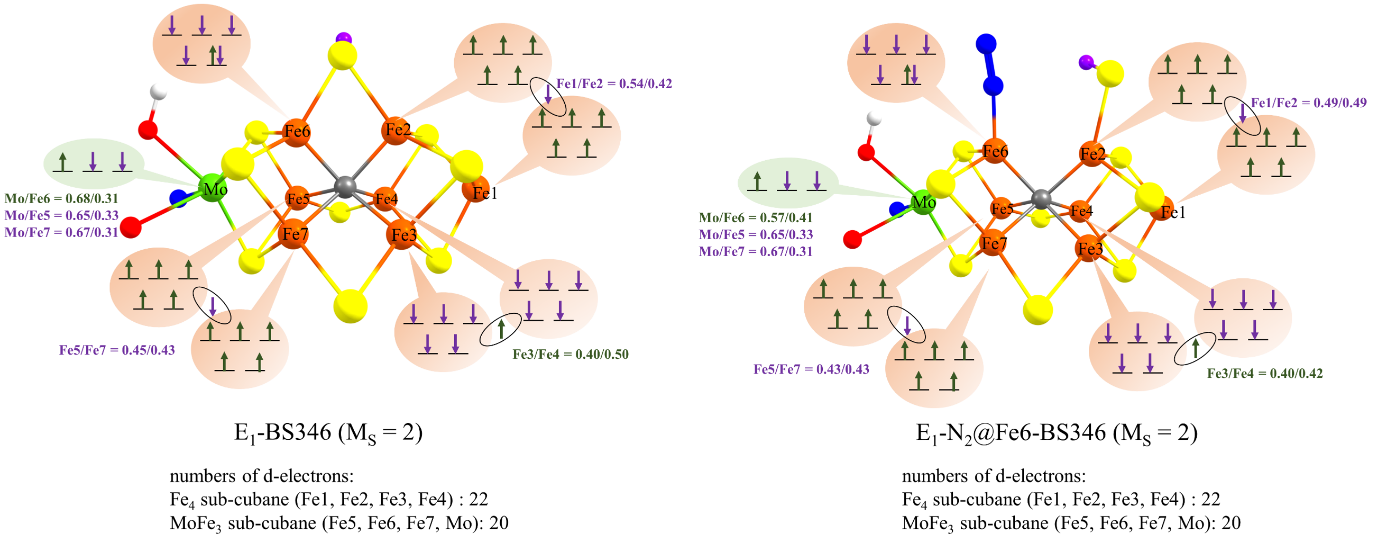

Figure S9. Electron configurations of FeMoco before and after N_2 binding in the $\mathbf{E_1}$ state: $\mathbf{E_1}$**-BS346 (**$\mathbf{M_S = 2}$**)** and $\mathbf{E_1}$**-**$\mathbf{N_2}$**@Fe6-BS346 (**$\mathbf{M_S = 2}$**)** according to localized orbital analysis using the QM-VI region. Localized orbital populations are shown for the Mo electron and Fe d-electrons with significant delocalization. Also shown are electron-counts per cubane.

7. CO binding to Fe6 of the E_1 state: binding energies

Figure S10 shows the binding energies of CO binding to Fe6 in the **E_1** state. The binding energy of -9.7 kcal/mol calculated using the QM-I region is reasonably exothermic, which is very different from the N_2 binding energy to the same state.

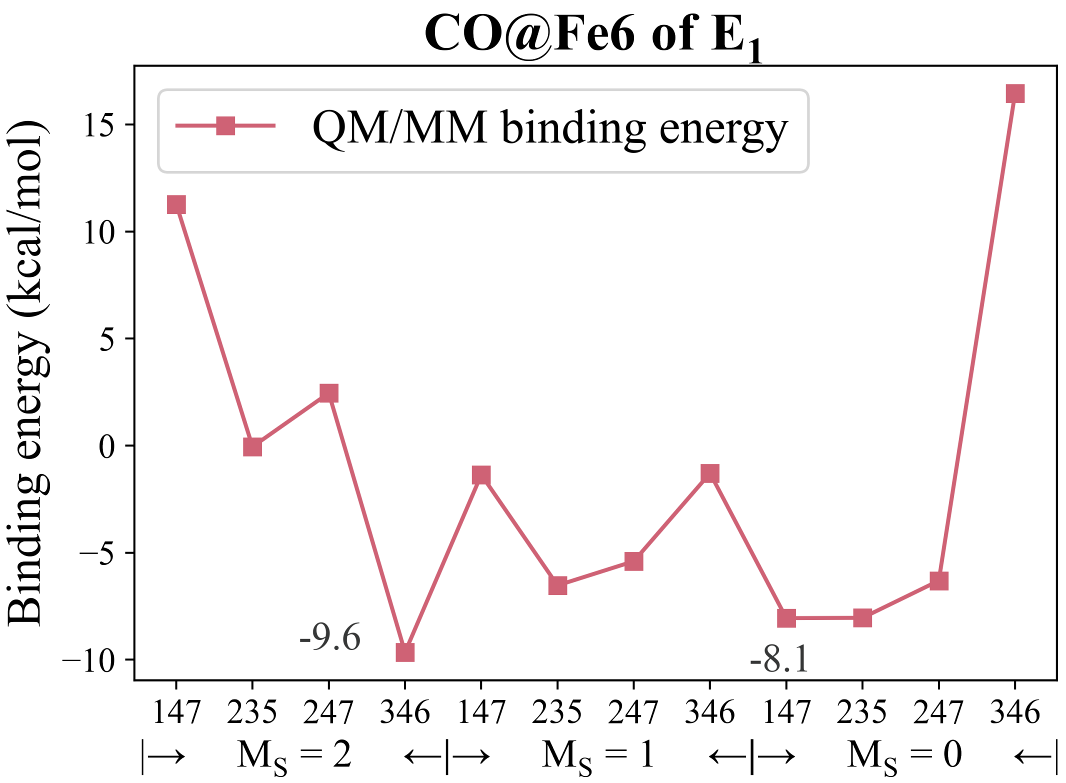

Figure S10. The plot shows CO binding energies to the Fe6 of the FeMoco in the **E_1** state calculated using the QM-I region. All binding energies are relative to **E_1-BS346 (M_S = 2)**.

8. N_2 binding to the E_2 state: structures, binding energies, QRO-based ligand-field diagrams, and electronic configurations based on the localized orbital analysis

Additional data on N_2 binding to the **E_2** state, presented in the 4th Results-Section of the manuscript. Figure S11 shows structures, relative frequencies, binding energies, QRO-based ligand-field diagrams, and Hirshfeld spin populations for Fe2 and Fe6 ions in the lowest-energy **E_2-nonhyd** isomer prior to and after N_2 binding to Fe2 and Fe6, respectively. While N_2 binding energies are similar for Fe2 and Fe6 positions, the structure of the protonated S2B differs in the N_2-bound forms (partially bridging in the N_2@Fe6 model while terminal in the N_2@Fe2 model). Furthermore, the local electronic structures of the Fe ions are different: being Fe^{2+} (S = 2) for the N_2@Fe6 model but Fe^{2+} (S = 1) in the N_2@Fe2 model.

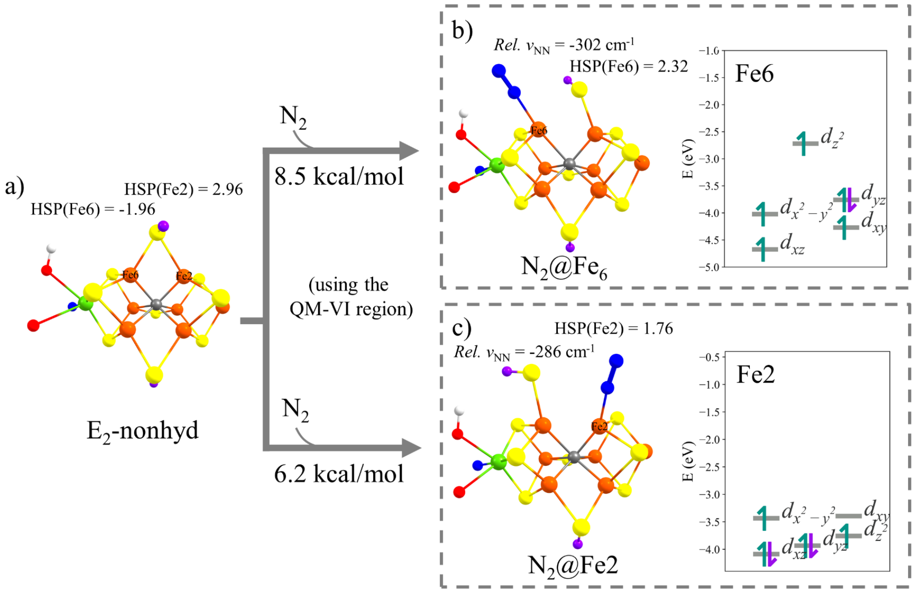

Figure S11. The structure, relative N-N frequencies (relative to free N_2, $\nu_{NN,\,free}$ = 2432 cm^{-1}), and QRO-based ligand-field diagrams and Hirshfeld spin populations (HSPs) of the indicated Fe ions for **E_2-nonhyd-BS346 (M_S = 3/2)** (a), **E_2-nonhyd-N_2@Fe6-BS235 (M_S = 3/2)** (b), and **E_2-nonhyd-N_2@Fe2-BS147 (M_S = 1/2)** (c).

Figure S12 shows the electron configurations based on the Pipek-Mezey localized orbital analysis of FeMoco in the **E_2-hyd** and **E_2-hyd-N_2@Fe6** states, which reveals that the 2 additional electrons in the **E_2-hyd** state (relative to **E_0**, see Figure S6) can approximately be considered to be located on the partially bridging hydride (with considerable contribution from the hydride binding Fe2 and Fe6 ions). The localized electronic structure of the **E_2-hyd-N_2@Fe6** model is more complicated, however, showing strong spin polarization of the hydride electrons and unusual electron configurations at Fe6 and Mo that could not be unambiguously assigned using the Pipek-Mezey localized orbital analysis.

Therefore, we tested two other localization methods (Foster-Boys[1] and IAO-IBO[2]) to get further insight into the electronic structure of **E_2-hyd-N_2@Fe6**, shown in Figure S13. The IAO-IBO localized orbitals resulted in almost identical assignment as Pipek-Mezey (not surprising as only the charge definition differs). Pipek-Mezey and IAO-IBO both suggest a S = 1/2 Fe^{3+} assignment on Fe6 although with an additional unusual delocalized electron between Mo and Fe6 and a beta-electron of the hydride surprisingly suggestive of being localized on Fe6. However, Foster-Boys suggests an Fe^{2+} low spin electron configurations state in which an additional electron transfers from Fe2. Based on results of these three localized methods and the Hirshfeld spin population (-0.08), both Fe^{2+} S = 0 and Fe^{3+} S = 1/2 assignments seem possible. Seeing as the Hirshfeld spin population is more consistent with a spin-paired electronic structure we tentatively assign Fe6 as a S = 0 Fe^{2+} ion.

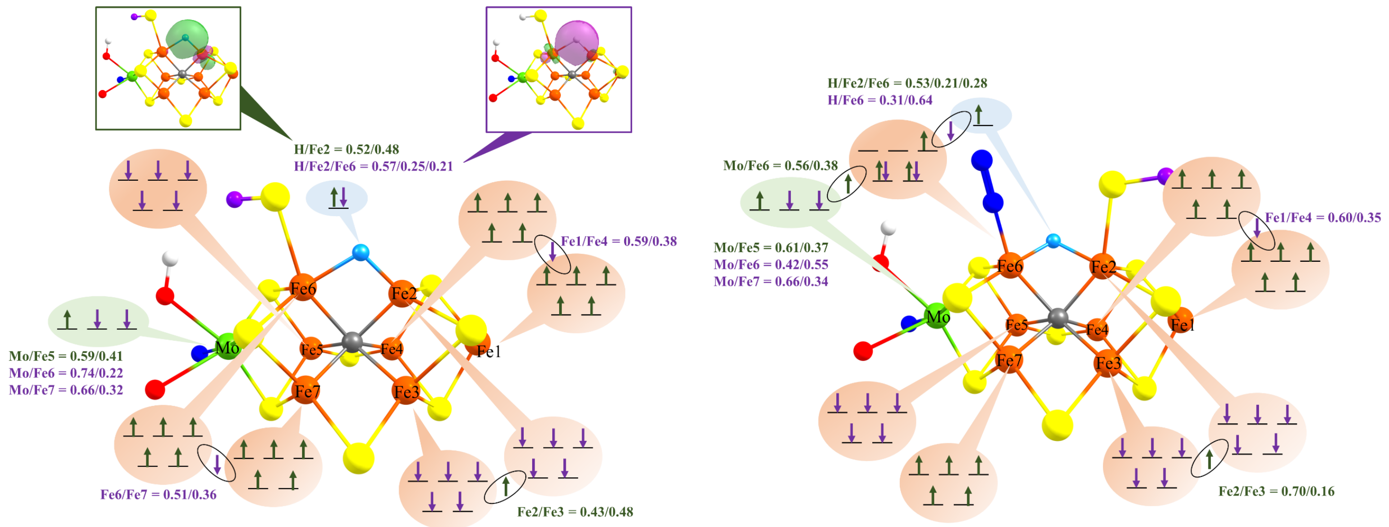

E_2-hyd-SH^-@Fe6-BS235 (M_S = 3/2)
numbers of d-electrons:
Fe_4 sub-cubane (Fe1, Fe2, Fe3, Fe4) : 22
$MoFe_3$ sub-cubane (Fe5, Fe6, Fe7, Mo): 19

E_2-hyd-N_2@Fe6-BS235 (M_S = 1/2)
numbers of d-electrons:
Fe_4 sub-cubane (Fe1, Fe2, Fe3, Fe4) : 22
$MoFe_3$ sub-cubane (Fe5, Fe6, Fe7, Mo): 20

Figure S12. Electron configurations of FeMoco before and after N_2 binding in the **E_2-hyd** state: **E_2-hyd-SH^-@Fe6-BS235 (M_S = 3/2)** and **E_2-hyd-N_2@Fe6-B235 (M_S = 1/2)** using the QM-VI region according to the localized method Pipek-Mezey. Localized orbital populations are shown for the Mo electrons and the Fe d-electrons with significant delocalization and the electrons associated with the hydride. Also shown are electron-counts per cubane.

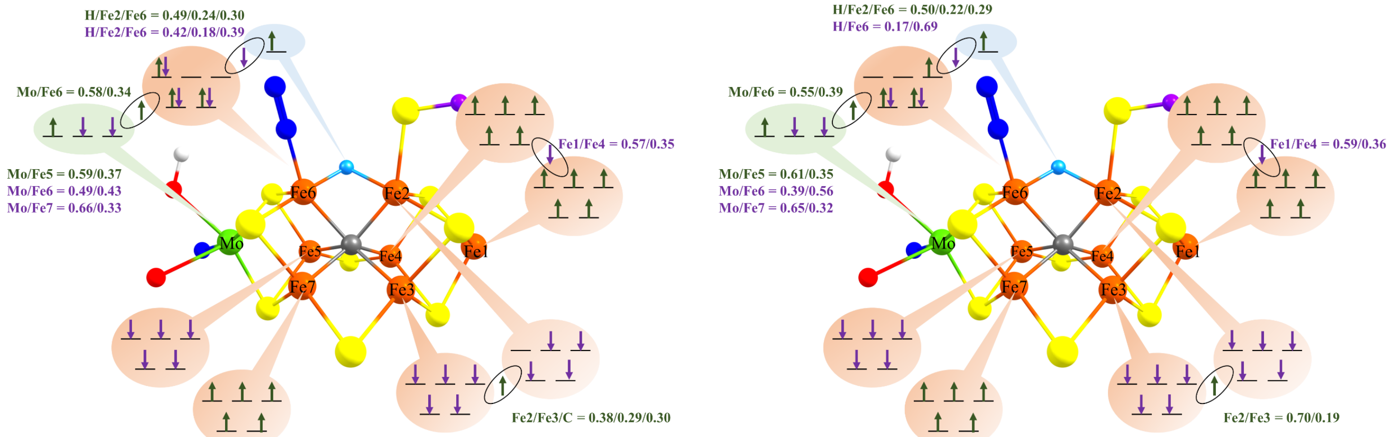

E_2-hyd-N_2@Fe6-BS235 (M_S = 1/2)
using Foster-Boys
numbers of d-electrons:
Fe_4 sub-cubane (Fe1, Fe2, Fe3, Fe4) : 21
$MoFe_3$ sub-cubane (Fe5, Fe6, Fe7, Mo): 20

E_2-hyd-N_2@Fe6-BS235 (M_S = 1/2)
using IAO-IBO
numbers of d-electrons:
Fe_4 sub-cubane (Fe1, Fe2, Fe3, Fe4) : 22
$MoFe_3$ sub-cubane (Fe5, Fe6, Fe7, Mo): 20

Figure S13. Electronic configurations of FeMoco for **E_2-hyd-N_2@Fe6-B235 (M_S = 1/2)** using two other localization methods (Foster-Boys and IAO-IBO) and the QM-VI region.

Figure S14 shows the electron configurations based on localized orbital analysis of FeMoco in the **E_2-nonhyd**, **E_2-nonhyd-N_2@Fe2**, and **E_2-nonhyd-N_2@Fe6** states. The 2 additional electrons end up reducing the Fe ions directly in the **E_2-nonhyd** state (relative to **E_0**, see Figure S6) Fe6 and Fe7 to ferrous states. The electronic structure of the **E_2-nonhyd-N_2@Fe2** model is more complicated due to the addition of the N_2 ligand and the BS147 configuration. Therefore, a straightforward interpretation is difficult. However, based on electron counts per cubane the $MoFe_3$ sub-cubane appears to have gained an electron (reducing Fe7 to a local Fe^{2+} state) while in the Fe_4 sub-cubane, Fe3 has become local Fe^{2+} while Fe2 is now in a local Fe^{2+} (S = 1) state. When N_2 binds to Fe6, the localized orbital analysis of the most stable BS235 M_S = 3/2 state, shown in Figure S14, reveals reduction of each $MoFe_3$ sub-cubane and Fe_4 sub-cubane. The Fe6 with N_2 bound is a high-spin Fe^{2+}.

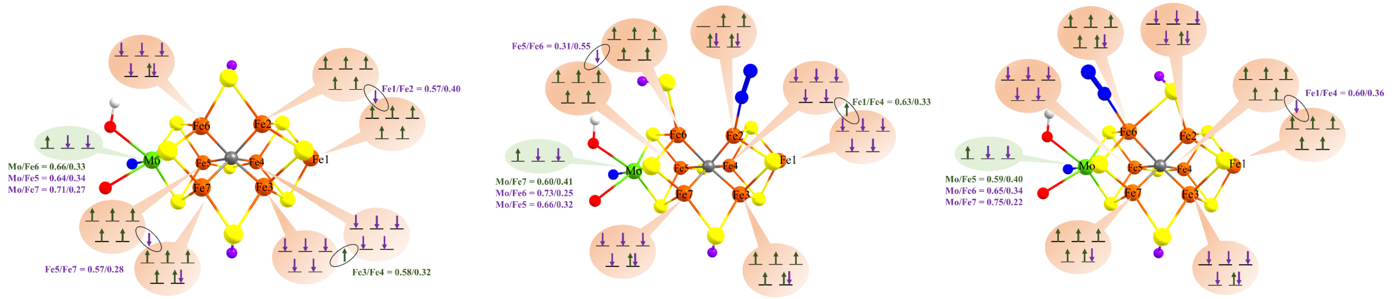

E_2-nonhyd-BS346 (M_S = 3/2)

numbers of d-electrons:
Fe_4 sub-cubane (Fe1, Fe2, Fe3, Fe4) : 22
$MoFe_3$ sub-cubane (Fe5, Fe6, Fe7, Mo): 21

E_2-nonhyd-N_2@Fe2-BS147 (M_S = 1/2)

numbers of d-electrons:
Fe_4 sub-cubane (Fe1, Fe2, Fe3, Fe4) : 23
$MoFe_3$ sub-cubane (Fe5, Fe6, Fe7, Mo): 20

E_2-nonhyd-N_2@Fe6-BS235 (M_S = 3/2)

numbers of d-electrons:
Fe_4 sub-cubane (Fe1, Fe2, Fe3, Fe4) : 23
$MoFe_3$ sub-cubane (Fe5, Fe6, Fe7, Mo): 20

Figure S14. Electron configurations of FeMoco before and after N_2 binding in the **E_2-nonhyd** state: **E_2-nonhyd-BS346 (M_S = 3/2), E_2-nonhyd-N_2@Fe2-B147 (M_S = 1/2)**, and **E_2-nonhyd-N_2@Fe6-B235 (M_S = 3/2)** according to localized orbital analysis using the QM-VI region. Localized orbital populations are shown for the Mo electrons as well as the Fe d-electrons with significant delocalization. Also shown are electron-counts per cubane.

9. N_2 binding energies, single-step binding energies, and relative N-N frequencies for E_0, E_0H^+, E_1, E_2, and E_4 states

Figure S15 shows the QM/MM binding energies of N_2 binding and relative N-N frequencies for nine models of **E_0**, **E_1**, **E_2**, and **E_4** states in multiple spin and BS states using the QM-I region (different from results using the QM-VI region). The binding energy decreases in going from **E_0** (**E_0H^+** being similar), **E_1**, **E_2-nonhyd**, **E_4-DP-Fe2/6(5)**, **E_4-DP-Fe2/6(3)**, **E_2-hyd**, and **E_4-SP** models. These binding energies can be divided into three groups of most favorable binding energies (for each redox state): endothermic binding energies higher than 8 kcal/mol, endothermic energies between 0 and 8 kcal/mol, and exothermic energies (< 0 kcal/mol). The first level includes **E_0** (similar to **E_0H^+**), **E_1**, **E_2-nonhyd**, FeMoco states all lacking a hydride. The second group consists of **E_4-DP-Fe2/6(3)**, **E_4-DP-Fe2/6(5)**, and **E_2-hyd** which are FeMoco states all containing a single hydride between Fe2 and Fe6 (for **E_4-DP-Fe2/6(3)**, **E_4-DP-Fe2/6(5)** the second hydride is located elsewhere). The third group contains the **E_4-SP** FeMoco isomers that contain two hydrides between Fe2 and Fe6. Binding energies are defined relative to the most stable isomer of each redox state (note that **E_2-nonhyd** is considered a separate redox state here).

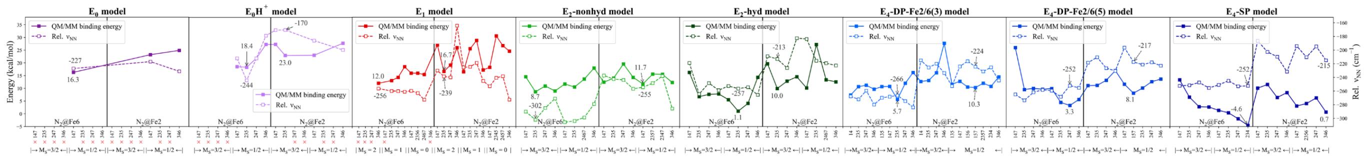

Figure S15. N_2 binding energies and relative N-N frequencies (related to free N_2, $\nu_{NN,\,free} = 2432\ cm^{-1}$) of **$E_0$**, **$E_0H^+$**, **$E_1$**, **$E_2$**, and **$E_4$** states calculated using the QM-I region. The graphs have a common y-axis scale (for both binding energies and rel. ν_{NN}). The N_2 binding energies of **E_0**, **E_0H^+**, **E_1**, and **E_2-nonhyd**, **E_2-hyd** are relative to states **E_0-BS235** (**$M_S = 3/2$**) (Figure S4a), **E_0H^+-BS235** (**$M_S = 3/2$**) (Figure 4), **E_1-BS346** (**$M_S = 2$**) (Figure S8a), **E_2-nonhyd-BS346** (**$M_S = 3/2$**) (Figure S11a), and **E_2-hyd-SH$^-$@Fe6-BS235** (**$M_S = 3/2$**) (the left structure of Figure 7a), respectively. The binding energy of **E_4-SP**, **E_4-DP-Fe2/6(3)**, and **E_4-DP-Fe2/6(5)** are relative to the same isomer **E_4-SP-SH$^-$@Fe6-BS346** with **$M_S = 3/2$** (Figure S17c).

Figure S16 shows the same QM/MM data as in Figure S15 but is instead plotted as single-step binding energies i.e. the energies of the N_2-bound states are relative to the FeMoco isomer that has a geometry closest to the N_2-binding isomer. This changes the energies for the states with bridging-hydrides and open sulfur bridges: **E_2-hyd** and **E_4** states.

The **E_0**, **E_0H^+**, **E_1**, and **E_2-nonhyd** states, have endothermic single-step binding energies more than 8 kcal/mol as before (unchanged). The most favorable single-step binding energies for **E_4-DP-Fe2/6(3)** and **E_4-DP-Fe2/6(5)** are -2.4 and -2.5 kcal/mol. The **E_2-hyd-N_2@Fe6** states change considerably revealing a considerably changed binding affinity to this site (-11.6 kcal/mol). This large difference between the single-step binding energy and the binding energy arises because the **E_2-hyd** state with an open coordination site at Fe6 (**E_2-hyd-SH$^-$@Fe2**) is quite unfavorable.

Similarly, for the most favorable single-step binding energy of -16.1 kcal/mol for the **E_4-SP-N_2@Fe6** model (revealing strong binding affinity of the Fe6 site) the difference arises due to the fact that the **E_4-SP** model with an open coordination site at Fe6 (**E_4-SP-SH$^-$@Fe2**) is actually rather unstable compared to the lowest energy one (**E_4-SP-SH$^-$@Fe6**).

The major trends are left unchanged: the endothermic **E_0**, **E_0H^+**, **E_1**, and **E_2-nonhyd** states have no hydride present, the mildly exothermic **E_4-DP-Fe2/6(3)** and **E_4-DP-Fe2/6(5)** and **E_2-hyd** have a single hydride present between Fe2 and Fe6. Finally, the most exothermic **E_4-SP** has two hydrides bridging Fe2 and Fe6.

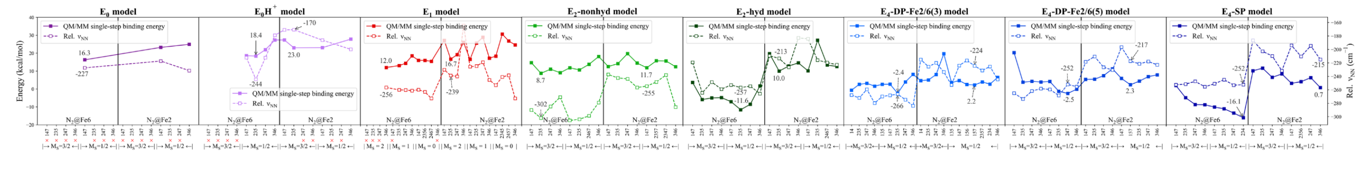

Figure S16. N_2 single-step binding energies and relative N-N frequencies (related to free N_2, $\nu_{NN,\,free} = 2432\ cm^{-1}$) of **$E_0$**, **$E_0H^+$**, **$E_1$**, **$E_2$**, and **$E_4$** states using the QM-I region. These plots have the same y-axis region, which helps reveal the trends between different **E_n** states. The single-step binding energies of **E_0**, **E_0H^+**, **E_1**, **E_2-nonhyd**, **E_2-hyd-N_2@Fe6**, **E_2-hyd-N_2@Fe2**, **E_4-SP-N_2@Fe6**, **E_4-SP-N_2@Fe2**, **E_4-DP-Fe2/6(3)**, and **E_4-DP-Fe2/6(5)** are relative to states **E_0-BS235** (**$M_S = 3/2$**) (Figure S4a), **E_0H^+-BS235** (**$M_S = 3/2$**) (Figure 4), **E_1-BS346** (**$M_S = 2$**) (Figure S8a), **E_2-nonhyd-BS346** (**$M_S = 3/2$**) (Figure S11a), **E_2-hyd-SH$^-$@Fe2-BS235** (**$M_S = 3/2$**) (the right structure of Figure 7a), **E_2-hyd-SH$^-$@Fe6** (**$M_S = 3/2$**) (the left structure of Figure 7a), **E_4-SP-SH$^-$@Fe2-BS147 ($M_S = 1/2$)** (Figure S17e), **E_4-SP-SH$^-$@Fe6-BS346 ($M_S = 3/2$)** (Figure S17c), **E_4-DP-Fe2/6(3)-BS135 ($M_S = 1/2$)** (Figure S17a), **E_4-DP-Fe2/6(5)-BS147 ($M_S = 1/2$)** (Figure S17b), respectively.

A clear difference between the binding affinity of Fe2 and Fe6 sites is revealed from the plots. This is in large part due to the presence of the His195 residue close to the Fe2 site. Additionally, while N_2 binding energies correlate reasonably well with N_2 activation (as seen via the shift in N_2 vib. frequency) for most states, the **E_2-nonhyd** states are the exception, revealing N_2-bound states with the most activated N_2 ligands despite unfavorable N_2 binding energies.

10. N_2 binding to the E_4 state: structures, N_2 binding energies, single-step N_2 binding energies, QRO-based ligand-field diagrams, and electronic configurations from localized orbital analysis

Figure S17 shows relative QM/MM energies and relevant QRO-based ligand-field diagrams for three types of **E_4** states before N_2 binding, calculated using r^{2}SCAN and the QM-VI region. Also shown is a plot associated with relative QM/MM energies using four functionals, B97-D3, r^{2}SCAN, TPSSh, B3LYP*, which were the best performing functionals in a geometric benchmarking study.[3] The plot indicates a strong thermodynamic preference for an open belt sulfur bridge (FeMoco isomers **E_4-SP** shown in c and d) with bridging hydrides between Fe2 and Fe6, regardless of functional. **E_4-SP-SH$^-$@Fe6-BS346 (M_S = 3/2)** has a spin state inconsistent with the EPR S = 1/2 signal (and lacks 2 bridging hydrides as ENDOR indicates), however, it is only slightly lower in energy than the **E_4-SP-SH$^-$@Fe6-BS2467 (M_S = 1/2)** state with 2 bridging hydrides.

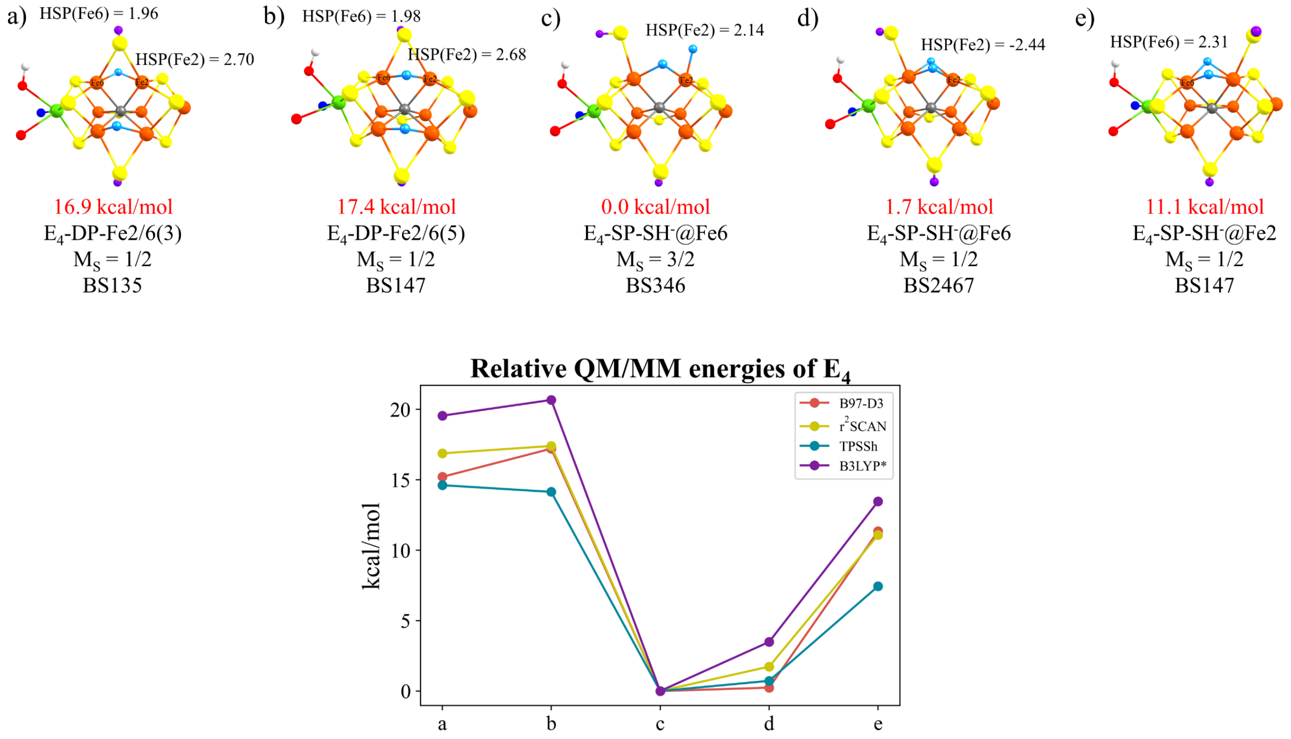

Figure S17. (a-e) Structures, and Hirshfeld spin populations (HSPs) of labeled Fe for three types of **E_4** states before N_2 binding calculated using r^{2}SCAN and the QM-VI region. Also shown are relative QM/MM energies of three types of the **E_4** state using four different functionals.

Figure S18 shows relative QM/MM energies, N_2 binding energies, single-step binding energies, and QRO-based ligand-field diagrams of indicated Fe ions of **E_4-DP-Fe2/6(3)** and **E_4-DP-Fe2/6(5)** models. When N_2 binds to Fe2 or Fe6 of these two models, there are similar binding energies and single-step binding energies, which are much higher than the binding energies and single-step binding energies of the **E_4-SP** model.

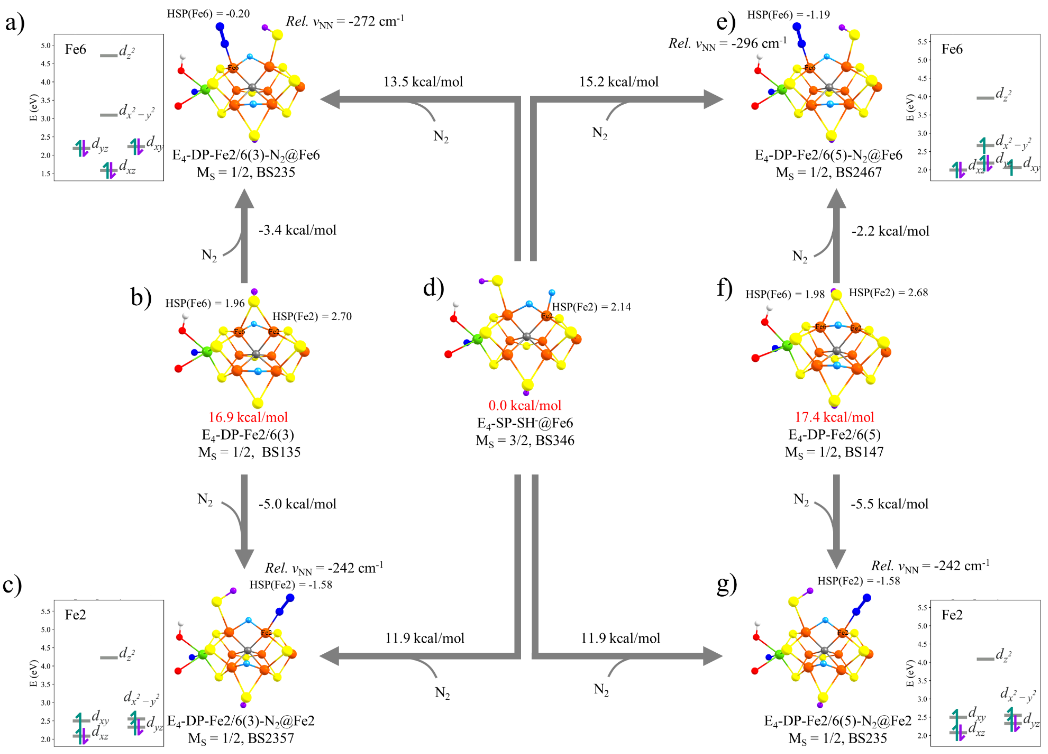

Figure S18. Structures, QRO-based ligand-field diagrams and Hirshfeld spin populations (HSPs) of labeled Fe ions, relative energies, N_2 binding energies, and single-step binding energies for **E$_4$-DP-Fe2/6(3)** and **E$_4$-DP-Fe2/6(5)** models using the QM-VI region. All binding energies are relative to the lowest **E$_4$** isomer (d). The single-step binding energies (-5.0, -5.5, -3.4, and -2.2 kcal/mol) of N_2 binding to **E$_4$-DP-Fe2/6(3)** and **E$_4$-DP-Fe2/6(5)** models are relative to **E$_4$-DP-Fe2/6(3)-BS135** with M_S = 1/2 (b) and **E$_4$-DP-Fe2/6(5)-BS147** with M_S = 1/2 (f), respectively.

Figure S19 and Figure S20 show electronic configurations of **E$_4$-SP** models before and after N_2 binding to Fe2 or Fe6 according to the localized orbital analysis.

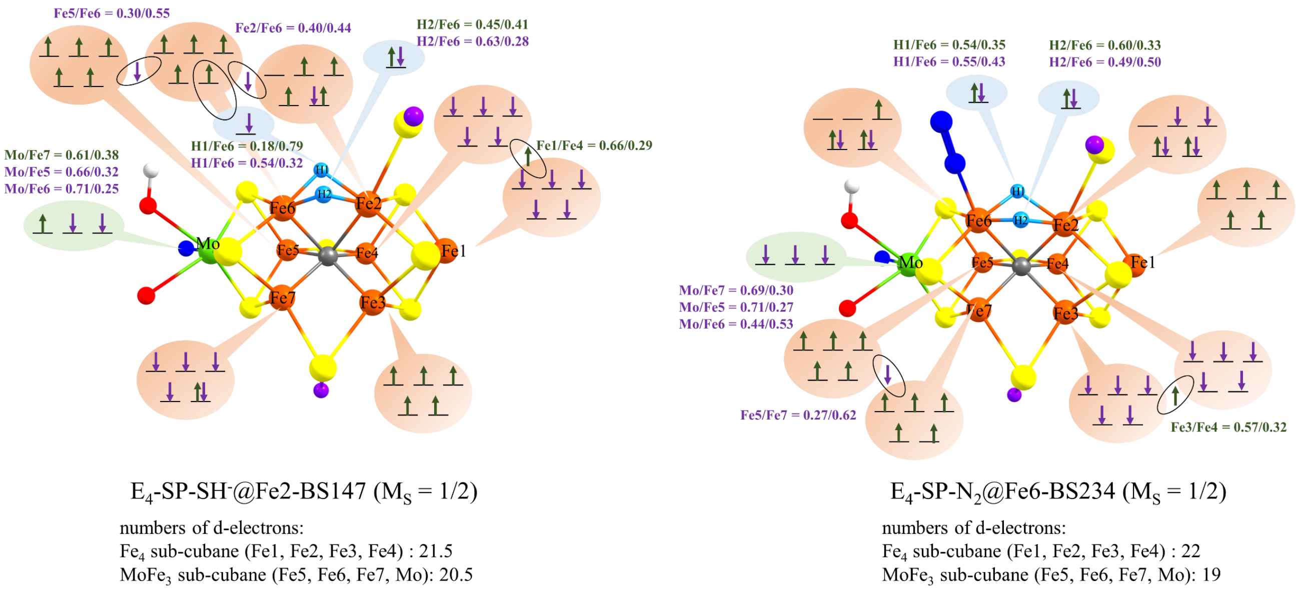

Figure S19. Electronic configurations based on localized orbital analysis for **E$_4$-SP** models before and after N_2 binding to Fe6 using the QM-VI region. Localized orbital populations are shown for the Mo electrons as well as the Fe d-electrons with significant delocalization. Also shown are electron-counts per cubane.

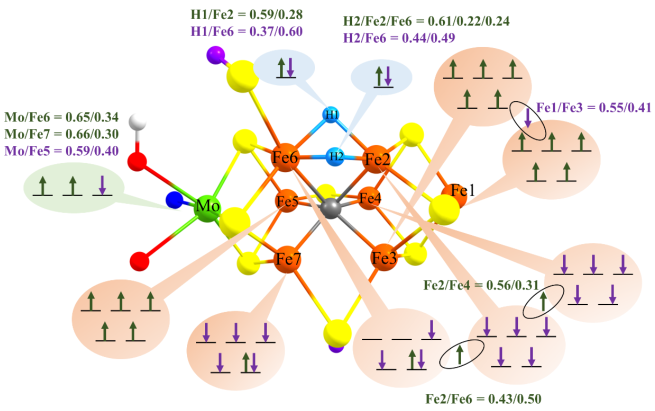

E_4-SP-SH^-@Fe6-BS247 (M_S = 1/2)

numbers of d-electrons:
Fe_4 sub-cubane (Fe1, Fe2, Fe3, Fe4) : 22.5
$MoFe_3$ sub-cubane (Fe5, Fe6, Fe7, Mo): 18.5

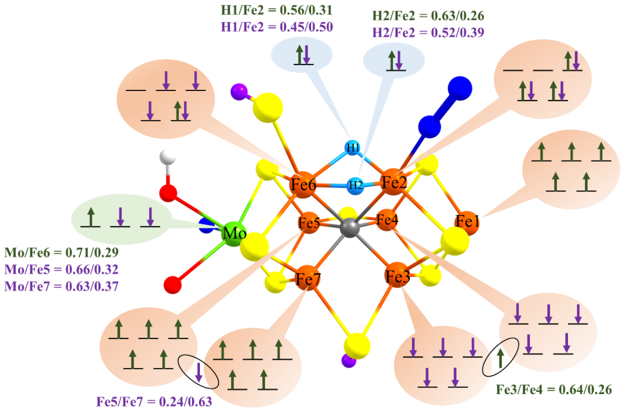

E_4-SP-N_2@Fe2-BS346 (M_S = 1/2)

numbers of d-electrons:
Fe_4 sub-cubane (Fe1, Fe2, Fe3, Fe4) : 22
$MoFe_3$ sub-cubane (Fe5, Fe6, Fe7, Mo): 19

Figure S20. Electronic configurations based on localized orbital analysis for **E_4-SP** models before and after N_2 binding to Fe2 using the QM-VI region. Localized orbital populations are shown for the Mo electrons as well as the Fe d-electrons with significant delocalization. Also shown are electron-counts per cubane.

11. Testing additional BS solutions for E_4 models

The spin-coupling problem of FeMoco has previously been discussed in terms of spin-flipping 3 Fe ions which gives rise to 35 BS solutions. The Mo ion is here considered not to be part of the spin-coupling problem due to its unusual non-Hund configuration. The relative energies of the 35 BS solutions of FeMoco in the $\mathbf{E_0}$ state have been previously discussed.[4]

However, when N_2 binds to either Fe2 or Fe6 of **E_4-SP** isomers, one of these Fe ions tends to result in low local spin states (S = 0, or S = 1/2) than the typical high-spin states of S = 5/2 (for Fe^{3+}) and S = 2 (for Fe^{2+}) for the regular weak-field ligand environment of FeMoco. This changes the nature of the spin-coupling considerably and thus we test additional BS solutions here to test if more stable solutions can be found. If, to an approximation, one of the Fe ions is considered no longer to be part of the spin-state problem (S = 0, S = 1/2), 35 BS state possibilities become 20 (= $\frac{6!}{(6-3)!\times 3!}$) possibilities where 3 Fe ions are spin-flipped. This was systematically explored below (Figure S21) for the **E_4-SP** with N_2 binding in this work.

Overall the results reveal that the most stable BS solution for **E_4-SP-N_2@Fe2** and **E_4-SP-N_2@Fe6** are BS346 and BS234, respectively, which are converged from the 4 BS solutions originally tested (BS234 converged from the calculation of BS346).

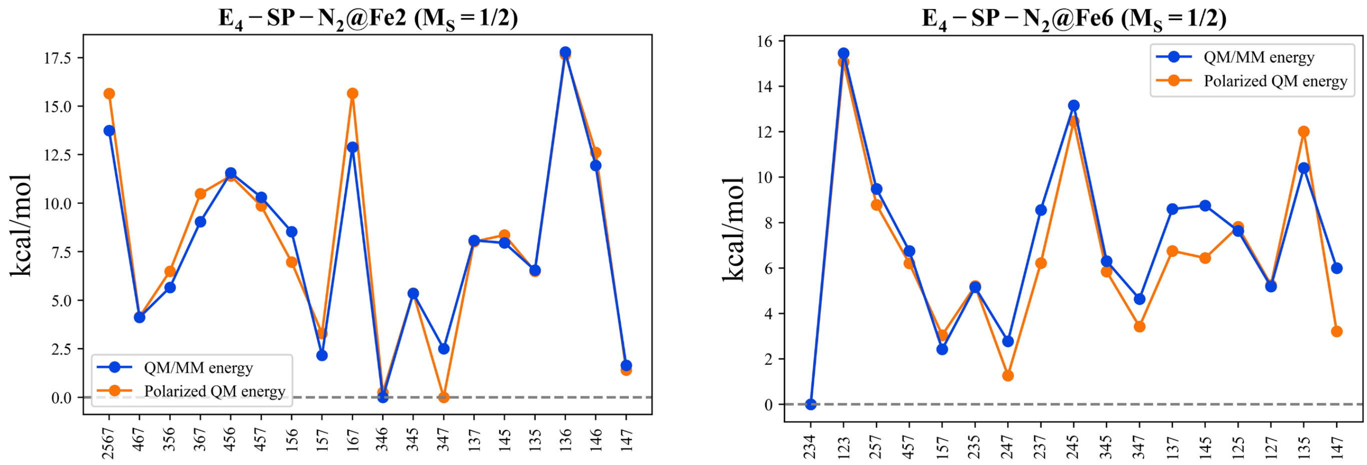

Figure S21. Twenty BS solutions were investigated for N_2 binding to Fe2 or Fe6 of **E_4-SP** models using the QM-I region. When N_2 binds to the FeMoco of **E_4-SP** models, the binding site Fe2 or Fe6 tends to be low-spin Fe^{3+} or Fe^{2+}, which differs from $\mathbf{E_0}$. There are 20 BS solutions when ignoring the spin state of binding site Fe among 35 BS solutions. **E_4-SP-N_2@Fe2-BS346** with M_S = 1/2 and **E_4-SP-N_2@Fe6-BS234** with M_S = 1/2 are the lowest-energy isomers when N_2 binding to Fe2 and Fe6, respectively.

Cao and Ryde previously tested 56 (= 35 + 21) BS solutions for **E_4-DP-Fe2/6(3)** and **E_4-DP-Fe2/6(5)** models but using a different QM/MM setup and different functional and basis sets.[5] The 21 (=$\frac{7!}{(7-2)!\times 2!}$) solutions have 2 down-spin irons rather than 3. In this work, these 56 BS solutions were also systematically studied for **E_4-DP-Fe2/6(3)** and **E_4-DP-Fe2/6(5)** models without N_2 binding shown in Figure S22. When we tried those 21 BS solutions with 2 down-spin irons, most of BS solutions converged to solutions with 3 or even 4 down-spin irons, as shown in Figure S22. We only found BS13 and BS14 for the **E_4-DP-Fe2/6(3)** and BS14 for the **E_4-DP-Fe2/6(5)**, which means that **E_4-DP-Fe2/6(3)** and **E_4-DP-Fe2/6(5)** models tend to have more than two spin-down Fe ions. Therefore, we only studied 35 BS solutions with 3 down-spin irons for **E_4-DP-Fe2/6(3)** and **E_4-DP-Fe2/6(5)** models after N_2 binding shown in Figure S22. The lowest-energy BS solution for N_2 binding to Fe6 of both **E_4-DP-Fe2/6(3)** and **E_4-DP-Fe2/6(5)** is one of 4 BS solutions tested initially. However, when N_2 binds to Fe2, the lowest-energy BS solution becomes BS156 or BS157 for **E_4-DP-Fe2/6(3)** and becomes BS157 for **E_4-DP-Fe2/6(5)**. For these three lower energy isomers, we expanded the QM-I region to QM-VI region, however, they were found to be less stable than the lowest-energy isomer of the initial 4 BS solutions shown in Table S17. The energies of BS156 and BS157 of **E_4-DP-Fe2/6(3)** are higher than BS2357 (converged from BS235), and BS157 of **E_4-DP-Fe2/6(5)** is also higher than BS235 in energy.

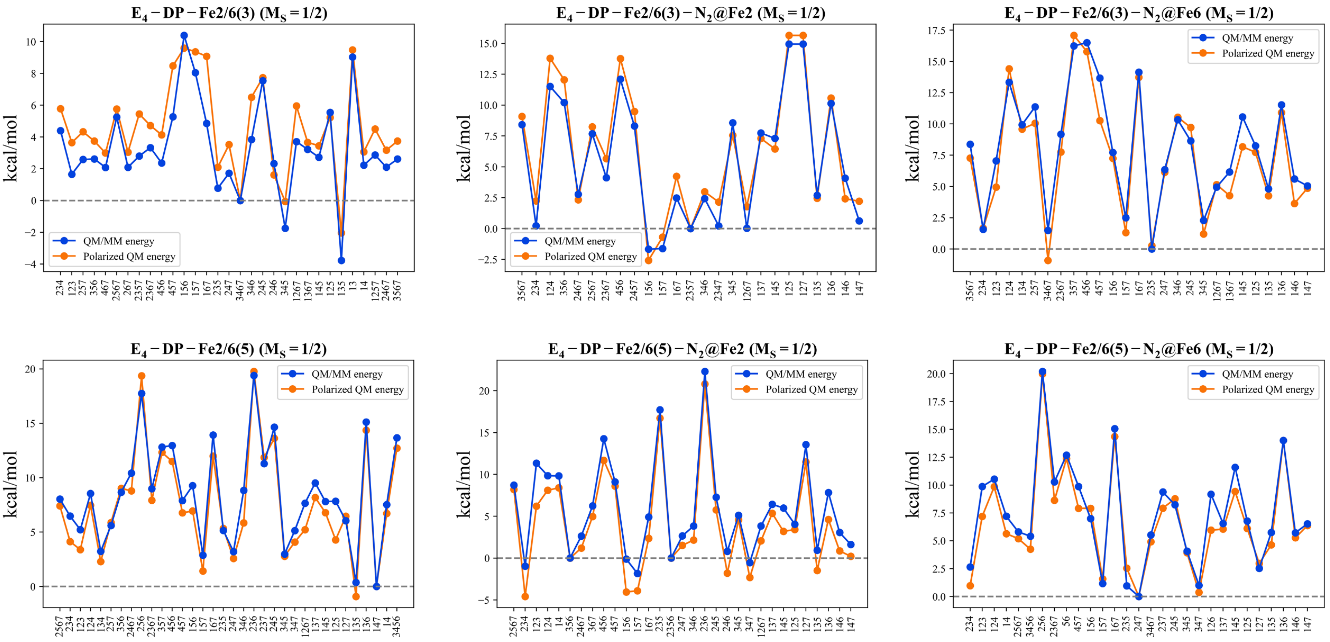

Figure S22. The left two plots show relative energies among 56 BS solutions (35 solutions with three down-spin irons and 21 solutions with two down-spin irons) for **E_4-DP-Fe2/6(3)** and **E_4-DP-Fe2/6(5)** models without N_2 binding using the QM-I region. We found that these two **E_4** models converged to solutions with more than two spin-down Fe ions. Therefore, trying 35 BS solutions for these two **E_4** models with N_2 binding is reasonable, as shown in the middle and right.

12. The Hirshfeld charges and populations of distal N and Fe2 or Fe6 when N_2 binding

Figure S23 shows the correlation of single-step N_2 binding energies and Hirshfeld charges or spin populations of Fe ions bound N_2 and distal N.

The charges and spin populations of Fe6 of **E_2-hyd** and **E_4-SP** models are either more negative or smaller than **E_0** and **E_1** models, suggesting that Fe6 becomes more electron-rich and lower-spin when the **E_n** state changes from **E_1** to **E_2-hyd**. The reason is likely to be the presence of the hydride. The trend of charges of distal N-atom of the N_2 ligand is similar to that of the single-step binding energies, showing that slightly more electron density is pushed to N_2 with increasing **E_n** states.

When N_2 binds to Fe2, the **E_4-SP** model has the lowest single-step binding energy, the most negative Fe2, the lowest spin state, and the most negative distal N. However, single-step binding energies otherwise do not correlate with the charges of Fe2 and distal N, and the spin population of Fe2.

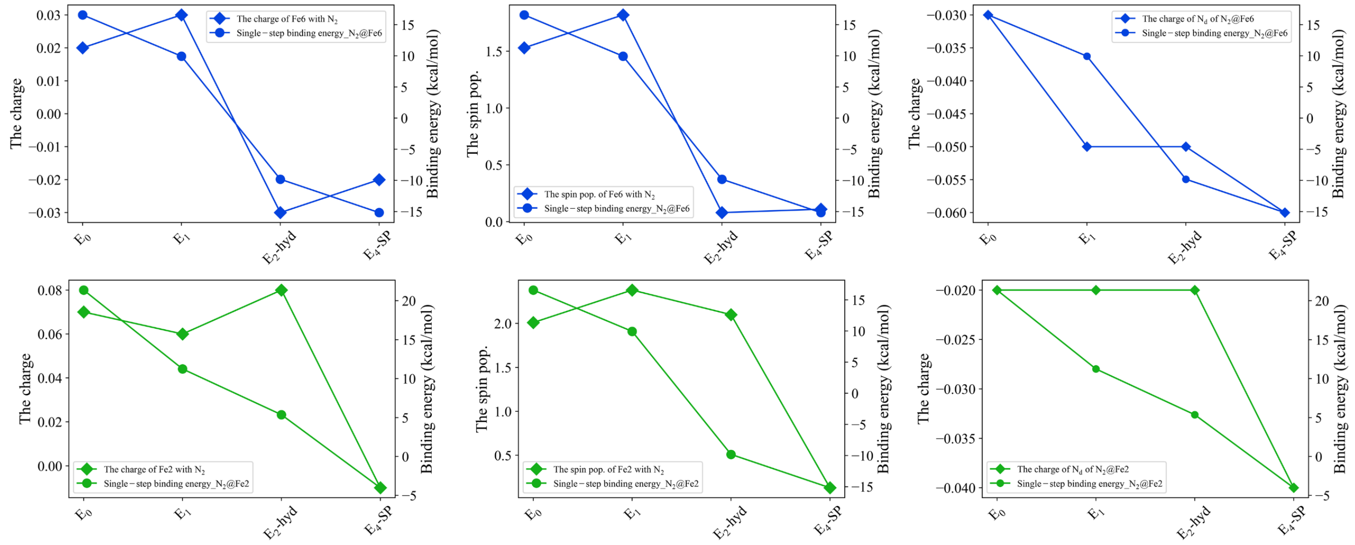

Figure S23. These plots show the correlation of single-step N_2 binding energies and Hirshfeld charges or populations of binding site Fe and distal N for the most stable isomer bound N_2 of each **E_n** state using the QM-VI region.

Table S4. The Hirshfeld charges and populations of Fe6 and distal N for the most stable N_2 bound isomer of each **E_n** state using the QM-VI region.

	**The charge of Fe6**	**The spin population of Fe6**	**The charge of distal N**
E_0-N_2@Fe6-BS147 (M_S = 1/2)	0.02	1.53	-0.03
E_1-N_2@Fe6-BS346 (M_S = 2)	0.03	-1.82	-0.05
E_2-hyd-N_2@Fe6-BS235 (M_S = 1/2)	-0.03	-0.08	-0.05
E_4-SP-N_2@Fe6-BS234 (M_S = 1/2)	-0.02	-0.11	-0.06

Table S5. The Hirshfeld charges and populations of Fe2 and distal N for the most stable N_2 bound isomer of each **E_n** state using the QM-VI region.

	**The charge of Fe2**	**The spin population of Fe2**	**The charge of distal N**
E_0-N_2@Fe2-BS147 (M_S = 1/2)	0.07	2.01	-0.02
E_1-N_2@Fe2-BS147 (M_S = 1)	0.06	2.38	-0.02
E_2-hyd-N_2@Fe2-BS235 (M_S = 3/2)	0.08	-2.10	-0.02
E_4-SP-N_2@Fe2-BS346 (M_S = 1/2)	-0.01	-0.13	-0.04

13. The N_2 binding affinities from E_0 to E_4 states using cluster models

Figure S24 shows that the QM/MM (with a large QM-region, QM-VI) and cluster-continuum models (with a cofactor-only cluster same as the QM-I region) show the same trend of single-step N_2 binding energies from $\mathbf{E_0}$ to $\mathbf{E_4}$ states, although the absolute values depend quite a bit on the quality of the model and environment. The single-step N_2 binding energies become more and more favorable from $\mathbf{E_0}$, $\mathbf{E_1}$, $\mathbf{E_2}$ to $\mathbf{E_4}$ states, further supporting our hypothesis that the $\mathbf{E_4}$ redox state with 2 bridging hydrides between Fe2 and Fe6 is a favourable model for binding N_2. All single-step N_2 binding energies are relative to the $\mathbf{E_n}$ state (without N_2) that is geometrically closest to the N_2-bound geometry (which is not necessarily the most favorable $\mathbf{E_n}$ isomer).

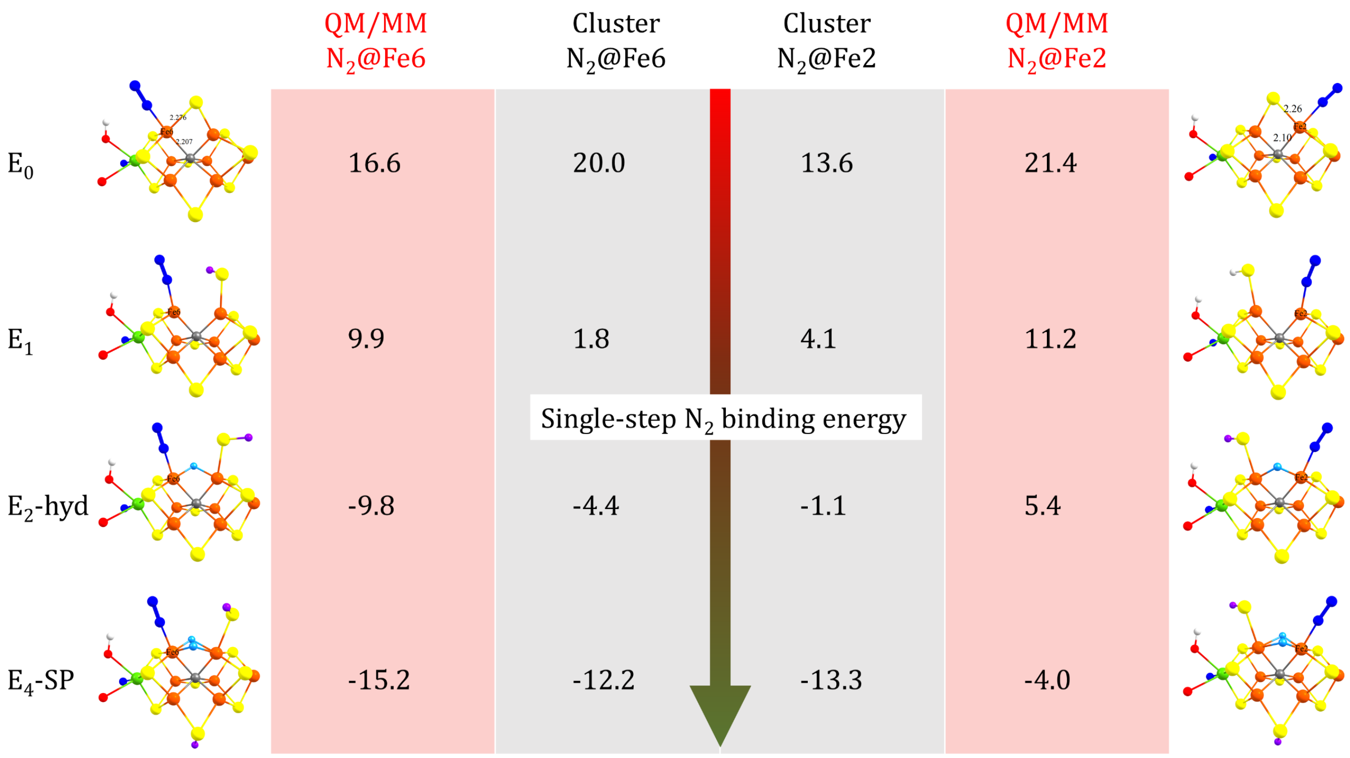

Figure S24. Comparison of single-step N_2 binding energies to $\mathbf{E_n}$ states of FeMoco between QM/MM and QM-I-cluster-continuum computational setups. The detail information for states in the figure: **E_0-N_2@Fe6-BS147 (M_S = 1/2)**, **E_1-N_2@Fe6-BS346 (M_S = 2)**, **E_2-hyd-N_2@Fe6-BS235 (M_S = 1/2)**, **E_4-SP-N_2@Fe6-BS234 (M_S = 1/2)**, **E_0-N_2@Fe2-BS147 (M_S = 1/2)**, **E_1-N_2@Fe2-BS147 (M_S = 1)**, **E_2-hyd-N_2@Fe2-BS235 (M_S = 3/2)**, **E_4-SP-N_2@Fe2-BS346 (M_S = 1/2)**. The QM/MM and cluster models use the QM-VI and QM-I regions, respectively.

14. The effect of different His195 protonation states for N_2 binding to the E_4-SP model

The section "N_2 Binding to E_4-SP in Two α-His195 Protonation States" in the manuscript compares the effect of the His195 protonation state: His195-$N_δ$(H) vs. His195-$N_ε$(H) on N_2 binding energies. We here show the comparison of QM/MM energies between His195-$N_δ$(H) and His195-$N_ε$(H) first as well as attempts to describe His195 as doubly protonated.

Table S6 shows that His195-$N_ε$(H) is lower than His195-$N_δ$(H) by more than 15 kcal/mol. The X-ray structure reveals hydrogen bonding distances between S2B and $N_ε$, suggesting His195-$N_ε$(H) in the $\mathbf{E_0}$ state. There is additionally a hydrogen bond between a water molecule and $N_δ$, which in our model is modelled as the water molecule being the donor, and additional hydrogen bonding to Tyr281 and other nearby residues is also present. This specific hydrogen-bonding pattern is likely behind the His195-$N_ε$(H) preference. As our QM/MM model is based on the $\mathbf{E_0}$ model (based on the X-ray structure), it is not surprising that His195-$N_ε$(H) thus remains more stable than His195-$N_δ$(H) in the $\mathbf{E_4}$ state. However, as X-ray structures are not available for reduced $\mathbf{E_n}$ states, it is not known whether hydrogen bonding network changes might have occured which conceivably may alter this preference. In this study, we focus on the relative energies and binding energies for each protonation states, therefore, we do not compare energies between different protonation states when discussing relative energies between **E_4-SP-SH^-@Fe6** and **E_4-SP-SH^-@Fe2** and binding energies, and we regard the **E_4-SP-SH^-@Fe6** as the 0.0 kcal/mol for each protonation states.

Table S6. Energy differences between His195-$N_δ$(H) and His195-$N_ε$(H) using the QM-VI region. Polarized QM energies, MM energies and total QM/MM energies are shown.

	△E(polarized QM)	△E(MM)	△E(QM/MM)
E_4-SP-SH^-@Fe6-$N_ε$(H)	0.0	0.0	0.0
E_4-SP-SH^-@Fe6-$N_δ$(H)	17.5	4.8	22.3
E_4-SP-SH^-@Fe2-$N_ε$(H)	0.0	0.0	0.0
E_4-SP-SH^-@Fe2-$N_δ$(H)	16.2	1.8	18.0
E_4-SP-N_2@Fe6-$N_ε$(H)	0.0	0.0	0.0
E_4-SP-N_2@Fe6-$N_δ$(H)	14.3	1.7	16.0
E_4-SP-N_2@Fe2-$N_ε$(H)	0.0	0.0	0.0
E_4-SP-N_2@Fe2-$N_δ$(H)	14.4	4.2	18.6

Figure S25 shows the N_2 binding energies and single-step binding energies for the **E_4-SP** model with an initial doubly protonated α-His195, named His195-$N_{δ,ε}$(H). Calculations on this state, however, are highly problematic as spontaneous proton transfer is observed for most of these states, which is different from previous Dance's research[6] (found stable minima containing a doubly protonated His195). Prior to N_2 binding, the proton of $N_ε$ is found to transfer to the terminal SH^- group, forming H_2S when the SH^- is on Fe2. However, when the SH^- group is instead on Fe6, i.e. farther from His195, the proton of $N_ε$ instead jumps over to combine with a hydride and forms a Fe-bound H_2. These results thus indicate that doubly protonated His195 is extremely acidic and reactive and it's unclear whether such a state would ever form during realistic nitrogenase conditions.

When N_2 is bound to Fe2 the proton transfer does not occur, while when N_2 is bound to Fe6 proton-transfer is observed to form an H_2S group. These proton-transfer prevents us from giving a useful description of the effect of doubly protonated His195 on N_2 binding. We do note, however, that the results in Figure S25 suggest that if doubly protonated His195 could form, N_2 binding at Fe2 would be unlikely to occur while N_2 binding at Fe6 might still occur, with a conceivable spontaneous protonation of the SH^- group to Fe-bound H_2S.

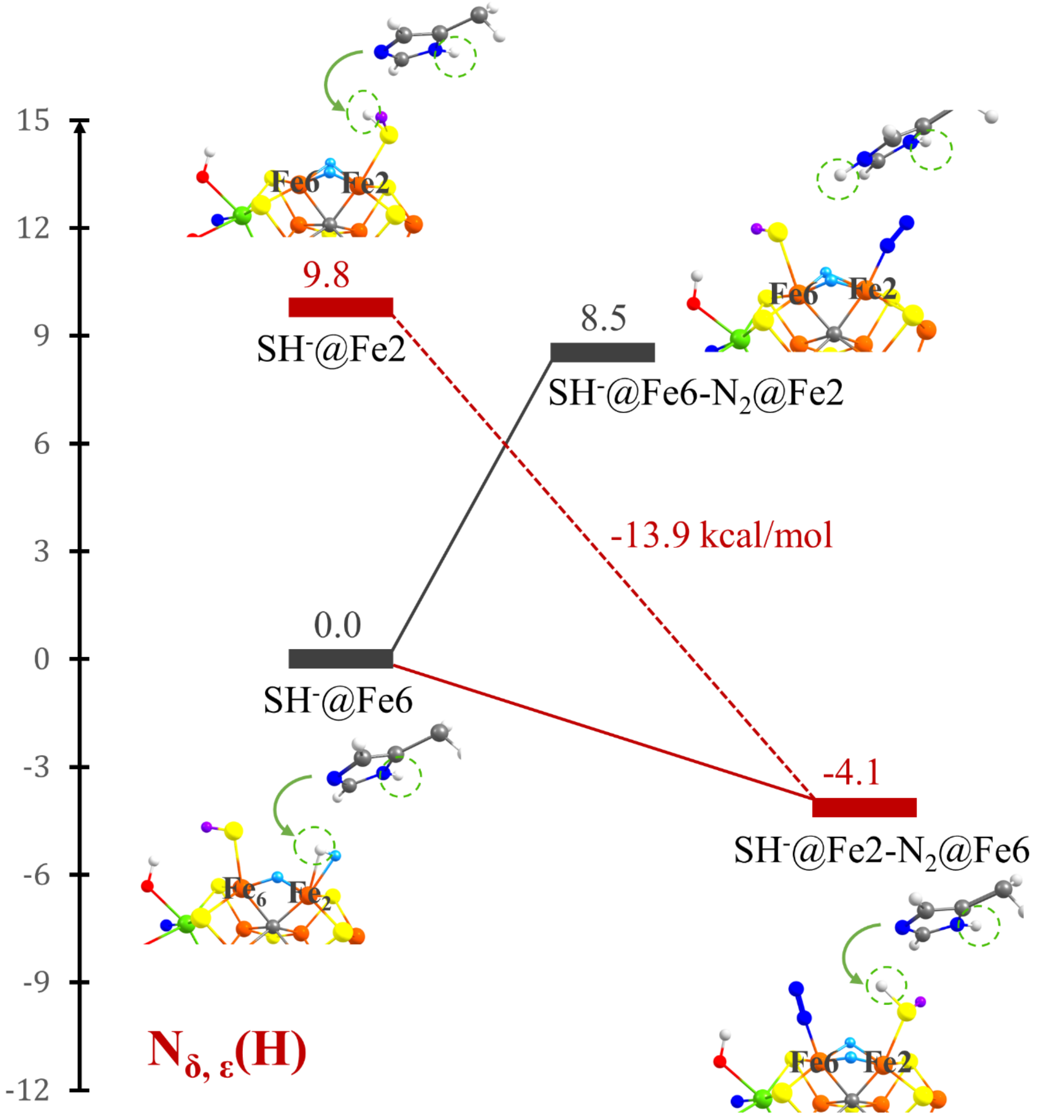

Figure S25. The N_2 binding energies in the **E_4-SP** state with a doubly protonated α-His195 using the QM-VI region.

15. How the QRO-based ligand-field diagrams were created

Getting insight into the local electronic structure of individual metal ions of FeMoco is not straightforward. While the localized orbital analysis is extremely useful in deriving approximate electronic configurations, unfortunately there are no well-defined energies associated with localized orbitals which prevent one from discussing the local electronic structure in terms of MO diagrams and ligand-field gaps etc. Diamagnetic substitution was utilized to derive such approximate ligand-field diagrams for a single open-shell Fe ion (as described in section 3 of the manuscript).

Using Fe6 of the **E_4-SP-N_2@Fe6-BS234** with M_S = 1/2 model as an example, the QM/MM-optimized geometry of the full system using the QM-VI region is shown in Figure S26a. Localized orbital analysis was next used to determine the d-electron count at Fe6 which gave the oxidation state as Fe^{3+} as 5 localized d-orbitals could be located (see Figure S26c). The appearance of approximate α and β versions of d-orbitals labeled as d_{xz} and d_{yz} (z-axis defined to be along the Fe-N bond) furthermore indicates spin-pairing and suggests a local low spin state of Fe^{3+}. This low spin-state assignment from localized orbitals is further supported by a low Hirshfeld spin population of -0.11 at Fe6.

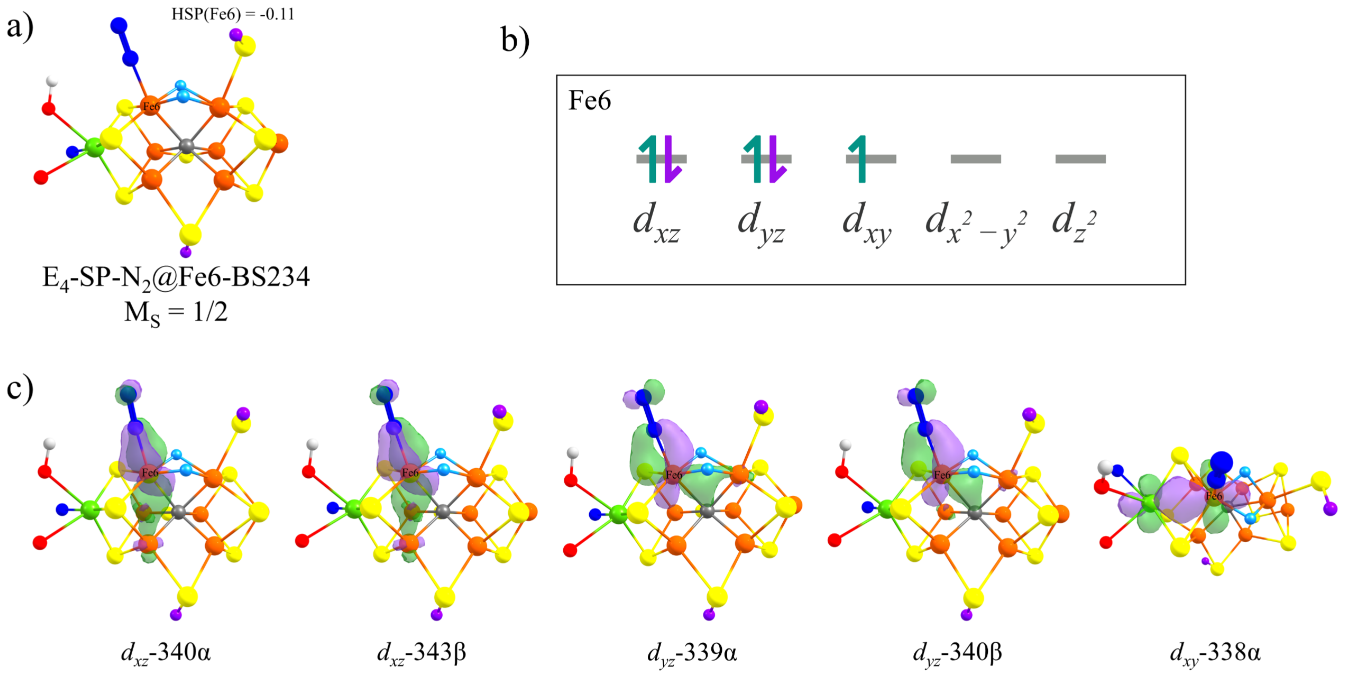

Figure S26. The structure (a), Hirshfeld spin population (HSP) and localized orbitals (c), and the approximate electronic configuration (based on localized orbitals) (b) of Fe6 for the full FeMoco of **E_4-SP-N_2@Fe6-BS234 (M_S = 1/2)** using the QM-VI region. According to localized orbitals, Fe6 is an Fe^{3+} (S = 1/2), which is supported by a low Hirshfeld spin population of Fe6 (-0.11).

Having assigned the local oxidation state and spin state of Fe6 to be an Fe^{3+} (S = 1/2), the full system was next substituted to a diamagnetic model (Figure S27b) with an Fe^{3+} (S = 1/2) in which Mo and Fe ions except Fe6 were replaced by In^{3+} and Ga^{3+}, respectively. With the system having only a single open-shell metal ion present, one can transform the canonical UKS orbitals (associated with a single open-shell metal ion) to useful quasi-restricted orbitals that have well-defined orbital energies. According to these quasi-restricted d-orbitals, as shown in Figure S27, an approximate ligand-field diagram (Figure S27b) was derived.

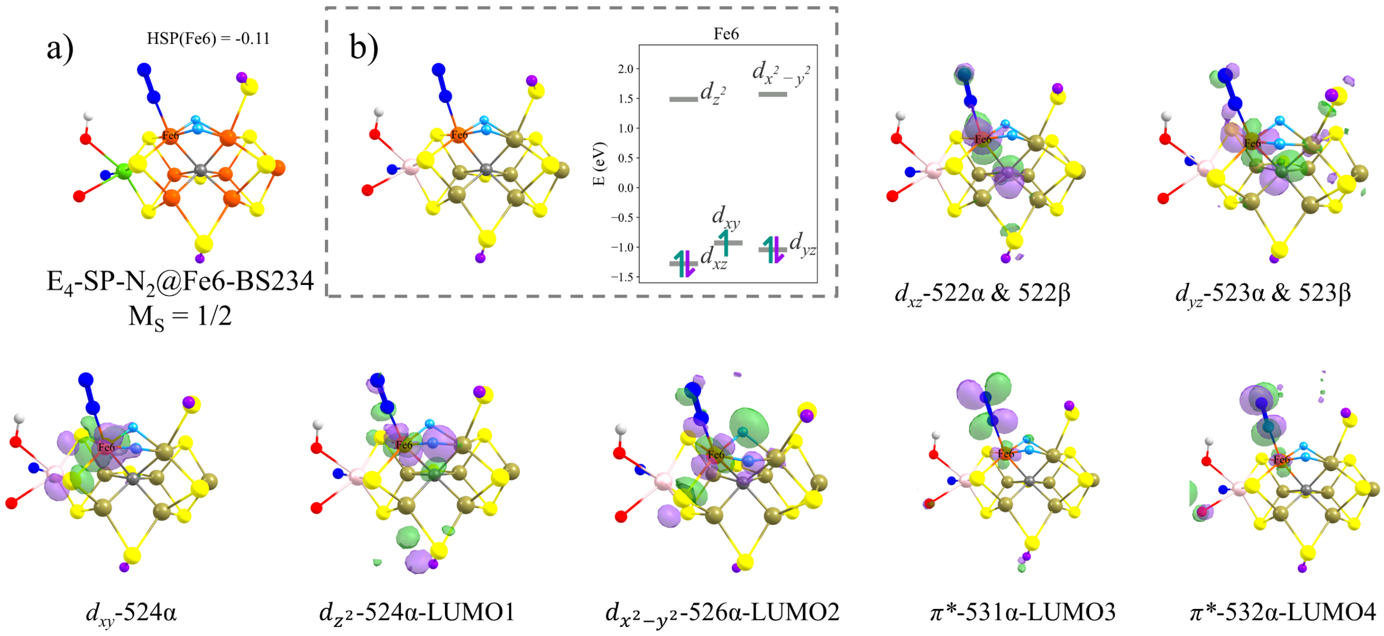

Figure S27. An example showing how QRO-based ligand-field diagrams can be derived based on the diamagnetically substituted approach. (a) The structure and Hirshfeld spin population (HSP) of the **E_4-SP** model with N_2 bound to Fe6 in the M_S = 1/2 and BS234 state. (b) The diamagnetically substituted model of Fe6 and associated QRO-based ligand-field diagram. Mo and Fe ions except Fe6 are diamagnetically replaced by In^{3+} and Ga^{3+}, respectively. Also shown are quasi-restricted d-orbitals of Fe6 and π^* orbitals of N_2. All results were obtained using the QM-VI region.

16. Spin-state energies of diamagnetically substituted models

To evaluate the reliability of QRO-based ligand-field diagrams of diamagnetically substituted models, **E_0-BS346 (M_S = 3/2), E_0-N_2@Fe6-BS147 (M_S = 1/2)**, and **E_4-SP-N_2@Fe6-BS234 (M_S = 1/2)** were studied using localized orbital analysis and spin-state energies of the diamagnetically substituted model.

Figure S28 shows the structure, localized orbitals, and relevant the approximate electronic configuration of Fe6 of the full FeMoco of **E_0-BS346 (M_S = 3/2)**. The localized orbital analysis indicates that Fe6 should be an Fe^{3+} (S = 5/2). The full FeMoco is next shown as diamagnetically substituted (Figure S28b).

Using the diamagnetically substituted model with a single Fe^{3+} ion present allows us next to calculate all 3 possible spin states for a single Fe^{3+} ion: S = 1/2, 3/2 and 5/2. The spin state energies (vertical) are shown in Table S7, and indicated (unsurprisingly) that the lowest energy spin-state is S = 5/2, consistent with the result of the localized orbital analysis and as expected for an approximately tetrahedral Fe ion in a weak-field sulfide (plus carbide) environment. Table S7 also shows a comparison of the Hirshfeld spin populations of Fe6 between the full FeMoco and diamagnetic models with three spin states (S = 1/2, 3/2, and 5/2). We note that while an Fe^{3+} with S = 3/2 (2.42) instead of S = 5/2 has a closer Hirshfeld spin population to the Hirshfeld spin population of Fe6 in the full FeMoco, the reason is that in the full system the Fe d-electrons are not completely localized (e.g. Mo and Fe6 partially share a d-electron) (as can be seen in Figure S28). This would make a Hirshfeld spin population of Fe6 of the full FeMoco lower than a localized Fe^{3+} (S = 5/2).

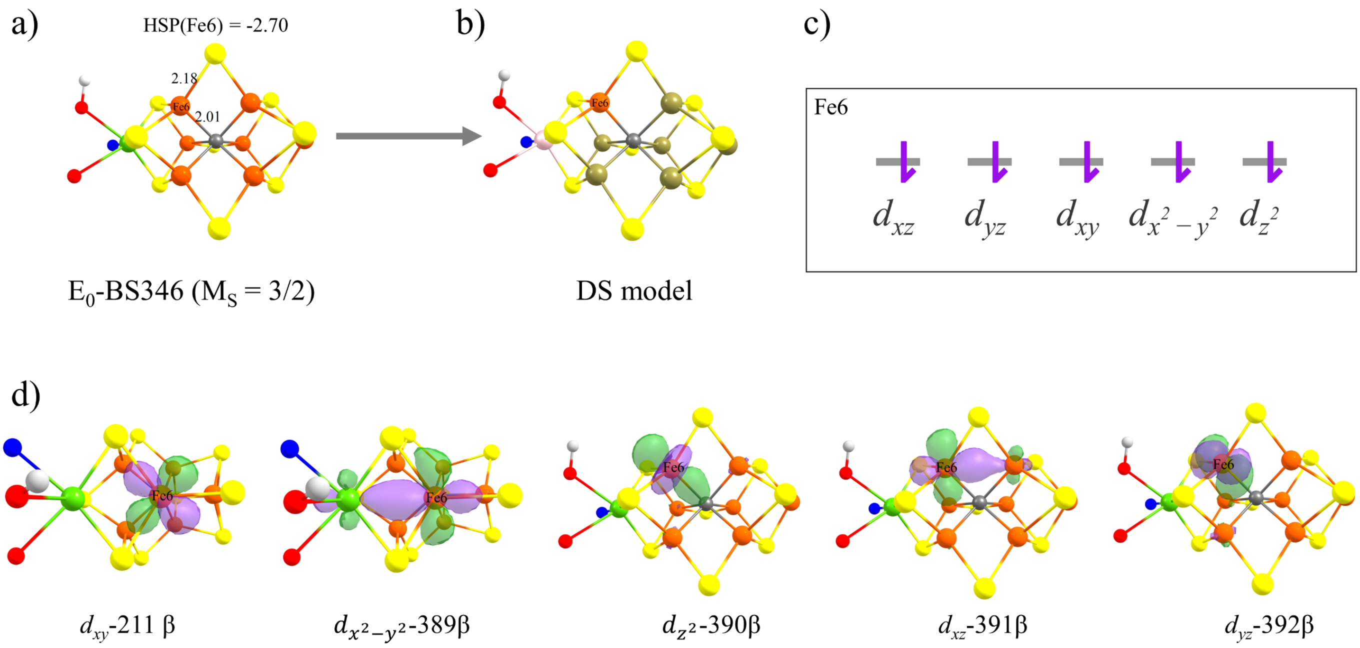

Figure S28. The structure (a), Hirshfeld spin population (HSP), and ligand-field diagram (c) of Fe6 derived from the localized orbital analysis (d) of the full FeMoco of **E_0-BS346 (M_S = 3/2)**. (b) The diamagnetically substituted model of **E_0-BS346 (M_S = 3/2)**. According to the localized orbital analysis, Fe6 is likely to be an Fe^{3+} (S = 5/2). All results were calculated using the QM-VI region.

Table S7. Relative energies for diamagnetically substituted (DS) models with an Fe^{3+} (S = 1/2, 3/2, or 5/2) built by using **E_0-BS346 (M_S = 3/2)** (Figure S28a).

**Full FeMoco**		**Spin pop.**
**Fe6**		-2.70
**DS FeMoco for Fe6**	**Relative polarized QM energies (kcal/mol)**	
**Fe6_III (S = 1/2)**	23.8	0.70
**Fe6_III (S = 3/2)**	17.1	2.42
**Fe6_III (S = 5/2)**	0.0	3.41

Note: The DS FeMoco model was calculated using the QM-VI region in the QM/MM level.

The same analysis was performed for **E_0-N_2@Fe6-BS147 (M_S = 1/2)**. Figure S29 shows the structure of the full FeMoco and diamagnetically substituted model of **E_0-N_2@Fe6-BS147 (M_S = 1/2)**. The localized orbitals of Fe6 of the full FeMoco (QM-VI region) and relevant electronic structure based on localized orbitals are also shown here. The localized orbital analysis suggests Fe6 is best described as an intermediate spin Fe^{2+} (S = 1). The spin-states of the diamagnetically substituted model with Fe^{2+} (S = 0, 1 or 2) were calculated. The lowest-energy model and the model with the closest Hirshfeld spin population to the full FeMoco were found to be the same one containing an Fe^{2+} (S = 1), again consistent with the localized orbital analysis of the full FeMoco (see Table S8). These results lend support to the diamagnetically substituted model as a reasonable way of getting insight into the ligand-field of localized Fe ions of FeMoco.

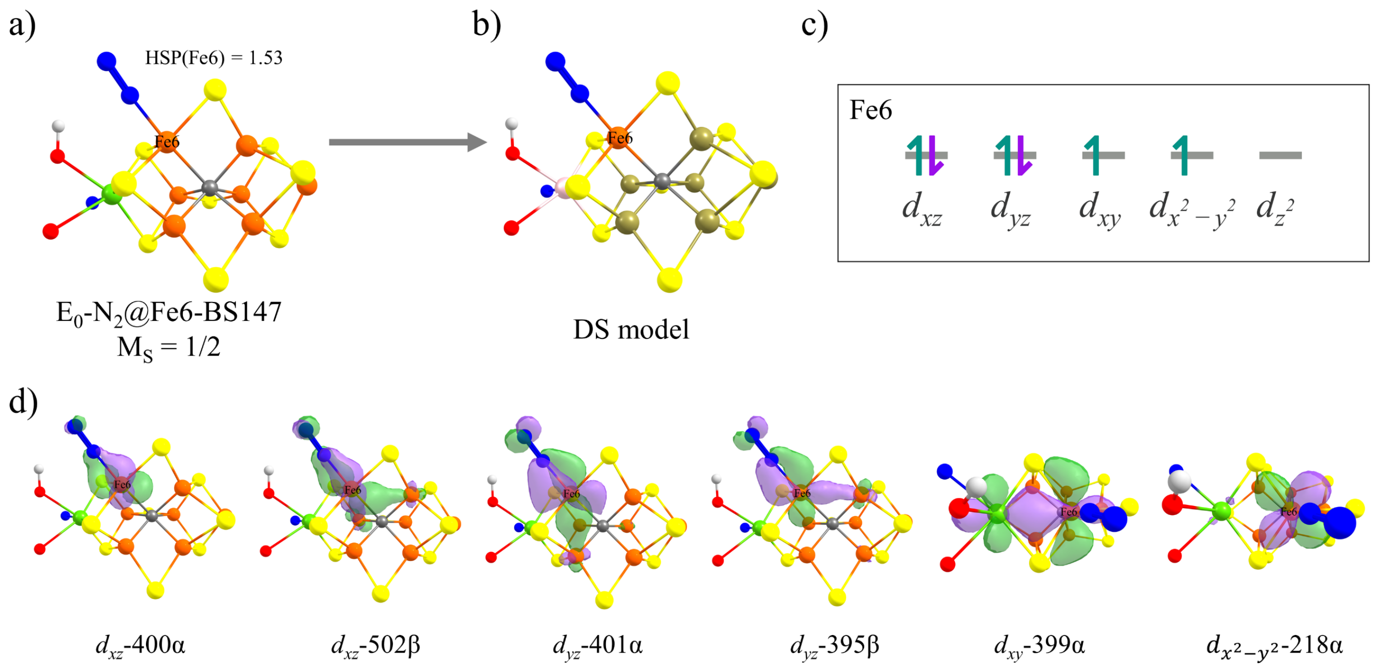

Figure S29. The structure (a), Hirshfeld spin population (HSP), electronic structures (c) based on localized orbitals (d) of Fe6 of the full FeMoco of **E_0-N_2@Fe6-BS147 (M_S = 1/2)** are shown here. According to the localized orbital analysis, Fe6 is an Fe^{2+} (S = 1). (b) The diamagnetically substituted model of **E_0-N_2@Fe6-BS147 (M_S = 1/2)**. All results were calculated using the QM-VI region.

Table S8. Relative energies between different spin states for the diamagnetically substituted (DS) model of Fe6 based on **E_0-N_2@Fe6-BS147** with M_S = 1/2 (Figure S29a).

**Full FeMoco**		**Spin pop.**
**Fe6**		1.53
**DS FeMoco for Fe6**	**Relative polarized QM energies (kcal/mol)**	
**Fe6_II (S = 0)**	15.4	0.00
**Fe6_II (S = 1)**	0.0	1.79
**Fe6_II (S = 2)**	12.3	3.13

Note: The DS FeMoco model was calculated using the QM-VI region in the QM/MM level.

E_4-SP-N_2@Fe6-BS234 with M_S = 1/2 was also investigated to evaluate the reasonableness of our diamagnetically substituted approach. The localized orbital analysis of Fe6 of **E_4-SP-N_2@Fe6-BS234** with M_S = 1/2 (Figure S26) indicates that Fe6 should be a low-spin Fe^{3+}. The full FeMoco was utilized to build diamagnetically substituted models with an Fe^{3+} (S = 1/2, 3/2, or 5/2), as shown in Table S9, showing that the model with a low-spin Fe^{3+} is the lowest-energy model and has the closest Hirshfeld spin population to the full FeMoco.

All in all, the analysis of relative energies and Hirshfeld spin populations among diamagnetically substituted models with possible spin states for **E_0-BS346 (M_S = 3/2), E_0-N_2@Fe6-BS147 (M_S = 1/2)**, and **E_4-SP-N_2@Fe6-BS234 (M_S = 1/2)** supports that our diamagnetically substituted approach appears to be a reasonable one for deriving QRO-based

ligand-field diagrams.

Table S9. Relative energies between different spin states for diamagnetically substituted models of **E_4-SP-N_2@Fe6-BS234** with M_S = 1/2 (Figure S26a).

**Full FeMoco**		**Spin pop.**
**Fe6**		-0.11
**DS FeMoco for Fe6**	**Relative polarized QM energies (kcal/mol)**	
**Fe6_III (S = 1/2)**	0.0	0.30
**Fe6_III (S = 3/2)**	45.1	2.48
**Fe6_III (S = 5/2)**	86.1	3.47

Note: The DS FeMoco model was calculated using the QM-VI region in the QM/MM level.

17. The energy difference between E_2-hyd and E_2-nonhyd models

In our previous QM/MM study by Thorhallsson and Bjornsson, the energy difference between **E_2-hyd** and **E_2-nonhyd** models was reported to be 0.1 kcal/mol at the TPSSh-QM/MM level.[7] However, in this work using a new QM/MM model and another functional (r^2SCAN) the energy difference was instead found to be 13.6 kcal/mol (QM-I region) and 12.1 kcal/mol (QM-VI region).

We carried out a systematic investigation into this reproducibility issue, comparing QM region size, different programs used (ASH vs. Chemshell), different functionals (TPSSh vs. r^2SCAN), as well as the size of the whole QM/MM model (the whole 320814 atom solvated box vs. the 36988 atom spherical truncated system). The lowest-energy isomer of **E_2-nonhyd** models is calculated to be **E_2-nonhyd-BS346** with M_S = 3/2 in our study, which is the same as in the previous study. For **E_2-hyd** models, **E_2-hyd-BS235** with M_S = 3/2 and **E_2-hyd-BS247** with M_S = 3/2 are the lowest-energy isomers in both studies. **E_2-hyd-BS235** in the M_S = 3/2 spin state is slightly lower than **E_2-hyd-BS247** with M_S = 3/2 by 0.7 kcal/mol when using the **QM-I** region in our study. However, **E_2-hyd-BS235 (M_S = 3/2)** is a bit higher than **E_2-hyd-BS247 (M_S = 3/2)** by 0.3 kcal/mol when they used the QM-V region. Therefore, comparing **E_2-hyd-BS247 (M_S = 3/2)** of Thorhallsson's result with **E_2-hyd-BS235** with M_S = 3/2 of our present study is reasonable. The QM-V region is defined the same in both studies.

Calculations on the same truncated 36988 atom spherical model was compared using both Chemshell and ASH as QM/MM programs using the TPSSh functional. The small energy difference could be reproduced reasonably well (0.1 kcal/mol using Chemshell vs. 0.5 kcal/mol using ASH) suggesting details differing between two programs to not be the reason for the different results.

The functional, however, turned out to be the main reason: r^2SCAN being used in this work while TPSSh was used by Thorhallsson et al. These two functionals were previously reported to reproduce the structure of FeMoco about equally well when compared against the high-resolution X-ray structure.[3] However, the **E_2** is a more complicated state than the **E_0** and the electronic distributions of **E_2-hyd** and **E_2-nonhyd** are very different. The two additional electrons in the **E_2** state are located on the hydride between Fe2 and Fe6 for the **E_2-hyd** models (Figure S12). In contrast, these two electrons are assigned among irons in the FeMoco for **E_2-nonhyd** models (Figure S14). The energy difference between **E_2-hyd** and **E_2-nonhyd** models increased to 8.7 kcal/mol when using ASH, r^2SCAN, and the same QM/MM system as their study, as shown in the 9th and 10th rows of Table S10. The 3rd and 4th only have one difference from the 9th and 10th: the functional. Thus, the functional appears to be the primary reason for the energy difference.

Finally, the size of the QM/MM system appears to be another factor. Both Thorhallsson's and our QM/MM system came from the same molecular dynamics (MD) simulation. However, in this work the whole solvated protein box was used which should be a more realistic model than the truncated model used in ref. 7. The active region was almost identical in both setups with the model size difference affecting primarily the the polarized QM energy via a larger MM pointcharge environment in our work compared to ref. 7. When ASH calculations using the r^2SCAN functional and the QM-V region are carried out on the sphere model the energy difference is 8.7 kcal/mol (**E_2-hyd** more stable) but this increased to 11.5 kcal/mol for the larger cube model. The size of the QM/MM model is hence a factor to pay attention to when comparing results of different QM/MM studies of nitrogenase.

These results appear also to be related to the relatively large electronic structure difference between **E_2-hyd** and **E_2-nonhyd**. The **E_2-nonhyd** state is in fact a problematic state as well as detailed in the next section.

Table S10. Energy differences of **E_2-hyd** and **E_2-nonhyd** models without N_2.

	Mulliken spin population	sizes of the whole QM/MM system	programs	functionals	sizes of QM regions	ΔE (polarized QM)	ΔE (QM/MM)	Mo[a]	Fe1	Fe2	Fe3	Fe4	Fe5	Fe6	Fe7
1	nonhydride_mult4_BS346	sphere[c]	Chemshell	TPSSh	QM-V	1.1	0.0	-0.24	3.36	2.98	-3.21	-3.29	3.07	-2.55	2.86
2	hydride_mult4_BS247[b]	sphere	Chemshell	TPSSh	QM-V	0.0	0.1	-0.48	3.42	-3.15	3.31	-3.16	2.98	2.90	-3.02
3	nonhydride_mult4_BS346	sphere	ASH	TPSSh	QM-V	0.3	0.0	-0.25	3.35	2.96	-3.20	-3.29	3.07	-2.55	2.84
4	hydride_mult4_BS247	sphere	ASH	TPSSh	QM-V	0.0	0.5	-0.50	3.42	-3.15	3.30	-3.17	3.00	2.88	-3.02
5	nonhydride_mult4_BS346	sphere	ASH	TPSSh	QM-III	0.0	0.0	-0.25	3.36	2.95	-3.20	-3.29	3.07	-2.54	2.84
6	hydride_mult4_BS247	sphere	ASH	TPSSh	QM-III	0.5	0.4	-0.51	3.42	-3.14	3.30	-3.17	3.00	2.89	-3.02
7	nonhydride_mult4_BS346	sphere	ASH	TPSSh	QM-I	4.4	3.0	-0.32	3.36	2.96	-3.2	-3.3	3.07	-2.51	2.86

**8**	**hydride_mult4_BS247**	sphere	ASH	TPSSh	QM-I	0.0	0.0	-0.49	3.43	-3.15	3.32	-3.19	2.97	2.89	-3.02
**9**	**nonhydride_mult4_BS346**	sphere	ASH	r^2SCAN	QM-V	9.6	8.7	-0.34	3.23	2.59	-2.99	-3.12	2.92	-2.09	2.63
**10**	**hydride_mult4_BS247**	sphere	ASH	r^2SCAN	QM-V	0.0	0.0	-0.41	3.28	-2.93	3.13	-3.03	2.81	2.78	-2.87
**11**	**nonhydride_mult4_BS346**	cube[d]	ASH	TPSSh	QM-III	6.7	5.2	-0.39	3.43	3.28	-3.15	-3.26	3.00	-2.61	2.39
**12**	**hydride_mult4_BS247**	cube	ASH	TPSSh	QM-III	0.0	0.0	-0.47	3.44	-3.15	3.30	-3.17	2.99	2.89	-3.03
**13**	**nonhydride_mult4_BS346**	cube	ASH	TPSSh	QM-I	9.1	5.4	-0.43	3.43	3.27	-3.16	-3.26	3.01	-2.61	2.43
**14**	**hydride_mult4_BS247**	cube	ASH	TPSSh	QM-I	0.0	0.0	-0.46	3.43	-3.16	3.31	-3.18	2.98	2.92	-3.05
**15**	**nonhydride_mult4_BS346**	cube	ASH	r^2SCAN	QM-I	2.2	12.0	-0.43	3.30	3.10	-2.94	-3.08	2.85	-2.16	1.97
**16**	**hydride_mult4_BS235[e]**	cube	ASH	r^2SCAN	QM-I	0.0	0.0	-0.47	3.32	-2.96	-3.09	3.14	-2.97	2.83	2.69
**17**	**nonhydride_mult4_BS346**	cube	ASH	r^2SCAN	QM-III	14.2	12.9	-0.40	3.32	3.10	-2.91	-3.06	2.85	-2.19	1.89
**18**	**hydride_mult4_BS235**	cube	ASH	r^2SCAN	QM-III	0.0	0.0	-0.46	3.32	-2.96	-3.06	3.13	-2.97	2.85	2.68
**19**	**nonhydride_mult4_BS346**	cube	ASH	r^2SCAN	QM-V	14.6	11.5	-0.37	3.30	3.12	-2.89	-3.07	2.85	-2.15	1.85
**20**	**hydride_mult4_BS235**	cube	ASH	r^2SCAN	QM-V	0.0	0.0	-0.45	3.31	-2.95	-3.06	3.13	-2.97	2.84	2.68

[a] The last eight columns show Mulliken spin multiplicities of metal centers in the FeMoco.

[b] The lowest isomer of **E_2-hyd** in energy is **E_2-hyd-BS247** with $M_S = 3/2$ in the previous study. The data of the first two rows were copied from the previous paper[7].

[c] The cutting spherical model was used in the previous paper by Thorhallsson et al.

[d] The whole cubic system without any cutting is the system we used in the present study.

[e] The lowest-energy isomer of **E_2-hyd** models is **E_2-hyd-BS235** with $M_S = 3/2$ when using the QM-I region in the present study.

18. Electronic structure sensitivity of the E_2-nonhyd isomer

This section discusses two distinct electronic configurations of the **E_2-nonhyd-BS346** isomer (without N_2) that are encountered in calculations. We find that BS-DFT calculations of this isomer are extremely sensitive to the details of the calculation setup and document it here for future reference.

There are two sub-cubanes in FeMoco: $MoFe_3$ sub-cubane and Fe_4 sub-cubane. The former contains Mo and Fe5, Fe6, and Fe7, and the latter includes Fe1, Fe2, Fe3, and Fe4. In the **E_0** state, there are 19 and 22 d-electrons distributed among of metal atoms of $MoFe_3$ sub-cubane and Fe_4 sub-cubane, respectively, according to the localized analysis (Figure S5). In our previous study[7] on the **E_2** state, 21 d-electrons are among metal atoms of $MoFe_3$ sub-cubane. In other words, two additional electrons locate on metal atoms of $MoFe_3$ sub-cubane. In this study, we found that two additional electrons in the $MoFe_3$ sub-cubane or one additional electron in each $MoFe_3$ sub-cubane and Fe_4 sub-cubane are possible.

Two isomers for **E_2-nonhyd-BS346** with M_S = 3/2 could be found, with the result depending on both the size of the QM-region, what initial structure was used as well as how the environment was treated. The QM/MM energy difference between these two isomers is 0 ~ 2 kcal/mol, and these two isomers actually have different electronic configurations according to the localized orbital analysis. When the QM-I region is used, *isomer1* has one additional electron in each $MoFe_3$ sub-cubane and Fe_4 sub-cubane (the first two rows in Table S11). In contrast, the $MoFe_3$ sub-cubane of *isomer2* contains two additional electrons. When r^{2}SCAN is replaced with TPSSh, the localized orbital analysis is similar (the 3rd and 4th rows of Table S11).

As the QM region was expanded step by step, the localized orbital analysis reveals that the results were reproducible for some QM-regions but not all (e.g. QM-IV). Single-point cluster-continuum calculations on the QM/MM geometries using different values of the dielectric constant, however, resulted in an electronic distribution over both sub-cubanes, according to the localized orbital analysis, suggesting the 2 difference electronic configurations is sensitive to the electrostatic environment around the cofactor.

Overall, the results reveal that the **E_2-nonhyd** isomer has considerable electronic structure complexity with multiple SCF solutions available to this redox state (even when flipping the same Fe ions) that may lead to different electronic configurations and different energies resulting from each geometry optimization. Calculations and interpretations of the **E_2-nonhyd** isomer thus need to be taken with a grain of salt and this redox state requires further analysis.

Table S11. Energies and electron distribution counts between sub-cubanes based on localized orbital analysis for two isomers of **E_2-nonhyd-BS346** in M_S = 3/2.

		**ΔE(polarized QM)**	**ΔE(MM)**	**ΔE(QM/MM)**	**$MoFe_3$ sub-cubane | Fe_4 sub-cubane**
**1**	**E_2-nonhyd-BS346-QM-I-r^{2}SCAN-isomer1 (M_S = 3/2)**	1.2	0.6	1.8	1 | 1
**2**	**E_2-nonhyd-BS346-QM-I-r^{2}SCAN-isomer2 (M_S = 3/2)**	0.0	0.0	0.0	2 | 0
**3**	**E_2-nonhyd-BS346-QM-I-TPSSh-isomer1 (M_S = 3/2)**	0.9	0.0	0.2	1 | 1
**4**	**E_2-nonhyd-BS346-QM-I-TPSSh-isomer2 (M_S = 3/2)**	0.0	0.7	0.0	2 | 0
**5**	**E_2-nonhyd-BS346-QM-II-r^{2}SCAN-isomer1 (M_S = 3/2)**	0.0	5.7	0.4	1 | 1
**6**	**E_2-nonhyd-BS346-QM-II-r^{2}SCAN-isomer2 (M_S = 3/2)**	5.3	0.0	0.0	2 | 0
**7**	**E_2-nonhyd-BS346-QM-III-r^{2}SCAN-isomer1 (M_S = 3/2)**	0.0	1.7	1.0	1 | 1
**8**	**E_2-nonhyd-BS346-QM-III-r^{2}SCAN-isomer2 (M_S = 3/2)**	0.8	0.0	0.0	2 | 0
**9**	**E_2-nonhyd-BS346-QM-III-TPSSh-isomer2 (M_S = 3/2)**	-	-	-	2 | 0
**10**	**E_2-nonhyd-BS346-QM-IV-r^{2}SCAN-isomer1 (M_S = 3/2)**	0.0	5.2	1.4	1 | 1
**11**	**E_2-nonhyd-BS346-QM-IV-r^{2}SCAN-isomer2 (M_S = 3/2)**	3.8	0.0	0.0	1 | 1
**12**	**E_2-nonhyd-BS346-QM-V-r^{2}SCAN-isomer1 (M_S = 3/2)**	0.0	6.1	1.2	1 | 1
**13**	**E_2-nonhyd-BS346-QM-V-r^{2}SCAN-isomer2 (M_S = 3/2)**	4.9	0.0	0.0	2 | 0
**14**	**E_2-nonhyd-BS346-cluster-QM-I-r^{2}SCAN-isomer1 (M_S = 3/2, ε = 4)**	0.0	0.0	0.0	1 | 1
**15**	**E_2-nonhyd-BS346-cluster-QM-I-r^{2}SCAN-isomer2 (M_S = 3/2, ε = 4)**	2.6	0.0	2.6	1 | 1
**16**	**E_2-nonhyd-BS346-cluster-QM-I-r^{2}SCAN-isomer1 (M_S = 3/2, ε = 100)**	0.0	0.0	0.0	1 | 1
**17**	**E_2-nonhyd-BS346-cluster-QM-I-r^{2}SCAN-isomer2 (M_S = 3/2, ε = 100)**	2.7	0.0	2.7	1 | 1

19. Additional N_2 binding modes

Besides the end-on (*trans* to Fe-C) N_2 binding primarily discussed in this work, there are additional N_2 binding modes to FeMoco that have been suggested in previous papers[8-11], some of which are shown in the Figure S30.

Apart from the *trans*-end-on mode primarily explored in the manuscript, we also tested other possibilities shown in Figure S30. We used **E_2-hyd** as a test case for these explorations, comparing isomers with the SH-group on either Fe2 or Fe6. When the SH-group is coordinated to Fe2, three types of binding modes (side-on, end-on, and end-on side-on) in a *cis* position (to Fe-C) were tested, however, all of these QM/MM geometry optimizations (case of b, c, and d) either converged to the *trans*-end-on mode previously calculated or N_2 dissociated from the cofactor (case of f). When the SH-group is located on Fe6, N_2 in the *cis*-side-on mode dissociated. For the case of N_2 bound *cis*-end-on (case e), a minima was found but the binding energy is much higher than the regular *trans*-end-on mode (a); this energy penalty is due to steric clashing with residues Arg96 and Val70. When calculating N_2 as partially bridging, *cis*-end-on/side-on (case h) the optimization converged to a *trans*-side-on structure on Fe2; this structure is overall very unfavourable compared to the simple *trans*-end-on geometry seen in a.

Generally, N_2 bound in a *cis* position (of the Fe-C bond) seems unlikely due to the protein environment restricting such geometries. Furthermore, side-on structures are generally unfavourable and overall end-on N_2-binding geometries *trans* to the Fe-C bond appears to be the most relevant N_2-bound geometry at FeMoco.

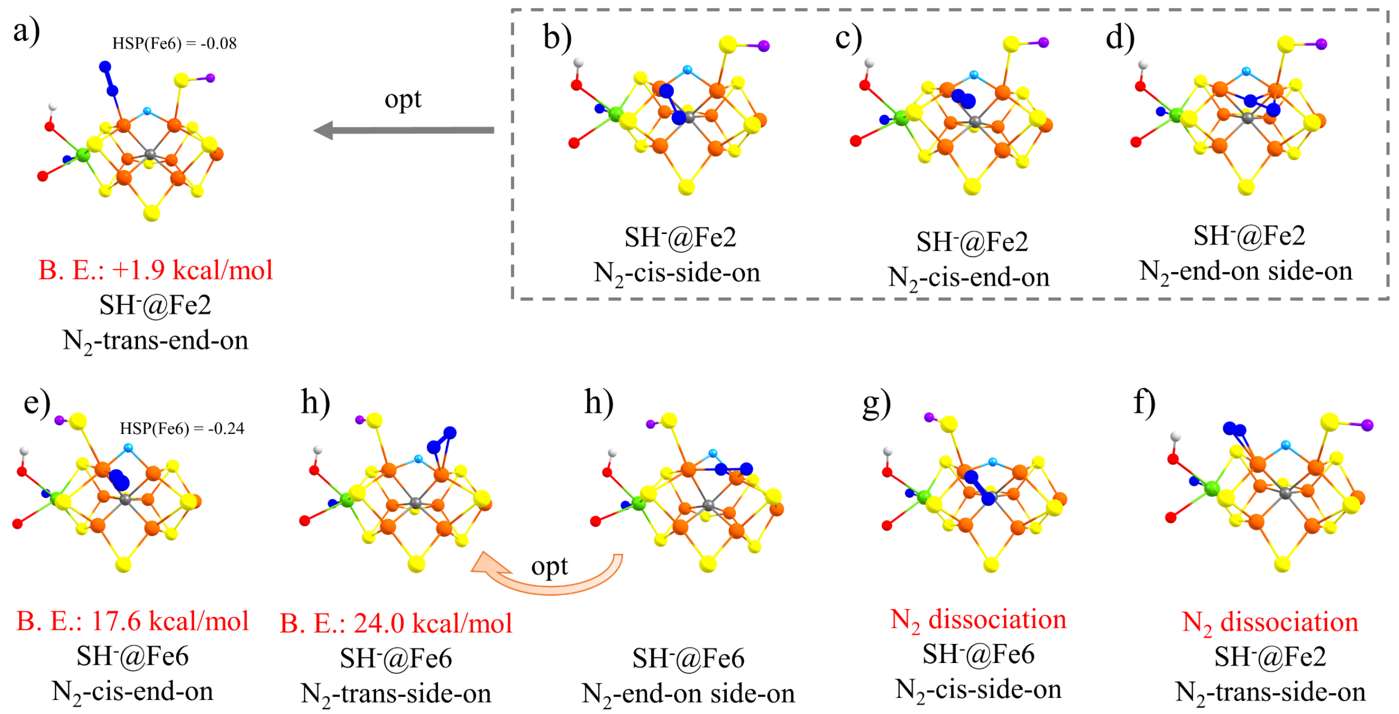

Figure S30. Additional N_2 binding modes tested using the QM-VI region. All structures were calculated using the lowest-energy BS solution and spin state (M_S = 1/2, BS235) in the **E_2-hyd-N_2** state when N_2 binding in the *trans*-end-on mode. All binding energies of these modes have the same reference **(E_2-hyd-SH^-@Fe6 (M_S = 3/2)**, shown in the left structure of Figure 7a).

20. Investigation of the integration grid dependence in r^{2}SCAN calculations

We evaluated the grid noise in our r^{2}SCAN/MM calculations using the **E_0-BS235 (M_S = 3/2)** state with a QM-I region. All calculations were performed using ORCA version 5 that comes with a new recommended set of default grids (defgridX). ORCA's molecular grids are constructed from atomic grids using Becke's approach, using Lebedev angular grids and radial grids using a modified Gauss-Chebyschev quadrature with M3 mapping by Treutler and Ahlrichs. The number of radial points is determined by a formula by Krack and Köster. The angular grids are also pruned according to a 5-region pruning scheme. Machine learning techniques were used to find the best values for the final parameters defining the final grids: including the atomic M3 mapping parameters, the b parameter in the Krack-Köster formula and the cutoffs for the region-based pruning scheme. See ORCA manual for details.

The "refgrid" setting in ORCA consists of a highly dense grid that is assumed to be close to the grid-infinite limit; it consists of a Lebedev770 angular grid and an IntAcc of 10.0 (ε in the Krack-Köster formula). The "defgrid2" setting is the new default grid-setting in ORCA version 5 and was primarily used in this work; it consists of a Lebedev302 angular grid and an IntAcc parameter of 4.388. The "defgrid1" is a smaller grid (Lebedev194, IntAcc=4.159) while "defgrid3" is larger (Lebedev590, IntAcc=4.959).

Table S12 shows the results of r^{2}SCAN/MM and TPSSh/MM calculations with different grids. Absolute QM-energies and integrated number of electrons for the **E_0-BS235** state are shown relative to the "refgrid" result. The QM-energy comparison suggests that grid-noise is slightly larger for r^{2}SCAN compared to TPSSh. The "defgrid1" calculation gives a 7.1 kcal/mol absolute energy error (compared to "refgrid"). This is less for "defgrid2" (-2.8 kcal/mol) and very small for "defgrid3" (-0.2 kcal/mol). However, some of this absolute numerical integration error is expected to be present in the core-region where lower-grids and grid pruning is deliberately applied; for energy differences involving changes in valence electron structure, this error would cancel out to some extent. This in fact happens when a relative energy such as the N_2 binding energy is calculated (**E_0-BS235** + N_2 → **E_0-BS235-N_2**). Grid errors for the binding energy are: 0.5 kcal/mol ("defgrid1"), 0.02 kcal/mol ("defgrid2") and 0.2 kcal/mol ("defgrid3"). These results suggest that the grid errors for relative energies are mostly acceptable, perhaps being slightly too large for "defgrid1". Slightly better error cancellation is seen for "defgrid2" than "defgrid3" but overall we estimate a grid uncertainty of ~0.2 kcal/mol in our "defgrid2" calculations.

Table S12. The energy difference and binding energies between refgrid, defgrid1, defgrid2, and defgrid3 using r^{2}SCAN and TPSSh (kcal/mol).

		Num. grid points	Δ(QM energy[a])	Δ(integrated electrons)	Δ(B. E.[b])-QM
r^{2}SCAN	defgrid1	179518	7.1	0.0007	0.5
	defgrid2	348076	-2.8	-0.0004	0.0
	defgrid3	840700	-0.2	0.0000	0.2
	refgrid	3877088	0.0	0.0000	0.0
TPSSh	defgrid1	179458	-1.6	0.0007	
	defgrid2	347987	-0.9	-0.0004	
	defgrid3	840465	0.1	0.0000	
	refgrid	3876531	0.0	0.0000	

[a] The QM energy is a polarized QM energy in this table.
[b] B. E. refers to the N_2 binding energy, and the N_2 bound state is the **E_0-BS235-N_2@Fe6-BS147 (M_S = 1/2)**.
Note: All results were obtained using the QM-I region.

21. Relative energies, vibrational frequencies and Hirshfeld spin populations of all calculated models

Tables S13 to S17 show relative energies of different electronic states (ΔE), N_2 binding energies (B. E.), as well as single-step binding energies (S. B. E.) in each redox state. Shown are polarized QM energy differences and QM/MM energy differences, absolute and relative vibrational frequencies (relative to free N_2, $\nu_{NN,\, free} = 2432\ cm^{-1}$) and Hirshfeld spin populations for the metal ions.

Table S13. Results of $\mathbf{E_0}$ models calculated using both QM-I and QM-VI regions. N_2 binding energies are relative to **$\mathbf{E_0}$-BS235 ($\mathbf{M_S = 3/2}$)**.

QM-I region	ΔE(QM)	ΔE(QM/MM)	B. E. (QM)	B. E. (QMMM)	υ(N-N)	Rel. υ(N-N)	Mo	Fe1	Fe2	Fe3	Fe4	Fe5	Fe6	Fe7
**E_0-N_2@Fe6-BS147 (M_S = 1/2)**	0.0	0.0	17.4	16.3	2205	-227	-0.18	-3.12	2.82	2.82	-2.98	2.80	1.49	-2.59
**E_0-N_2@Fe2-BS147 (M_S = 1/2)**	6.6	6.9	24.0	23.2	2215	-217	-0.28	-3.12	2.01	2.96	-3.04	2.61	2.59	-2.56
**E_0-N_2@Fe2-BS346 (M_S = 1/2)**	7.0	8.6	24.4	24.9	2201	-231	-0.13	3.16	1.86	-2.94	-2.90	2.53	-2.73	2.46
**E_0-BS147 (M_S = 3/2)**	8.2	7.2					-0.34	-3.16	3.12	2.95	-3.00	2.79	2.89	-2.51
**E_0-BS235 (M_S = 3/2)**	0.8	0.0					-0.33	3.17	-2.79	-2.85	3.01	-2.78	2.57	2.58
**E_0-BS247 (M_S = 3/2)**	2.7	2.0					-0.27	3.14	-2.79	3.00	-2.84	2.66	2.54	-2.72
**E_0-BS346 (M_S = 3/2)**	0.0	0.0					-0.16	3.15	2.97	-2.86	-2.86	2.67	-2.72	2.55
**E_0-BS147 (M_S = 1/2)**	12.4	10.5					-0.23	-3.14	2.62	2.76	-3.09	2.63	2.10	-2.55
**E_0-BS235 (M_S = 1/2)**	18.6	16.7					-0.33	1.77	-2.84	-2.87	2.80	-2.77	2.57	2.60
**E_0-BS247 (M_S = 1/2)**	16.2	15.2					-0.20	3.09	-3.04	2.53	-3.08	2.44	2.25	-2.77
**E_0-BS346 (M_S = 1/2)**	18.1	16.7					-0.17	1.77	2.70	-2.86	-2.92	2.68	-2.70	2.56
**QM-VI region**														
**E_0-N_2@Fe6-BS147 (M_S = 1/2)**	0.0	0.0	15.0	16.6	2200	-232	-0.18	-3.11	2.79	2.78	-2.96	2.78	1.53	-2.57
**E_0-N_2@Fe2-BS147 (M_S = 1/2)**	7.9	4.8	22.9	21.4	2212	-220	-0.27	-3.11	2.01	2.93	-3.02	2.61	2.58	-2.56
**E_0-BS235 (M_S = 3/2)**							-0.32	3.16	-2.78	-2.83	2.99	-2.77	2.56	2.56

Table S14. Results of $\mathbf{E_0H^+}$ models calculated using both QM-I and QM-VI regions. N_2 binding energies are relative to **$\mathbf{E_0H^+}$-BS235 ($\mathbf{M_S = 3/2}$)**.

QM-I region	ΔE(QM)	ΔE(QM/MM)	B. E. (QM)	B. E. (QMMM)	υ(N-N)	Rel. υ(N-N)	Mo	Fe1	Fe2	Fe3	Fe4	Fe5	Fe6	Fe7
**E_0H^+-N_2@Fe6-BS147 (M_S = 1/2)**	0.0	-4.4	17.9	18.6	2220	-212	-0.19	-3.08	2.78	2.80	-2.94	2.80	1.61	-2.64
**E_0H^+-N_2@Fe6-BS235 (M_S = 1/2)**	4.1	-4.6	21.9	18.4	2188	-244	-0.13	3.10	-2.82	-2.86	2.94	-2.81	0.85	2.62
**E_0H^+-N_2@Fe6-BS247 (M_S = 1/2)**	3.0	-1.1	20.9	21.9	2220	-212	-0.02	3.06	-2.74	2.94	-2.85	2.62	0.79	-2.78
**E_0H^+-N_2@Fe6-BS346 (M_S = 1/2)**	11.4	4.3	29.3	27.3	2253	-179	-0.14	3.05	0.66	-2.93	-2.88	2.68	-2.35	2.62
**E_0H^+-N_2@Fe2-BS147 (M_S = 3/2)**	17.1	4.4	35.0	27.4	2261	-171	-0.29	-3.13	2.57	3.05	-2.84	2.82	3.00	-2.38
**E_0H^+-N_2@Fe2-BS235 (M_S = 3/2)**	12.0	0.0	29.9	23.0	2262	-170	-0.15	3.12	-2.49	-2.94	2.89	-2.79	2.43	2.58
**E_0H^+-N_2@Fe2-BS147 (M_S = 1/2)**	15.2	0.1	33.1	23.1	2246	-186	-0.20	-3.12	2.09	2.83	-2.92	2.60	2.67	-2.73
**E_0H^+-N_2@Fe2-BS346 (M_S = 1/2)**	18.3	4.8	36.2	27.8	2232	-200	-0.35	3.11	0.63	-2.77	-2.70	2.80	-2.75	2.73
**E_0H^+-BS147 (M_S = 3/2)**	6.5	6.6					-0.31	-3.12	3.10	2.99	-2.95	2.82	2.87	-2.58
**E_0H^+-BS235 (M_S = 3/2)**	0.0	0.0					-0.28	3.13	-2.75	-2.87	2.99	-2.76	2.51	2.65
**E_0H^+-BS247 (M_S = 3/2)**	0.9	1.3					-0.18	3.09	-2.71	2.99	-2.84	2.70	2.45	-2.74
**E_0H^+-BS346 (M_S = 3/2)**	2.5	3.4					-0.26	3.17	2.91	-2.85	-2.83	2.64	-2.69	2.56
**E_0H^+-BS147 (M_S = 1/2)**	10.0	10.2					-0.21	-3.10	2.81	2.73	-3.03	2.60	2.02	-2.63
**E_0H^+-BS235 (M_S = 1/2)**	16.8	16.1					-0.28	1.72	-2.77	-2.87	2.76	-2.76	2.52	2.67
**E_0H^+-BS247 (M_S = 1/2)**	20.1	18.8					-0.20	1.84	-2.83	2.66	-2.83	2.70	2.47	-2.71
**E_0H^+-BS346 (M_S = 1/2)**	15.0	14.6					-0.25	3.07	0.68	-2.76	-2.73	2.70	-2.60	2.65
**QM-VI region**														
**E_0H^+-N_2@Fe2-BS235 (M_S = 3/2)**	3.1	0.0	16.4	16.8	2256	-177	-0.18	3.12	-2.61	-2.92	2.89	-2.76	2.54	2.53
**E_0H^+-N_2@Fe6-BS235 (M_S =1/2)**	0.0	1.0	13.3	17.8	2193	-239	-0.13	3.08	-2.80	-2.84	2.94	-2.80	0.83	2.59
**E_0H^+-BS235 (M_S =3/2)**							-0.27	3.12	-2.75	-2.84	2.99	-2.75	2.50	2.63

Table S15. Results of $\mathbf{E_1}$ models calculated using both QM-I and QM-VI regions. N_2 binding energies are relative to **$\mathbf{E_1}$-BS346 ($\mathbf{M_S = 2}$)**.

QM-I region	ΔE(QM)	ΔE(QM/MM)	B. E. (QM)	B. E. (QMMM)	υ(N-N)	Rel. υ(N-N)	Mo	Fe1	Fe2	Fe3	Fe4	Fe5	Fe6	Fe7
**E_1-N_2@Fe6-BS346 (M_S = 2)**	3.0	0.0	15.7	12.0	2176	-256	-0.36	3.19	2.92	-2.84	-2.86	2.69	-1.96	2.60
**E_1-N_2@Fe6-BS235 (M_S = 1)**	0.0	1.1	12.7	13.1	2172	-260	-0.36	3.15	-2.79	-2.88	2.96	-2.80	2.13	2.52
**E_1-N_2@Fe6-BS247 (M_S = 1)**	1.0	2.3	13.7	14.3	2172	-260	-0.25	3.12	-2.78	2.96	-2.85	2.61	1.99	-2.74
**E_1-N_2@Fe6-BS346 (M_S = 1)**	7.3	6.5	20.0	18.5	2171	-261	-0.14	3.15	1.94	-3.02	-3.02	2.61	-2.19	2.48
**E_1-N_2@Fe2-BS147 (M_S = 2)**	24.8	15.1	37.5	27.1	2202	-230	-0.53	-3.15	2.47	3.10	-2.80	2.85	3.17	-1.40
**E_1-N_2@Fe2-BS235 (M_S = 2)**	14.7	4.7	27.4	16.7	2193	-239	-0.24	3.17	-2.08	-2.99	2.97	-2.69	2.72	2.57
**E_1-N_2@Fe2-BS247 (M_S = 2)**	16.2	7.2	28.9	19.2	2192	-240	-0.17	3.17	-2.06	2.94	-2.96	2.67	2.65	-2.65
**E_1-N_2@Fe2-BS346 (M_S = 2)**	18.7	14.0	31.4	26.0	2268	-164	-0.39	3.18	2.78	-2.77	-2.80	2.77	-1.96	2.63
**E_1-N_2@Fe2-BS147 (M_S = 1)**	15.1	4.6	27.8	16.6	2207	-225	-0.32	-3.16	2.36	2.96	-2.92	2.73	2.91	-2.48
**E_1-N_2@Fe2-BS235 (M_S = 1)**	22.7	13.6	35.4	25.6	2207	-225	-0.21	3.11	-2.36	-3.02	2.71	-2.86	2.14	2.35
**E_1-N_2@Fe2-BS247 (M_S = 1)**	25.4	16.9	38.1	28.9	2213	-219	-0.18	3.10	-2.38	2.69	-2.98	2.41	2.10	-2.78
**E_1-N_2@Fe2-BS346 (M_S = 1)**	13.8	5.2	26.5	17.2	2187	-245	-0.27	3.20	2.15	-2.87	-2.86	2.71	-2.80	2.61
**E_1-BS147 (M_S = 2)**	9.4	9.5					-0.42	-3.13	3.12	3.02	-2.77	2.85	2.92	-1.96
**E_1-BS235 (M_S = 2)**	1.8	1.6					-0.27	3.14	-2.71	-2.85	3.07	-2.27	2.59	2.72
**E_1-BS247 (M_S = 2)**	3.2	3.6					-0.28	3.11	-2.68	3.06	-2.84	2.77	2.52	-2.13
**E_1-BS346 (M_S = 2)**	0.0	0.0					-0.28	3.17	2.95	-2.86	-2.86	2.71	-1.93	2.60
**E_1-BS147 (M_S = 1)**	4.1	3.0					-0.38	-3.17	2.98	2.95	-3.02	2.76	2.53	-2.57
**E_1-BS235 (M_S = 1)**	4.3	3.1					-0.30	3.15	-2.76	-2.87	3.03	-2.83	1.78	2.56
**E_1-BS247 (M_S = 1)**	5.5	4.3					-0.23	3.12	-2.70	3.03	-2.86	2.60	1.68	-2.75
**E_1-BS346 (M_S = 1)**	8.0	7.4					-0.29	3.15	2.39	-2.91	-2.93	2.68	-2.71	2.53
**QM-VI region**														
**E_1-N_2@Fe2-BS147 (M_S = 1)**	0.0	1.3	9.4	11.2	2178	-254	-0.30	-3.15	2.38	2.94	-2.91	2.72	2.90	-2.47
**E_1-N_2@Fe6-BS346 (M_S = 2)**	0.2	0.0	9.5	9.9	2145	-287	-0.29	3.18	2.96	-2.86	-2.89	2.63	-1.82	2.53
**E_1-BS346 (M_S = 2)**							-0.25	3.16	2.94	-2.84	-2.85	2.69	-1.94	2.58

Table S16. Results of **E_2** models calculated using both QM-I and QM-VI regions. N_2 binding energies of **E_2-hyd** and **E_2-nonhyd** are relative to **E_2-hyd-SH^-@Fe6-BS235 (M_S = 3/2)** and **E_2-nonhyd-BS346 (M_S = 3/2)**, respectively. The single-step binding energies of **E_2-hyd-N_2@Fe6-BS235 (M_S = 1/2)** and **E_2-hyd-N_2@Fe2-BS235 (M_S = 3/2)** are relative to **E_2-hyd-SH^-@Fe2-BS235 (M_S = 3/2)** and **E_2-hyd-SH^-@Fe6-BS235 (M_S = 3/2)**, respectively.

QM-I region	ΔE(QM)	ΔE(QM/MM)	S. B. E. (QM)	S. B. E. (QMMM)	B. E. (QM)	B. E. (QMMM)	υ(N-N)	Rel. υ(N-N)	Mo	Fe1	Fe2	Fe3	Fe4	Fe5	Fe6	Fe7
E_2-nonhyd-N_2@Fe6-BS147 (M_S = 3/2)	9.9	5.9			21.9	14.6	2142	-290	-0.38	-3.12	2.98	2.86	-2.96	2.79	2.34	-1.53
E_2-nonhyd-N_2@Fe6-BS235 (M_S = 3/2)	0.0	0.0			12.0	8.7	2130	-302	-0.28	3.14	-2.09	-2.54	3.00	-2.82	2.35	2.04
E_2-nonhyd-N_2@Fe6-BS247 (M_S = 3/2)	4.3	2.3			16.3	10.9	2147	-285	-0.16	3.13	-2.76	2.90	-2.86	2.76	1.77	-2.02
E_2-nonhyd-N_2@Fe6-BS346 (M_S = 3/2)	0.3	0.3			12.3	9.0	2161	-271	-0.44	3.18	2.95	-2.71	-2.89	2.68	-1.95	1.76
E_2-nonhyd-N_2@Fe6-BS147 (M_S = 1/2)	9.0	3.1			21.1	11.7	2126	-306	-0.34	-3.11	2.88	2.82	-3.01	2.66	1.30	-2.01
E_2-nonhyd-N_2@Fe6-BS235 (M_S = 1/2)	6.8	1.9			18.8	10.6	2128	-304	-0.10	3.10	-2.74	-2.83	2.97	-2.78	1.44	1.99
E_2-nonhyd-N_2@Fe6-BS2467 (M_S = 1/2)	8.0	5.0			20.1	13.7	2133	-299	0.02	3.15	-2.94	2.85	-2.88	2.82	-0.81	-1.50
E_2-nonhyd-N_2@Fe6-BS346 (M_S = 1/2)	12.5	9.3			24.5	18.0	2153	-279	-0.36	3.11	1.62	-2.78	-2.90	2.59	-1.96	1.61
E_2-nonhyd-N_2@Fe2-BS147 (M_S = 3/2)	7.1	3.8			19.2	12.4	2195	-237	-0.42	-3.14	2.36	2.87	-2.91	2.72	3.03	-1.58
E_2-nonhyd-N_2@Fe2-BS235 (M_S = 3/2)	6.7	5.4			18.7	14.0	2190	-242	-0.29	3.17	-2.07	-2.92	2.97	-2.77	2.64	1.92
E_2-nonhyd-N_2@Fe2-BS247 (M_S = 3/2)	14.9	10.9			26.9	19.6	2188	-244	-0.18	3.13	-2.12	2.16	-3.03	2.66	2.41	-2.32
E_2-nonhyd-N_2@Fe2-BS346 (M_S = 3/2)	9.3	5.7			21.3	14.3	2175	-257	-0.43	3.15	2.20	-2.67	-2.84	2.82	-2.02	2.53
E_2-nonhyd-N_2@Fe2-BS147 (M_S = 1/2)	7.4	3.1			19.5	11.8	2177	-255	-0.37	-3.16	1.62	2.73	-3.04	2.73	2.73	-1.98
E_2-nonhyd-N_2@Fe2-BS2357 (M_S = 1/2)	9.1	7.0			21.1	15.6	2184	-248	0.25	3.15	-1.07	-2.89	3.03	-2.66	2.88	-2.18
E_2-nonhyd-N_2@Fe2-BS2347 (M_S = 1/2)	6.5	6.9			18.5	15.5	2194	-238	-0.40	3.20	-1.80	-2.79	-2.78	2.66	2.82	-0.37
E_2-nonhyd-N_2@Fe2-BS346 (M_S = 1/2)	7.8	3.7			19.8	12.3	2146	-286	-0.20	3.12	1.76	-2.77	-2.92	2.70	-2.86	2.44
E_2-hyd-N_2@Fe6-BS147 (M_S = 3/2)	14.2	15.2	6.1	3.6	16.0	16.3	2213	-219	-0.53	-3.11	2.48	3.04	-2.93	2.75	2.32	-1.11
E_2-hyd-N_2@Fe6-BS235 (M_S = 3/2)	6.1	5.7	-2.0	-5.9	7.9	6.8	2168	-264	-0.19	3.18	-2.00	-2.76	2.99	-2.67	1.46	2.68
E_2-hyd-N_2@Fe6-BS247 (M_S = 3/2)	6.3	6.6	-1.8	-5.0	8.1	7.6	2184	-248	-0.11	3.16	-2.15	2.96	-2.79	2.78	1.53	-2.59
E_2-hyd-N_2@Fe6-BS346 (M_S = 3/2)	5.8	6.8	-2.3	-4.8	7.6	7.8	2173	-259	-0.41	3.12	2.18	-3.02	-3.02	2.60	-1.08	2.43
E_2-hyd-N_2@Fe6-BS147 (M_S = 1/2)	4.2	4.5	-3.9	-7.1	6.0	5.6	2179	-253	-0.19	-3.13	2.44	2.86	-2.91	2.82	1.78	-2.49
E_2-hyd-N_2@Fe6-BS235 (M_S = 1/2)	0.0	0.0	-8.1	-11.6	1.8	1.1	2175	-257	0.02	3.13	-2.43	-2.83	3.00	-2.74	-0.14	2.69
E_2-hyd-N_2@Fe6-BS247 (M_S = 1/2)	3.7	3.1	-4.5	-8.5	5.5	4.1	2178	-254	0.04	3.13	-2.47	2.96	-2.84	2.75	-0.05	-2.66
E_2-hyd-N_2@Fe6-BS346 (M_S = 1/2)	13.2	13.3	5.1	1.7	15.0	14.4	2166	-266	-0.35	3.11	-0.12	-2.95	-2.85	2.60	-1.25	2.51
E_2-hyd-N_2@Fe2-BS147 (M_S = 3/2)	17.0	18.7	18.8	19.7	18.8	19.7	2223	-209	-0.49	-3.13	1.97	3.09	-2.93	2.75	2.97	-1.29
E_2-hyd-N_2@Fe2-BS235 (M_S = 3/2)	6.3	8.9	8.1	10.0	8.1	10.0	2219	-213	-0.24	3.16	-2.09	-2.95	2.93	-2.73	1.96	2.51
E_2-hyd-N_2@Fe2-BS247 (M_S = 3/2)	8.3	11.8	10.1	12.9	10.1	12.9	2206	-226	-0.20	3.14	-2.05	2.95	-2.93	2.60	1.68	-2.59
E_2-hyd-N_2@Fe2-BS346 (M_S = 3/2)	9.3	13.5	11.0	14.6	11.0	14.6	2249	-183	-0.17	3.15	2.60	-2.75	-2.82	2.67	-2.54	2.55
E_2-hyd-N_2@Fe2-BS147 (M_S = 1/2)	6.8	9.1	8.6	10.2	8.6	10.2	2248	-184	-0.33	-3.19	2.29	2.90	-2.95	2.73	2.29	-2.43
E_2-hyd-N_2@Fe2-BS235 (M_S = 1/2)	24.4	26.2	26.2	27.2	26.2	27.2	2216	-216	-0.27	1.78	-2.11	-2.93	2.54	-2.71	2.05	2.53
E_2-hyd-N_2@Fe2-BS2467 (M_S = 1/2)	9.5	12.3	11.3	13.4	11.3	13.4	2213	-219	0.21	3.15	-1.63	3.03	-2.82	2.79	-1.60	-2.56
E_2-hyd-N_2@Fe2-BS346 (M_S = 1/2)	9.2	11.5	11.0	12.6	11.0	12.6	2209	-223	-0.20	3.15	1.54	-2.93	-2.81	2.60	-2.75	2.49
E_2-nonhyd-BS147 (M_S = 3/2)	6.8	15.3							-0.44	-3.14	2.72	2.82	-3.02	2.78	2.83	-1.62
E_2-nonhyd-BS235 (M_S = 3/2)	9.1	15.9							-0.32	3.14	-2.23	-2.59	3.04	-2.79	2.39	1.98
E_2-nonhyd-BS247 (M_S = 3/2)	9.2	15.4							-0.25	3.08	-2.76	2.46	-2.95	2.77	2.44	-2.01
E_2-nonhyd-BS346 (M_S = 3/2)	4.0	13.6							-0.32	3.16	2.95	-2.74	-2.90	2.67	-1.98	1.83
E_2-nonhyd-BS147 (M_S = 1/2)	11.8	17.0							-0.51	-3.14	2.32	2.73	-3.04	2.71	2.34	-2.08
E_2-nonhyd-BS235 (M_S = 1/2)	9.5	17.7							-0.29	3.14	-2.61	-2.77	3.07	-2.80	1.42	1.72
E_2-nonhyd-BS247 (M_S = 1/2)	13.5	19.1							-1.06	3.09	-2.79	2.44	-2.96	2.84	2.54	-2.97
E_2-nonhyd-BS346 (M_S = 1/2)	8.4	17.6							-0.33	3.14	2.33	-2.87	-2.95	2.67	-2.75	1.87
E_2-hyd-SH^-@Fe6-BS147 (M_S = 3/2)	6.5	6.5							-0.41	-3.12	3.04	2.93	-2.85	2.79	3.09	-2.60
E_2-hyd-SH^-@Fe6-BS235 (M_S = 3/2)	0.8	0.0							-0.36	3.17	-2.75	-2.89	2.99	-2.78	2.67	2.51
E_2-hyd-SH^-@Fe6-BS247 (M_S = 3/2)	0.0	0.7							-0.29	3.14	-2.74	2.98	-2.88	2.60	2.64	-2.72
E_2-hyd-SH^-@Fe6-BS346 (M_S = 3/2)	2.1	1.5							-0.22	3.15	2.97	-2.81	-2.82	2.75	-2.99	2.63
E_2-hyd-SH^-@Fe6-BS147 (M_S = 1/2)	5.1	2.7							-0.35	-3.17	2.58	2.77	-3.03	2.75	2.36	-2.58
E_2-hyd-SH^-@Fe6-BS235 (M_S = 1/2)	18.9	17.4							-0.37	1.81	-2.80	-2.90	2.70	-2.77	2.69	2.54
E_2-hyd-SH^-@Fe6-BS247 (M_S = 1/2)	21.1	19.1							-0.33	2.16	-2.82	2.37	-2.95	2.64	2.69	-2.68
E_2-hyd-SH^-@Fe6-BS346 (M_S = 1/2)	14.1	13.1							-0.14	3.15	2.32	-3.07	-3.02	2.52	-3.05	2.47
E_2-hyd-SH^-@Fe2-BS147 (M_S = 3/2)	15.1	17.2							-0.37	-3.16	2.77	2.95	-2.92	2.80	2.82	-2.02
E_2-hyd-SH^-@Fe2-BS235 (M_S = 3/2)	10.8	12.6							-0.24	3.17	-2.62	-2.83	2.98	-2.78	2.38	2.63
E_2-hyd-SH^-@Fe2-BS247 (M_S = 3/2)	13.2	14.9							-0.19	3.16	-2.65	2.94	-2.82	2.75	2.34	-2.72
E_2-hyd-SH^-@Fe2-BS346 (M_S = 3/2)	9.5	13.1							-0.29	3.18	2.93	-2.87	-2.89	2.63	-2.55	2.52
E_2-hyd-SH^-@Fe2-BS147 (M_S = 1/2)	11.0	12.8							-0.35	-3.16	2.40	2.89	-3.02	2.76	2.43	-2.67
E_2-hyd-SH^-@Fe2-BS2356 (M_S = 1/2)	19.6	20.5							0.08	3.14	-2.25	-2.68	3.08	-2.65	-0.96	2.76
E_2-hyd-SH^-@Fe2-BS2467 (M_S = 1/2)	22.6	24.4							0.07	3.15	-2.34	3.06	-2.73	2.82	-0.83	-2.51
E_2-hyd-SH^-@Fe2-BS346 (M_S = 1/2)	22.1	24.6							-0.39	3.13	1.94	-2.93	-2.87	2.58	-2.91	2.48
**QM-VI region**																
E_2-hyd-N_2@Fe6-BS235 (M_S = 1/2)	0.0	0.0	-9.5	-9.8	3.5	1.9	2175	-257	0.01	3.12	-2.44	-2.81	2.99	-2.75	-0.08	2.67
E_2-hyd-N_2@Fe2-BS235 (M_S = 3/2)	3.6	3.5	7.1	5.4	7.1	5.4	2198	-234	-0.24	3.15	-2.10	-2.93	2.93	-2.72	1.92	2.51
E_2-nonhyd-N_2@Fe6-BS235 (M_S = 3/2)	15.2	18.8			4.1	8.5	2130	-302	-0.32	3.13	-2.15	-2.53	2.98	-2.80	2.32	2.14
E_2-nonhyd-N_2@Fe2-BS147 (M_S = 1/2)	15.4	16.5			4.3	6.2	2146	-286	-0.35	-3.16	1.76	2.74	-3.02	2.72	2.71	-2.08
E_2-nonhyd-BS346 (M_S = 3/2)	14.6	12.1							-0.26	3.15	2.96	-2.70	-2.89	2.66	-1.96	1.70
E_2-hyd-SH^-@Fe6-BS235 (M_S = 3/2)	0.0	0.0							-0.35	3.16	-2.74	-2.86	2.97	-2.77	2.66	2.50
E_2-hyd-SH^-@Fe2-BS235 (M_S = 3/2)	13.0	11.7							-0.25	3.16	-2.63	-2.81	2.96	-2.77	2.35	2.63

Table S17. Results of **E_4** models calculated using both QM-I and QM-VI regions. N_2 binding energies of **E_4** models are relative to **E_4-SP-SH^-@Fe6-BS346 (M_S = 3/2)**. The single-step binding energies of **E_4-SP-N_2@Fe6**, **E_4-SP-N_2@Fe2**, **E_4-DP-Fe2/6(3)**, and **E_4-DP-Fe2/6(5)** are relative to states **E_4-SP-SH^-@Fe2-BS147 (M_S = 1/2)**, **E_4-SP-SH^-@Fe6-BS346 (M_S = 3/2)**, **E_4-DP-Fe2/6(3)-BS135 (M_S = 1/2)**, **E_4-DP-Fe2/6(5)-BS147 (M_S = 1/2)**, respectively.

QM-I region	△E(QM)	△E(QM/MM)	S. B. E. (QM)	S. B. E. (QMMM)	B. E. (QM)	B. E. (QMMM)	υ(N-N)	Rel. υ(N-N)	Mo	Fe1	Fe2	Fe3	Fe4	Fe5	Fe6	Fe7
E_4-SP-N_2@Fe6-BS147 (M_S = 3/2)	16.6	17.9	3.2	1.9	13.0	13.4	2180	-252	-0.13	-2.47	3.02	2.91	-2.81	2.83	0.56	-1.42
E_4-SP-N_2@Fe6-BS235 (M_S = 3/2)	13.7	11.1	0.3	-4.9	10.0	6.6	2180	-252	0.05	3.13	-1.45	-1.81	3.07	-2.73	-0.46	2.51
E_4-SP-N_2@Fe6-BS247 (M_S = 3/2)	6.6	7.3	-6.8	-8.7	2.9	2.8	2185	-247	-0.13	3.23	-2.01	2.89	-2.80	2.86	-0.23	-1.44
E_4-SP-N_2@Fe6-BS346 (M_S = 3/2)	8.2	7.2	-5.2	-8.8	4.6	2.7	2176	-256	-0.64	3.13	0.62	-2.61	-2.84	2.75	-0.29	2.51
E_4-SP-N_2@Fe6-BS147 (M_S = 1/2)	3.9	5.9	-9.4	-10.1	0.3	1.4	2181	-251	-0.11	-3.12	2.70	2.77	-2.93	2.75	0.65	-1.57
E_4-SP-N_2@Fe6-BS235 (M_S = 1/2)	5.2	5.0	-8.2	-11.1	1.5	0.4	2187	-245	0.11	3.16	-2.28	-2.34	3.01	-2.75	-0.43	2.14
E_4-SP-N_2@Fe6-BS247 (M_S = 1/2)	1.5	2.6	-11.9	-13.5	-2.1	-2.0	2179	-253	0.12	3.17	-2.55	2.81	-2.85	2.64	-0.62	-2.10
E_4-SP-N_2@Fe6-BS234 (M_S = 1/2)	0.0	0.0	-13.4	-16.1	-3.6	-4.6	2180	-252	-0.63	3.19	-1.82	-2.56	-2.74	2.75	-0.07	2.53
E_4-SP-N_2@Fe2-BS147 (M_S = 3/2)	11.0	14.7	7.3	10.1	7.3	10.1	2245	-187	0.54	-3.10	1.10	2.81	-2.98	2.86	3.30	-1.75
E_4-SP-N_2@Fe2-BS235 (M_S = 3/2)	13.4	16.1	9.8	11.5	9.8	11.5	2229	-203	0.05	3.11	-0.26	-2.79	3.03	-2.77	0.70	1.61
E_4-SP-N_2@Fe2-BS247 (M_S = 3/2)	6.3	11.0	2.7	6.4	2.7	6.4	2222	-210	-0.12	3.17	-1.72	2.91	-2.91	2.75	-0.13	-1.60
E_4-SP-N_2@Fe2-BS346 (M_S = 3/2)	10.8	12.9	7.1	8.4	7.1	8.4	2200	-232	-0.38	3.14	1.14	-2.71	-2.88	2.70	-0.48	2.36
E_4-SP-N_2@Fe2-BS147 (M_S = 1/2)	3.9	7.6	0.3	3.1	0.3	3.1	2238	-194	-0.42	-3.09	1.20	2.78	-3.05	2.67	2.73	-1.54
E_4-SP-N_2@Fe2-BS2356 (M_S = 1/2)	6.6	8.6	3.0	4.1	3.0	4.1	2222	-210	0.28	3.13	-1.30	-2.47	3.08	-2.68	-1.56	1.96
E_4-SP-N_2@Fe2-BS247 (M_S = 1/2)	6.2	10.8	2.6	6.3	2.6	6.3	2237	-195	0.27	3.16	-1.48	2.87	-2.91	2.65	-2.13	-1.94

**E$_4$-SP-N$_2$@Fe2-BS346 (M_S = 1/2)**	2.0	5.2	-1.6	0.7	-1.6	0.7	2217	-215	-0.21	3.19	-0.14	-2.67	-2.82	2.75	-1.93	2.47
**E$_4$-DP-Fe2/6(3)-N$_2$@Fe6-BS14 (M_S = 3/2)**	17.3	12.0	8.6	-0.7	13.7	7.4	2164	-268	-0.84	-3.09	2.32	1.96	-3.04	2.63	1.01	2.47
**E$_4$-DP-Fe2/6(3)-N$_2$@Fe6-BS235 (M_S = 3/2)**	21.3	15.3	12.6	2.6	17.7	10.7	2160	-272	-0.27	3.19	-1.87	-2.74	2.98	-2.70	1.45	2.66
**E$_4$-DP-Fe2/6(3)-N$_2$@Fe6-BS247 (M_S = 3/2)**	21.6	15.8	12.9	3.1	18.0	11.3	2173	-259	-0.03	3.18	-2.00	2.86	-2.80	2.75	1.43	-2.63
**E$_4$-DP-Fe2/6(3)-N$_2$@Fe6-BS346 (M_S = 3/2)**	18.5	14.2	9.8	1.5	14.9	9.7	2152	-280	-0.36	3.13	2.30	-2.75	-2.83	2.75	-0.92	1.33
**E$_4$-DP-Fe2/6(3)-N$_2$@Fe6-BS135 (M_S = 1/2)**	18.3	15.3	9.5	2.6	14.6	10.7	2162	-270	-0.31	-3.13	2.90	-2.61	2.85	-2.54	1.16	2.72
**E$_4$-DP-Fe2/6(3)-N$_2$@Fe6-BS147 (M_S = 1/2)**	20.5	15.3	11.7	2.6	16.8	10.8	2164	-268	-0.14	-3.15	2.42	2.83	-2.95	2.77	1.79	-2.29
**E$_4$-DP-Fe2/6(3)-N$_2$@Fe6-BS235 (M_S = 1/2)**	15.6	10.3	6.9	-2.5	12.0	5.7	2166	-266	-0.04	3.15	-2.43	-2.81	3.00	-2.71	-0.15	2.66
**E$_4$-DP-Fe2/6(3)-N$_2$@Fe6-BS247 (M_S = 1/2)**	21.8	16.6	13.1	3.9	18.2	12.0	2157	-275	-0.11	3.22	-2.06	1.26	-2.79	2.66	1.21	-2.52
**E$_4$-DP-Fe2/6(3)-N$_2$@Fe6-BS346 (M_S = 1/2)**	26.5	20.8	17.8	8.1	22.9	16.2	2148	-284	-0.38	3.15	-0.16	-2.71	-2.72	2.79	-0.92	1.55
**E$_4$-DP-Fe2/6(3)-N$_2$@Fe2-BS14 (M_S = 3/2)**	18.6	17.3	9.8	4.6	15.0	12.7	2217	-215	-0.85	-3.14	1.51	2.54	-3.06	2.63	2.05	1.79
**E$_4$-DP-Fe2/6(3)-N$_2$@Fe2-BS235 (M_S = 3/2)**	19.0	17.7	10.3	5.0	15.4	13.1	2206	-226	-0.19	3.15	-1.15	-2.86	2.96	-2.73	2.47	0.95
**E$_4$-DP-Fe2/6(3)-N$_2$@Fe2-BS247 (M_S = 3/2)**	21.5	20.7	12.8	8.0	17.9	16.2	2212	-220	-0.02	3.14	-1.89	2.84	-2.90	2.67	1.48	-2.70
**E$_4$-DP-Fe2/6(3)-N$_2$@Fe2-BS346 (M_S = 3/2)**	31.3	32.3	22.5	19.6	27.7	27.8	2198	-234	-0.04	3.20	1.62	-0.07	-2.87	2.68	-2.41	0.61
**E$_4$-DP-Fe2/6(3)-N$_2$@Fe2-BS135 (M_S = 1/2)**	16.1	16.2	7.4	3.5	12.5	11.7	2179	-253	-0.46	-3.14	2.09	-2.90	2.90	-2.51	2.63	2.60
**E$_4$-DP-Fe2/6(3)-N$_2$@Fe2-BS147 (M_S = 1/2)**	18.2	17.3	9.4	4.6	14.6	12.8	2208	-224	-0.40	-3.16	1.61	2.80	-3.06	2.67	1.92	-1.06
**E$_4$-DP-Fe2/6(3)-N$_2$@Fe2-BS156 (M_S = 1/2)**	13.6	15.1	4.8	2.4	10.0	10.6	2216	-216	0.38	-3.18	1.92	2.97	2.82	-2.56	-2.20	1.18
**E$_4$-DP-Fe2/6(3)-N$_2$@Fe2-BS157 (M_S = 1/2)**	15.2	14.9	6.5	2.2	11.6	10.3	2208	-224	0.17	-3.18	1.41	2.74	2.74	-2.70	1.66	-1.59
**E$_4$-DP-Fe2/6(3)-N$_2$@Fe2-BS2357 (M_S = 1/2)**	16.4	16.6	7.7	3.9	12.8	12.0	2201	-231	0.18	3.15	-1.35	-2.77	3.05	-2.73	2.61	-1.69
**E$_4$-DP-Fe2/6(3)-N$_2$@Fe2-BS234 (M_S = 1/2)**	15.1	15.4	6.3	2.7	11.4	10.8	2207	-225	-0.70	3.18	-1.94	-2.66	-2.78	2.73	1.85	1.00
**E$_4$-DP-Fe2/6(3)-N$_2$@Fe2-BS346 (M_S = 1/2)**	19.0	19.1	10.3	6.4	15.4	14.5	2187	-245	-0.12	3.15	1.51	-2.78	-2.88	2.67	-1.99	1.52
**E$_4$-DP-Fe2/6(5)-N$_2$@Fe6-BS147 (M_S = 3/2)**	40.8	30.6	24.2	20.2	37.2	26.0	2167	-265	-0.14	-3.07	2.94	2.76	-0.74	2.72	1.20	-2.56
**E$_4$-DP-Fe2/6(5)-N$_2$@Fe6-BS235 (M_S = 3/2)**	25.9	14.1	9.3	3.8	22.3	9.6	2158	-274	-0.28	3.19	-1.87	-2.76	2.97	-2.70	1.46	2.69
**E$_4$-DP-Fe2/6(5)-N$_2$@Fe6-BS247 (M_S = 3/2)**	25.7	14.6	9.1	4.2	22.1	10.0	2170	-262	-0.02	3.17	-1.99	2.87	-2.79	2.74	1.42	-2.66
**E$_4$-DP-Fe2/6(5)-N$_2$@Fe6-BS346 (M_S = 3/2)**	23.8	14.2	7.2	3.9	20.2	9.7	2174	-258	-0.45	3.11	2.17	-3.04	-3.02	2.53	-1.04	2.52
**E$_4$-DP-Fe2/6(5)-N$_2$@Fe6-BS147 (M_S = 1/2)**	24.8	14.5	8.2	4.1	21.2	9.9	2172	-260	-0.22	-3.16	2.82	2.86	-2.98	2.70	1.30	-2.05
**E$_4$-DP-Fe2/6(5)-N$_2$@Fe6-BS235 (M_S = 1/2)**	21.1	9.0	4.5	-1.3	17.5	4.5	2164	-268	-0.05	3.15	-2.43	-2.82	2.99	-2.71	-0.17	2.69
**E$_4$-DP-Fe2/6(5)-N$_2$@Fe6-BS247 (M_S = 1/2)**	18.1	7.8	1.5	-2.5	14.5	3.3	2180	-252	0.14	3.14	-2.45	2.81	-2.81	2.74	-0.03	-2.71
**E$_4$-DP-Fe2/6(5)-N$_2$@Fe6-BS2346 (M_S = 1/2)**	21.1	10.2	4.5	-0.2	17.5	5.7	2177	-255	-0.64	3.17	-2.17	-2.67	-2.61	2.89	-0.29	2.86
**E$_4$-DP-Fe2/6(5)-N$_2$@Fe2-BS14 (M_S = 3/2)**	22.6	15.7	6.0	5.3	19.0	11.1	2214	-218	-0.66	-3.15	1.88	3.04	-3.00	2.46	2.27	0.44
**E$_4$-DP-Fe2/6(5)-N$_2$@Fe2-BS235 (M_S = 3/2)**	23.5	15.8	6.9	5.4	19.9	11.2	2221	-211	-0.34	3.18	-2.13	-2.93	2.90	-2.71	2.00	2.55
**E$_4$-DP-Fe2/6(5)-N$_2$@Fe2-BS247 (M_S = 3/2)**	25.1	17.7	8.5	7.4	21.5	13.2	2206	-226	-0.12	3.15	-1.94	2.88	-2.89	2.60	1.53	-2.65
**E$_4$-DP-Fe2/6(5)-N$_2$@Fe2-BS346 (M_S = 3/2)**	30.3	21.6	13.7	11.2	26.7	17.0	2201	-231	-0.34	3.15	1.66	-2.64	-2.78	2.77	-1.86	2.74
**E$_4$-DP-Fe2/6(5)-N$_2$@Fe2-BS147 (M_S = 1/2)**	24.2	16.2	7.6	5.8	20.6	11.7	2236	-196	-0.32	-3.20	2.09	2.86	-3.00	2.65	2.50	-2.20
**E$_4$-DP-Fe2/6(5)-N$_2$@Fe2-BS157 (M_S = 1/2)**	19.9	12.7	3.3	2.3	16.3	8.1	2215	-217	0.24	-3.19	1.29	2.68	2.75	-2.63	2.74	-2.61
**E$_4$-DP-Fe2/6(5)-N$_2$@Fe2-BS2356 (M_S = 1/2)**	24.0	14.7	7.3	4.3	20.3	10.1	2211	-221	0.19	3.16	-1.31	-2.78	3.05	-2.59	-2.00	2.69
**E$_4$-DP-Fe2/6(5)-N$_2$@Fe2-BS2467 (M_S = 1/2)**	25.1	17.2	8.5	6.8	21.5	12.7	2214	-218	0.32	3.15	-1.59	2.96	-2.77	2.77	-1.67	-2.64
**E$_4$-DP-Fe2/6(5)-N$_2$@Fe2-BS346 (M_S = 1/2)**	26.0	18.2	9.4	7.8	22.4	13.7	2209	-223	-0.29	3.16	1.50	-2.83	-2.78	2.59	-2.81	2.50
**E$_4$-SP-SH$^-$@Fe2-BS14 (M_S = 3/2)**	14.1	15.6							-0.79	-3.16	2.34	2.87	-3.11	2.50	1.89	0.86
**E$_4$-SP-SH$^-$@Fe2-BS235 (M_S = 3/2)**	18.3	16.8							-0.14	3.15	-2.57	-2.69	2.99	-2.39	1.71	2.53
**E$_4$-SP-SH$^-$@Fe2-BS247 (M_S = 3/2)**	12.4	13.5							-0.22	3.17	-2.65	2.93	-2.85	2.71	1.39	-1.86
**E$_4$-SP-SH$^-$@Fe2-BS346 (M_S = 3/2)**	14.2	13.2							-0.24	3.11	2.33	-2.80	-2.98	2.65	-1.42	2.28
**E$_4$-SP-SH$^-$@Fe2-BS147 (M_S = 1/2)**	11.1	11.5							-0.40	-3.15	2.03	2.72	-3.05	2.72	2.22	-1.74
**E$_4$-SP-SH$^-$@Fe2-BS235 (M_S = 1/2)**	16.1	16.1							-0.06	3.15	-2.45	-2.62	3.06	-2.68	0.30	2.01
**E$_4$-SP-SH$^-$@Fe2-BS2467 (M_S = 1/2)**	10.6	12.2							0.20	3.19	-2.47	2.90	-2.90	2.79	-1.40	-1.81
**E$_4$-SP-SH$^-$@Fe6-BS147 (M_S = 3/2)**	3.4	3.3							-0.55	-3.14	2.39	2.81	-2.89	2.87	3.03	-1.61
**E$_4$-SP-SH$^-$@Fe6-BS235 (M_S = 3/2)**	12.5	12.0							-0.23	3.16	-2.30	-2.74	3.10	-1.94	0.79	2.60
**E$_4$-SP-SH$^-$@Fe6-BS247 (M_S = 3/2)**	4.5	5.8							-0.23	3.20	-2.38	3.00	-2.84	2.81	0.32	-1.42
**E$_4$-SP-SH$^-$@Fe6-BS346 (M_S = 3/2)**	1.4	0.0							-0.15	3.10	2.48	-2.65	-2.86	2.77	-2.47	2.52
**E$_4$-SP-SH$^-$@Fe6-BS147 (M_S = 1/2)**	2.4	3.4							-0.33	-3.15	2.51	2.65	-3.06	2.78	1.47	-1.49
**E$_4$-SP-SH$^-$@Fe6-BS2356 (M_S = 1/2)**	4.2	3.2							0.23	3.16	-2.49	-2.11	3.10	-2.78	-1.21	2.47
**E$_4$-SP-SH$^-$@Fe6-BS2467 (M_S = 1/2)**	0.0	1.7							0.21	3.16	-2.49	2.96	-2.82	2.74	-1.38	-1.98
**E$_4$-SP-SH$^-$@Fe6-BS346 (M_S = 1/2)**	0.8	0.6							-0.37	3.12	2.16	-2.73	-2.85	2.69	-2.94	2.00
**E$_4$-DP-Fe2/6(3)-BS14 (M_S = 3/2)**	8.8	11.2							-0.63	-3.17	2.82	2.33	-3.06	2.62	-0.18	2.58
**E$_4$-DP-Fe2/6(3)-BS235 (M_S = 3/2)**	10.8	13.1							-0.24	3.18	-2.72	-2.79	3.10	-2.25	1.37	2.74
**E$_4$-DP-Fe2/6(3)-BS247 (M_S = 3/2)**	12.5	14.9							-0.24	3.13	-2.71	3.01	-2.83	2.84	1.57	-2.11
**E$_4$-DP-Fe2/6(3)-BS347 (M_S = 3/2)**	10.0	13.6							-0.11	3.18	3.05	-2.85	-2.91	2.74	0.66	-1.21
**E$_4$-DP-Fe2/6(3)-BS135 (M_S = 1/2)**	6.5	8.2							-0.39	-3.17	2.74	-2.88	2.87	-2.63	1.93	2.74
**E$_4$-DP-Fe2/6(3)-BS14 (M_S = 1/2)**	11.6	14.1							-0.44	-3.19	2.60	2.55	-3.21	2.57	0.03	0.65
**E$_4$-DP-Fe2/6(3)-BS235 (M_S = 1/2)**	10.5	12.6							-0.16	3.19	-2.83	-2.82	3.05	-2.74	0.56	2.41
**E$_4$-DP-Fe2/6(3)-BS247 (M_S = 1/2)**	12.0	13.4							-0.07	3.14	-2.86	2.75	-2.81	2.71	0.82	-2.75
**E$_4$-DP-Fe2/6(3)-BS345 (M_S = 1/2)**	8.5	10.3							-0.34	3.18	2.90	-2.94	-3.10	-2.84	1.48	2.70
**E$_4$-DP-Fe2/6(3)-BS3467 (M_S = 1/2)**	8.7	11.9							0.12	3.16	2.95	-2.57	-2.96	2.76	-0.67	-2.20
**E$_4$-DP-Fe2/6(5)-BS147 (M_S = 3/2)**	30.9	20.9							-0.28	-1.83	2.73	2.91	-2.82	2.74	1.95	-2.45
**E$_4$-DP-Fe2/6(5)-BS235 (M_S = 3/2)**	17.2	10.2							-0.45	3.23	-2.72	-2.87	2.95	-2.74	2.42	2.68
**E$_4$-DP-Fe2/6(5)-BS247 (M_S = 3/2)**	17.2	9.2							-0.17	3.12	-2.71	3.02	-2.79	2.77	1.37	-2.04
**E$_4$-DP-Fe2/6(5)-BS346 (M_S = 3/2)**	13.5	8.1							-0.34	3.20	1.99	-2.78	-2.81	2.68	-2.02	2.70
**E$_4$-DP-Fe2/6(5)-BS147 (M_S = 1/2)**	14.4	5.8							-0.28	-3.22	2.73	2.90	-3.03	2.75	1.96	-2.42
**E$_4$-DP-Fe2/6(5)-BS235 (M_S = 1/2)**	19.6	10.9							-0.22	3.20	-2.86	-2.84	2.98	-2.78	0.77	2.49
**E$_4$-DP-Fe2/6(5)-BS247 (M_S = 1/2)**	17.1	8.8							-0.01	3.13	-2.85	2.90	-2.79	2.65	0.57	-2.77
**E$_4$-DP-Fe2/6(5)-BS234 (M_S = 1/2)**	21.0	13.5							-0.59	3.20	-2.19	-2.65	-2.64	2.78	-0.16	2.77
**QM-VI region**																
**E$_4$-SP-N$_2$@Fe6-BS234 (M_S = 1/2)**	0.8	0.0	-15.0	-15.2	-2.3	-4.1	2178	-254	-0.61	3.18	-1.77	-2.59	-2.73	2.73	-0.11	2.55
**E$_4$-SP-N$_2$@Fe2-BS346 (M_S = 1/2)**	0.0	0.1	-3.1	-4.0	-3.1	-4.0	2191	-241	-0.18	3.18	-0.13	-2.70	-2.80	2.73	-1.96	2.52
**E$_4$-DP-Fe2/6(3)-N$_2$@Fe2-BS2357 (M_S = 1/2)**	14.8	16.0	-5.2	-5.0	11.7	11.9	2190	-242	0.14	3.16	-1.58	-2.72	3.05	-2.69	2.64	-1.62
**E$_4$-DP-Fe2/6(3)-N$_2$@Fe2-BS156 (M_S = 1/2)**	14.4	16.1	-5.6	-4.9	11.3	11.9	2192	-240	0.39	-3.19	1.95	2.96	2.80	-2.55	-2.12	1.12
**E$_4$-DP-Fe2/6(3)-N$_2$@Fe2-BS157 (M_S = 1/2)**	16.1	17.7	-4.0	-3.2	13.0	13.6	2167	-265	0.15	-3.17	1.37	2.71	2.74	-2.75	1.64	-1.34
**E$_4$-DP-Fe2/6(3)-N$_2$@Fe2-BS345 (M_S = 1/2)**	25.0	27.2	5.0	6.2	21.9	23.1	2160	-272	-0.28	3.12	1.65	-2.79	-2.97	-1.54	2.47	1.54
**E$_4$-DP-Fe2/6(3)-N$_2$@Fe6-BS235 (M_S = 1/2)**	16.8	17.5	-3.3	-3.4	13.6	13.5	2160	-272	-0.04	3.14	-2.40	-2.72	2.99	-2.70	-0.20	2.55
**E$_4$-DP-Fe2/6(5)-N$_2$@Fe6-BS2467 (M_S = 1/2)**	19.2	19.3	1.0	-2.2	16.1	15.2	2136	-296	0.19	3.17	-2.36	2.84	-2.82	2.44	-1.19	-1.70
**E$_4$-DP-Fe2/6(5)-N$_2$@Fe2-BS235 (M_S = 1/2)**	14.8	16.0	-3.4	-5.5	11.7	11.9	2190	-242	0.14	3.16	-1.58	-2.72	3.05	-2.69	2.63	-1.62
**E$_4$-DP-Fe2/6(5)-N$_2$@Fe2-BS157 (M_S = 1/2)**	16.0	17.8	-2.2	-3.7	12.9	13.7	2167	-265	0.15	-3.17	1.37	2.71	2.73	-2.75	1.64	-1.34
**E$_4$-SP-SH$^-$@Fe2-BS147 (M_S = 1/2)**	12.6	11.1							-0.40	-3.13	1.94	2.73	-3.03	2.70	2.31	-1.76
**E$_4$-SP-SH$^-$@Fe6-BS346 (M_S = 3/2)**	0.0	0.0							-0.19	3.10	2.14	-2.65	-2.83	2.81	-2.28	2.58
**E$_4$-SP-SH$^-$@Fe6-BS346 (M_S = 1/2)**	3.9	2.9							-0.32	3.13	2.13	-2.72	-2.82	2.67	-2.98	1.96
**E$_4$-SP-SH$^-$@Fe6-BS2467 (M_S = 1/2)**	1.3	1.7							0.23	3.15	-2.44	2.97	-2.80	2.73	-1.41	-2.01
**E$_4$-DP-Fe2/6(3)-BS14 (M_S = 3/2)**	19.1	17.5							-0.82	-3.15	2.31	2.67	-3.04	2.67	2.40	0.32
**E$_4$-DP-Fe2/6(3)-BS14 (M_S = 1/2)**	21.6	19.8							-0.45	-3.18	2.57	2.52	-3.20	2.57	0.13	0.61
**E$_4$-DP-Fe2/6(3)-BS346 (M_S = 1/2)**	22.0	20.3							-0.18	3.15	1.38	-2.78	-2.86	2.77	-1.93	1.30
**E$_4$-DP-Fe2/6(3)-BS135 (M_S = 1/2)**	17.0	16.9							-0.41	-3.14	2.70	-2.80	2.86	-2.63	1.96	2.67
**E$_4$-DP-Fe2/6(3)-BS345 (M_S = 1/2)**	19.0	18.8							-0.36	3.16	2.86	-2.88	-3.09	-2.83	1.55	2.63
**E$_4$-DP-Fe2/6(5)-BS147 (M_S = 1/2)**	15.1	17.4							-0.31	-3.21	2.68	2.81	-3.03	2.73	1.98	-2.25

References

(1) Foster, J. M.; Boys, S. F. Canonical Configurational Interaction Procedure. *Reviews of Modern Physics* **1960**, *32*, 300-302.

(2) Knizia, G. Intrinsic Atomic Orbitals: An Unbiased Bridge between Quantum Theory and Chemical Concepts. *Journal of chemical theory and computation* **2013**, *9*, 4834-4843.

(3) Benediktsson, B.; Bjornsson, R. Analysis of the Geometric and Electronic Structure of Spin-Coupled Iron-Sulfur Dimers with Broken-Symmetry DFT: Implications for FeMoco. *Journal of chemical theory and computation* **2022**, *18*, 1437-1457.

(4) Harris, T. V.; Szilagyi, R. K. Comparative assessment of the composition and charge state of nitrogenase FeMo-cofactor. *Inorganic chemistry* **2011**, *50*, 4811-4824.

(5) Cao, L.; Ryde, U. What Is the Structure of the E4 Intermediate in Nitrogenase? *Journal of chemical theory and computation* **2020**, *16*, 1936-1952.

(6) Dance, I. New insights into the reaction capabilities of His(195) adjacent to the active site of nitrogenase. *J Inorg Biochem* **2017**, *169*, 32-43.

(7) Thorhallsson, A. T.; Bjornsson, R. The E2 state of FeMoco: Hydride Formation versus Fe Reduction and a Mechanism for H2 Evolution. *Chemistry – A European Journal* **2021**, *27*, 16788-16800.

(8) Fryzuk, M. D. Side-on End-on Bound Dinitrogen: An Activated Bonding Mode That Facilitates Functionalizing Molecular Nitrogen. *Accounts of Chemical Research* **2009**, *42*, 127-133.

(9) Fryzuk, M. D. N2 coordination. *Chemical communications* **2013**, *49*, 4866-4868.

(10) Burford, R. J.; Fryzuk, M. D. Examining the relationship between coordination mode and reactivity of dinitrogen. *Nature Reviews Chemistry* **2017**, *1*.

(11) Dance, I. Structures and reaction dynamics of N2 and H2 binding at FeMo-co, the active site of nitrogenase. *Dalton transactions* **2021**, *50*, 18212-18237.
